# Supplementary material for: Clostridium perfringens virulence factors are nonredundant activators of the NLRP3 inflammasome
Source: EMBO Rep. 2023 Apr 19;24(6):e54600. doi: 10.15252/embr.202254600 (PMC10240202; doi:10.15252/embr.202254600)
Supplement: Supplementary file 1 — Appendix [file EMBR-24-e54600-s006.docx]

**Appendix Data**

***Clostridium perfringens* virulence factors are non-redundant activators of the NLRP3 inflammasome**

**Table of contents**

Appendix Figure S1. Lecithinase induces cell death in macrophages………………………2

Appendix Figure S2. Lecithinase-induced inflammasome activation

following AF568 labelling is not affected..………………………………………………….3

Appendix Figure S3. Production of inflammasome-independent cytokines

by macrophages following treatment with inhibitors is not affected……………….……….5

Appendix Figure S4. Cytosolic access of PFO to induce activation of

the inflammasome is not required.…… ………………………….…………………………6

Appendix Figure S5. Lecithinase-induced inflammasome activation is

inhibited by concanamycin A…………………………………………………………….….7

Appendix Figure S6. Priming of macrophages following treatment with

bafilomycin A and ammonium chloride is not affected.…………………………………….8

Appendix Figure S7. Lecithinase-induced lysosomal destabilization

precedes cell death…………………………………………………………………………...9

Appendix Figure S8. Lecithinase-mediated inflammasome activation is dependent on K+ efflux……………………………………………………………………………………...…11

Appendix Figure S9. Caspase-8 is not required for lecithinase mediated

inflammasome activation…….……………………………………………………………...13

**
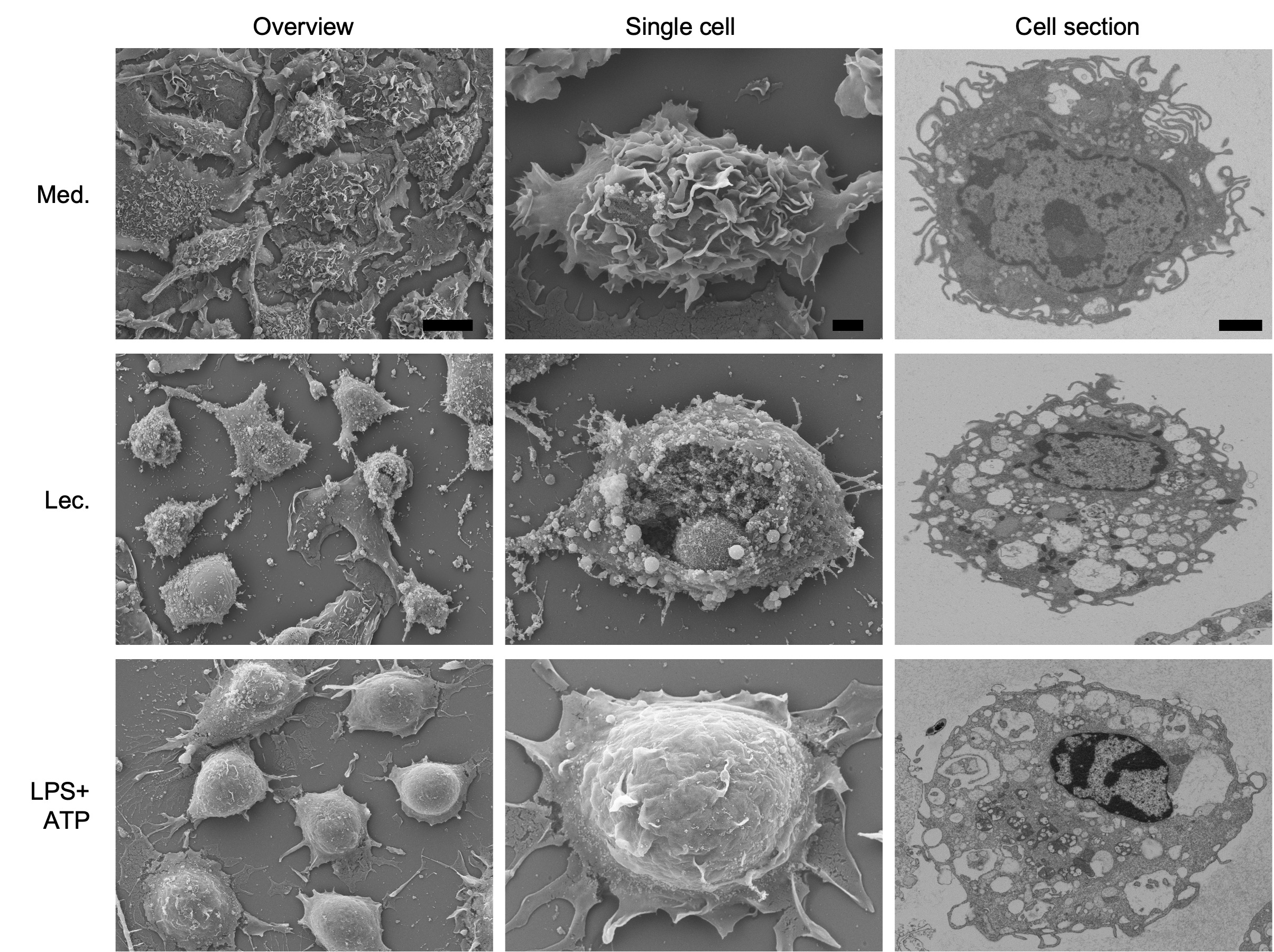
**

**Appendix Figure S1. Lecithinase induces cell death in macrophages.** Scanning electron microscopy and transmission electron microscopy analyses of WT BMDMs left untreated [Medium alone (Med.)] or LPS-primed and assessed 3 hr after stimulation with lecithinase (Lec.) or 1 hr after stimulation with ATP (LPS+ATP). Left panel shows overview SEM image, middle panel shows single cell SEM image and right panel shows TEM cell section image. Scale bar 10 μm (overview); 2 μm (single cell and cell section). Data are representative of one experiment.

**
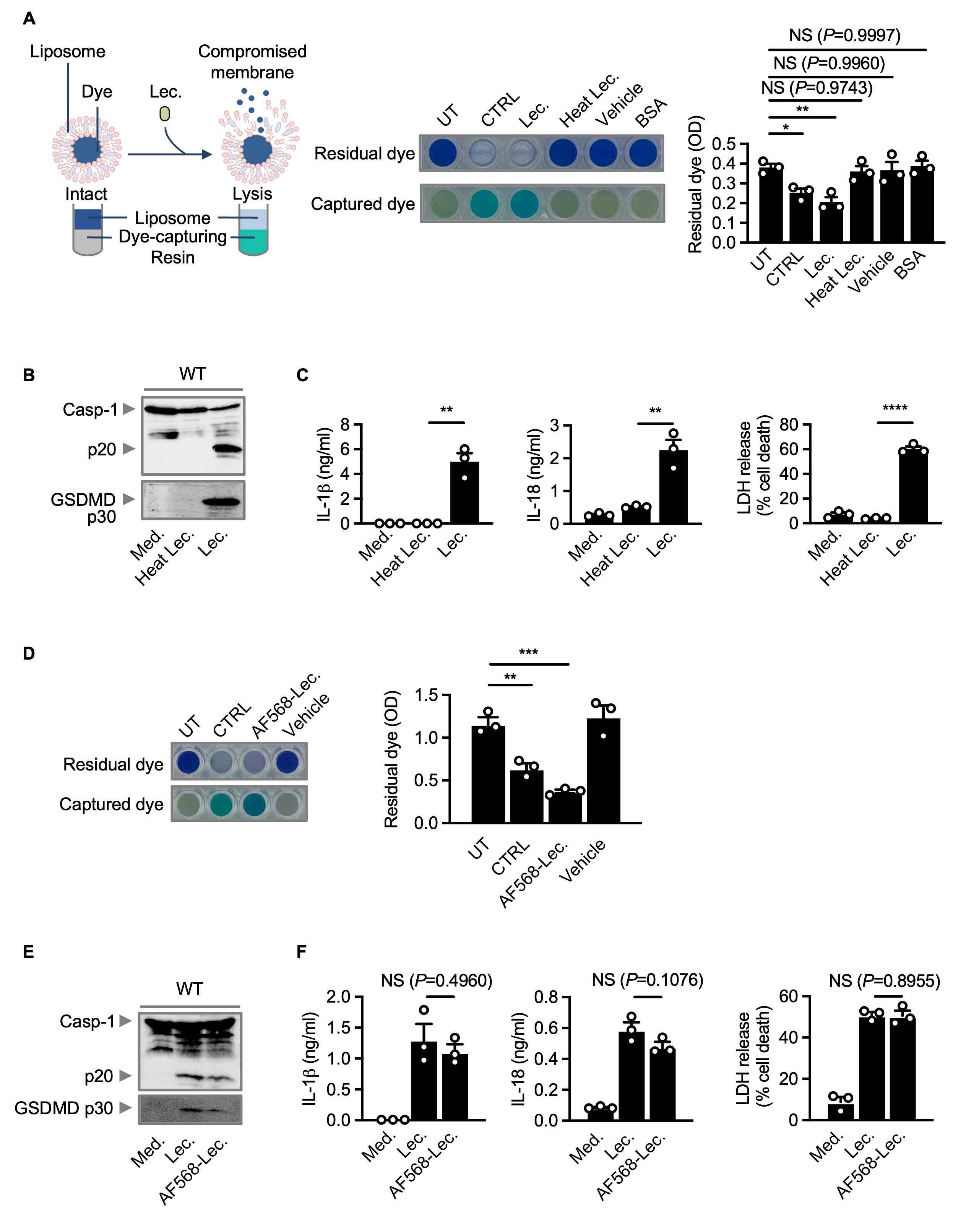
**

**Appendix Figure S2. Lecithinase-induced inflammasome activation following AF568 labelling is not affected.** (**A**) Colorimetric analysis of liposomes left untreated (UT), sonicated for 5 min at 100 amplitude as positive control (CTRL), or assessed 5 min after stimulation with lecithinase (Lec.), heat treated lecithinase (Heat Lec.), vehicle buffer (vehicle) or BSA. The absorbance (OD) of residual dye was measured at 595 nm. (**B**) Immunoblot analysis of caspase-1 and GSDMD of WT BMDMs left untreated [Medium alone (Med.)] or LPS-primed and assessed 3 hr after stimulation with lecithinase (Lec.) or lecithinase heated to 100 °C for 10 min (Heat Lec.). (**C**) Release of IL-1β (left) and IL-18 (middle), and death (right) of WT BMDMs as treated in B. (**D**) Colorimetric analysis of liposomes left untreated (UT), sonicated for 5 min at 100 amplitude as positive control (CTRL), or assessed 10 min after stimulation with AF568 labelled lecithinase (AF568-Lec.) or vehicle buffer. The absorbance (OD) of residual dye was measured at 595 nm. (**E**) Immunoblot analysis of caspase-1 and gasdermin D of WT BMDMs left untreated or LPS-primed and assessed 4 hr after stimulation with unlabelled lecithinase (Lec.) or AF568 labelled lecithinase (AF568-Lec.). (**F**) Release of IL-1β (left) and IL-18 (middle), and death (right) of WT BMDMs as treated in E. Each symbol represents an independent biological replicate (A, C, D and F). NS, not significant, **P* < 0.05, ***P* < 0.01 and ****P* < 0.001 (one-way ANOVA with Dunnett’s multiple-comparisons test [A and D] or two-tailed *t*-test [C and F]). Data are representative of three biological independent experiments (A to F; mean and s.e.m. in A, C, D and F).

**
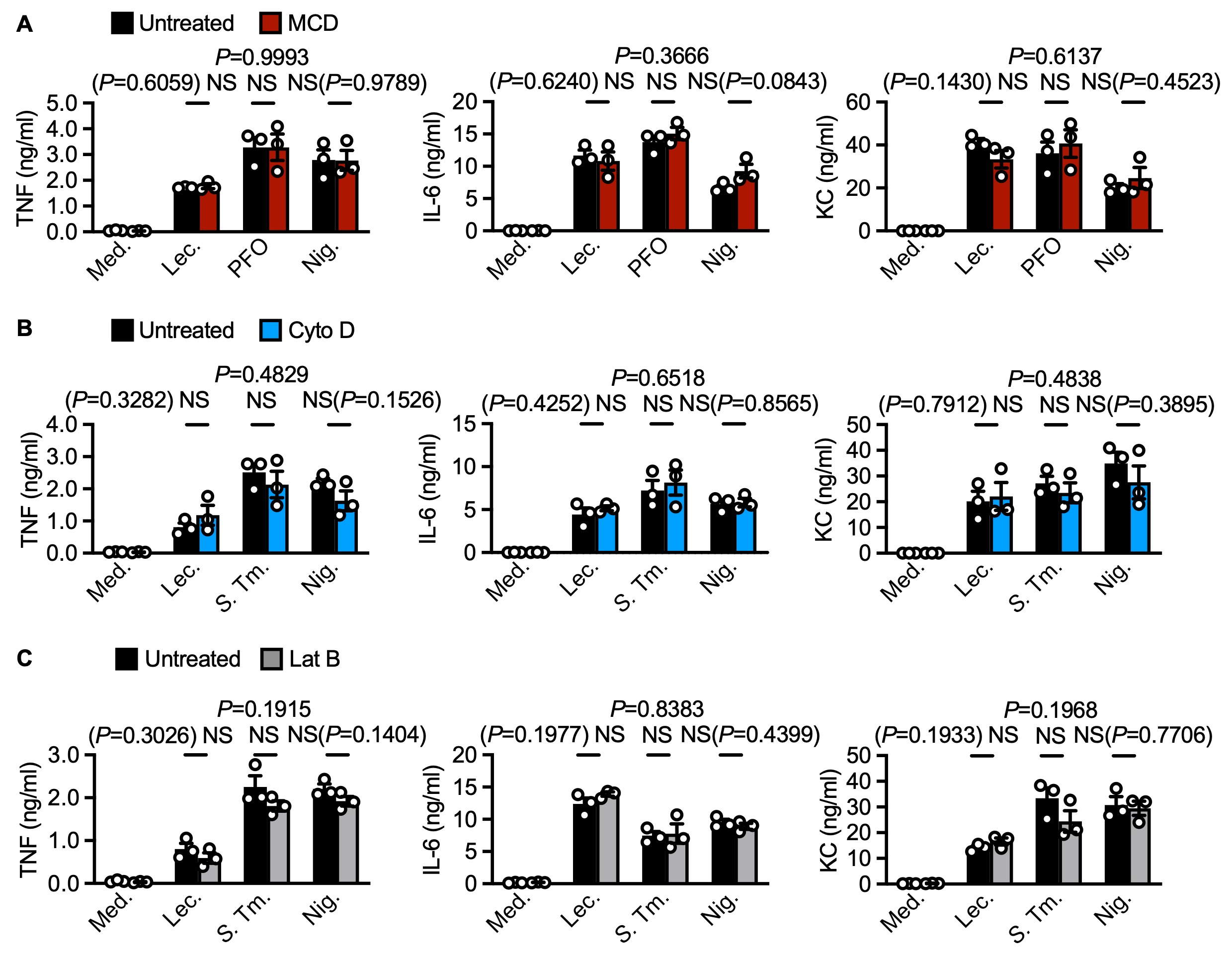
**

**Appendix Figure S3. Production of inflammasome-independent cytokines by macrophages following treatment with inhibitors is not affected.** (**A** to **C**) Release of TNF (left), IL-6 (middle) and KC (right) from WT BMDMs left untreated [Medium alone (Med.)] or assessed 3 hr after stimulation with lecithinase (Lec.), or 3 hr after stimulation with perfringolysin O (PFO), or 30 min after stimulation with nigericin (Nig.), or 4 hr after infection of with *S.* Typhimurium (*S.* Tm.; MOI, 5), or 16 hr after infection with *F. novicida* (*F. novi.,* MOI 100), in the absence or presence of inhibitors (Cytochalasin D [Cyto D], 50μM; latrunculin B [Lat B], 1 μg/ml; methyl-β-cyclodextrin [MCD], 5mM. Each symbol represents an independent biological replicate (A to C). NS, not significant (two-tailed *t*-test [A to C]). Data are representative of three biological independent experiments (A to C; mean and s.e.m. in A to C).

**
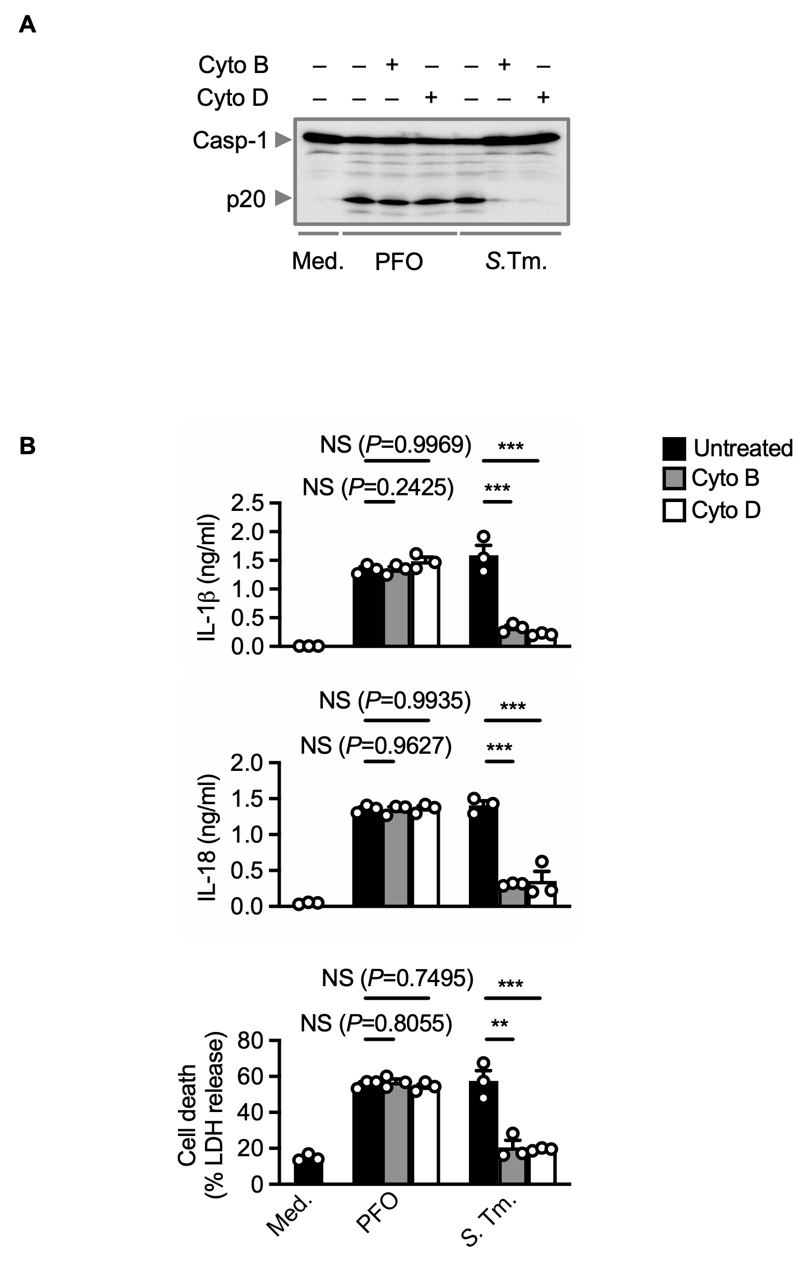
**

**Appendix Figure S4. Cytosolic access of PFO to induce activation of the inflammasome is not required.** (**A**) Immunoblot analysis of caspase-1 of WT BMDMs left untreated [Medium alone (Med.)] or LPS-primed and assessed 3 hr after stimulation with perfringolysin O (PFO) or 4 hr after infection of with *S.* Typhimurium (*S.* Tm.; MOI, 5) in the absence or presence of Cytochalasin D [Cyto D] or Cytochalasin B [Cyto B], 50μM. (**B**) Release of IL-1β, IL-18 and death of WT BMDMs as treated in A. Each symbol represents an independent biological replicate (B). NS, not significant. ***P* < 0.01, ****P* < 0.001 (one-way ANOVA with Dunnett’s multiple-comparisons test [B]). Data are representative of three biological independent experiments (A and B; mean and s.e.m. in B).

**
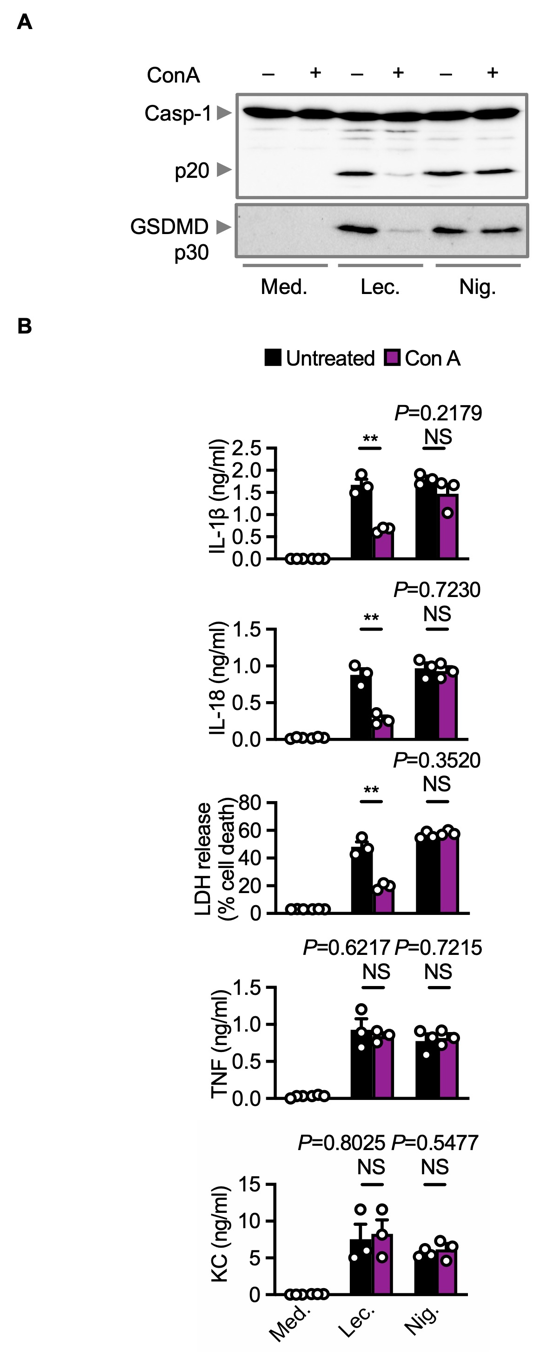
**

**Appendix Figure S5. Lecithinase-induced inflammasome activation is inhibited by concanamycin A.** (**A**) Immunoblot analysis of caspase-1 and gasdermin D of WT BMDMs left untreated [Medium alone (Med.)] or LPS-primed and assessed 3 hr after stimulation with lecithinase (Lec.) or 30 min after stimulation with nigericin (Nig.) in the absence or presence of concanamycin A (100 nM). (**B**) Release of IL-1β, IL-18, death, TNF and KC of WT BMDMs as treated in A. Each symbol represents an independent biological replicate (B). NS, not significant. ***P* < 0.01 (two-tailed *t*-test [B]). Data are representative of three biological independent experiments (A and B; mean and s.e.m. in B).

**
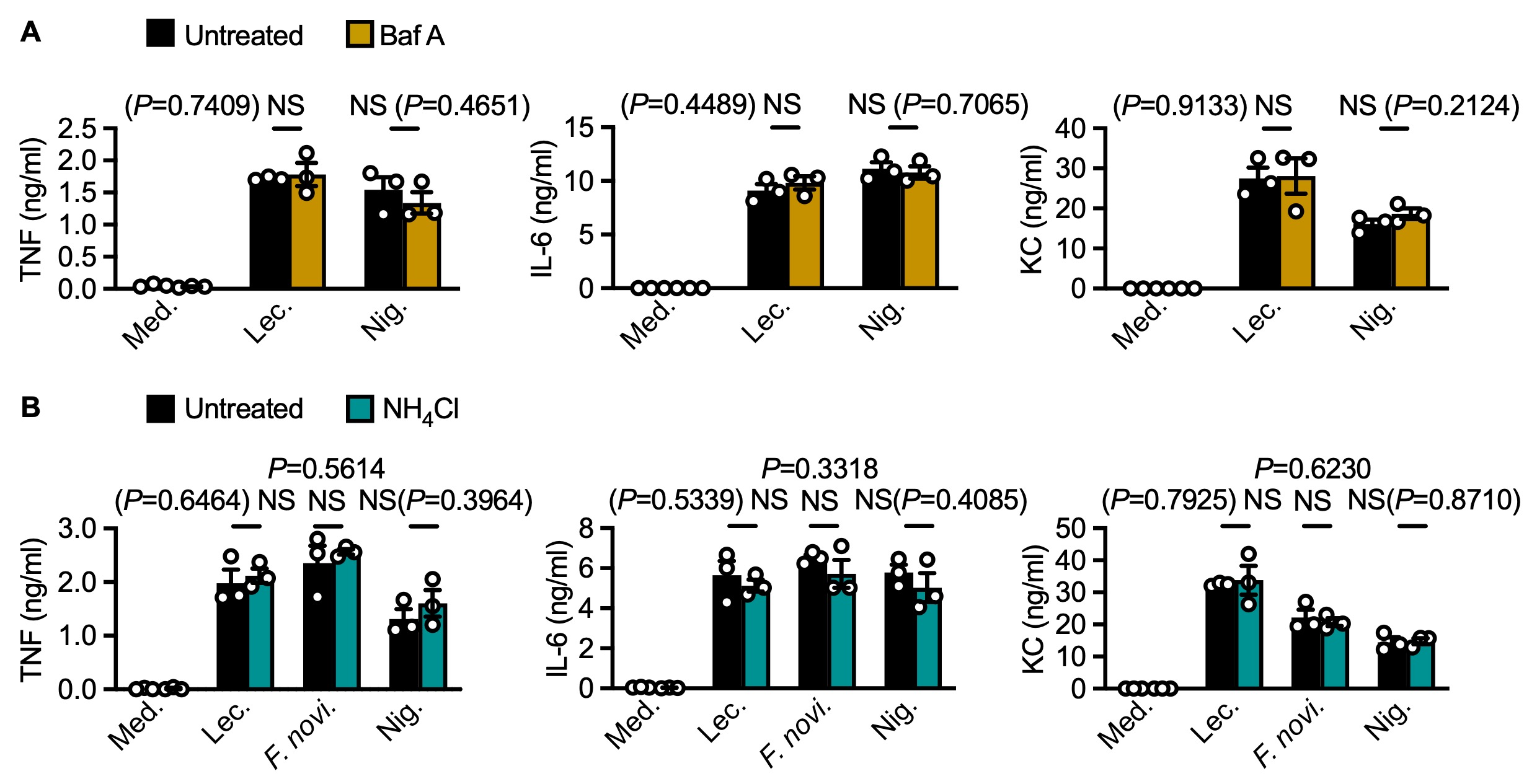
**

**Appendix Figure S6. Priming of macrophages following treatment with bafilomycin A and ammonium chloride is not affected.** (**A** and **B**) Release of TNF (left), IL-6 (middle) and KC (right) from WT BMDMs left untreated [Medium alone (Med.)] or assessed 3 hr after stimulation with lecithinase (Lec.), or 30 min after stimulation with nigericin (Nig.), or 16 hr after infection with *F. novicida* (*F. novi.,* MOI 100), in the absence or presence of inhibitors bafilomycin A [Baf A], 100nM and ammonium chloride [NH_4_Cl] 20μM. Each symbol represents an independent biological replicate (A and B). NS, not significant (two-tailed *t*-test [A and B). Data are representative of three biological independent experiments (A and B; mean and s.e.m. in A and B).

**
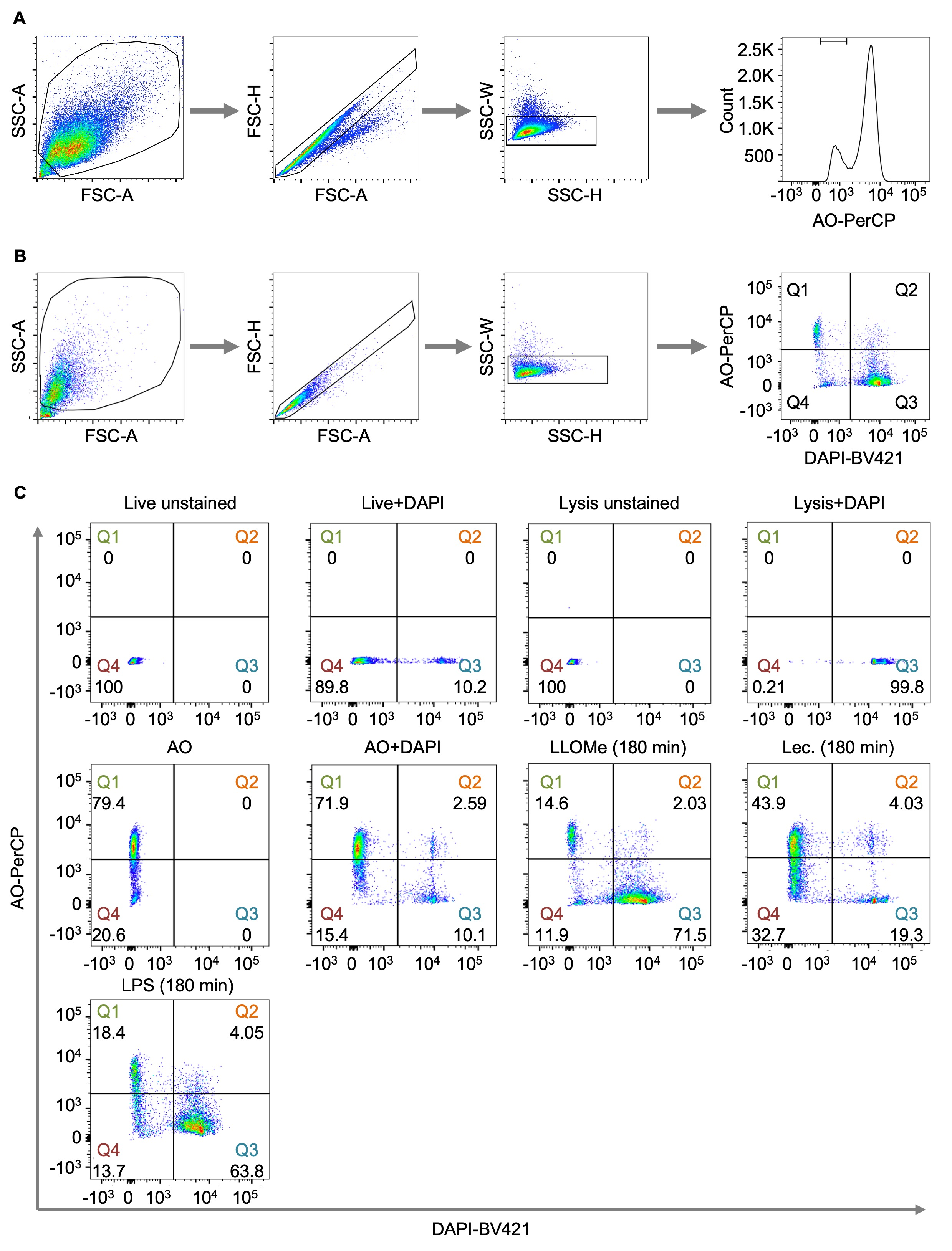
**

**Appendix Figure S7. Lecithinase-induced lysosomal destabilization precedes cell death.** (**A**) Flow cytometry scatter plots illustrating the gating strategy used to detect the loss of acridine orange fluorescence. Single and viable cells were selected based on the side and forward scatter. From the single cells, the loss of acridine orange fluorescence was recorded at 600–650 nm. (**B**) Flow cytometry scatter plots illustrating the gating strategy used to assess the loss of acridine orange dye from lysosomes (the PerCP channel) and DAPI^+^ or dead cells (the BV421 channel). (**C**) Flow cytometry scatter plots illustrating the percentage of cell population in various quadrants left untreated (Live) or lysed using 0.2% saponin (Lysis) or assessed 180 min after treatment with either lecithinase (Lec.) or LLOMe or LPS transfection (LPS) in the presence or absence of labelling dyes (acridine orange (AO) and DAPI). The loss of acridine orange dye (the PerCP channel) was used to evaluate lysosomal destabilization and DAPI (the BV421 channel) was used to evaluate cell viability (DAPI^+^ cells indicate cell death).

**Appendix Figure S8. Lecithinase-mediated inflammasome activation is dependent on K^+^ efflux.** (**A**) Inductively coupled plasma-optical emission spectrometry analysis of intracellular concentrations of K^+^ of BMDMs left untreated [Medium alone (Med.)] or LPS primed and assessed 2 hr after stimulation with lecithinase (Lec.), or 30 min after stimulation with ATP. (**B**) Immunoblot analysis of caspase-1 and gasdermin D in WT BMDMs left untreated or LPS primed and assessed 3 hr after stimulation with lecithinase (Lec.) or 1 hr after stimulation with ATP in the absence (–) or presence of 50 mM KCl (+) or at increasing concentrations of KCl (wedge; 5, 25, 50 and 75 mM). (**C**) Release of IL-1β (top) and IL-18 (middle), and death (bottom) of WT BMDMs after treatment as in B. (**D**) Inductively coupled plasma-optical emission spectrometry analysis of intracellular concentrations of K^+^ of BMDMs left untreated or LPS primed and assessed 2 hr after stimulation with lecithinase (Lec.), or 30 min after stimulation with nigericin (Nig.) in the absence or presence of cytochalasin D (Cyto D, 50μM), bafilomycin A (Baf A, 100nM) and methyl-β-cyclodextrin (MCD, 5mM). Each symbol represents an independent biological replicate (A, C and D). NS, not significant. ***P* < 0.01, ****P* < 0.001 and *****P* < 0.0001 (one-way analysis of variance [ANOVA] with Dunnett’s multiple-comparisons test [A, C and D]). Data are representative of four biological independent experiments (A) or three biological independent experiments (B to D; mean and s.e.m. in A, C and D).

**
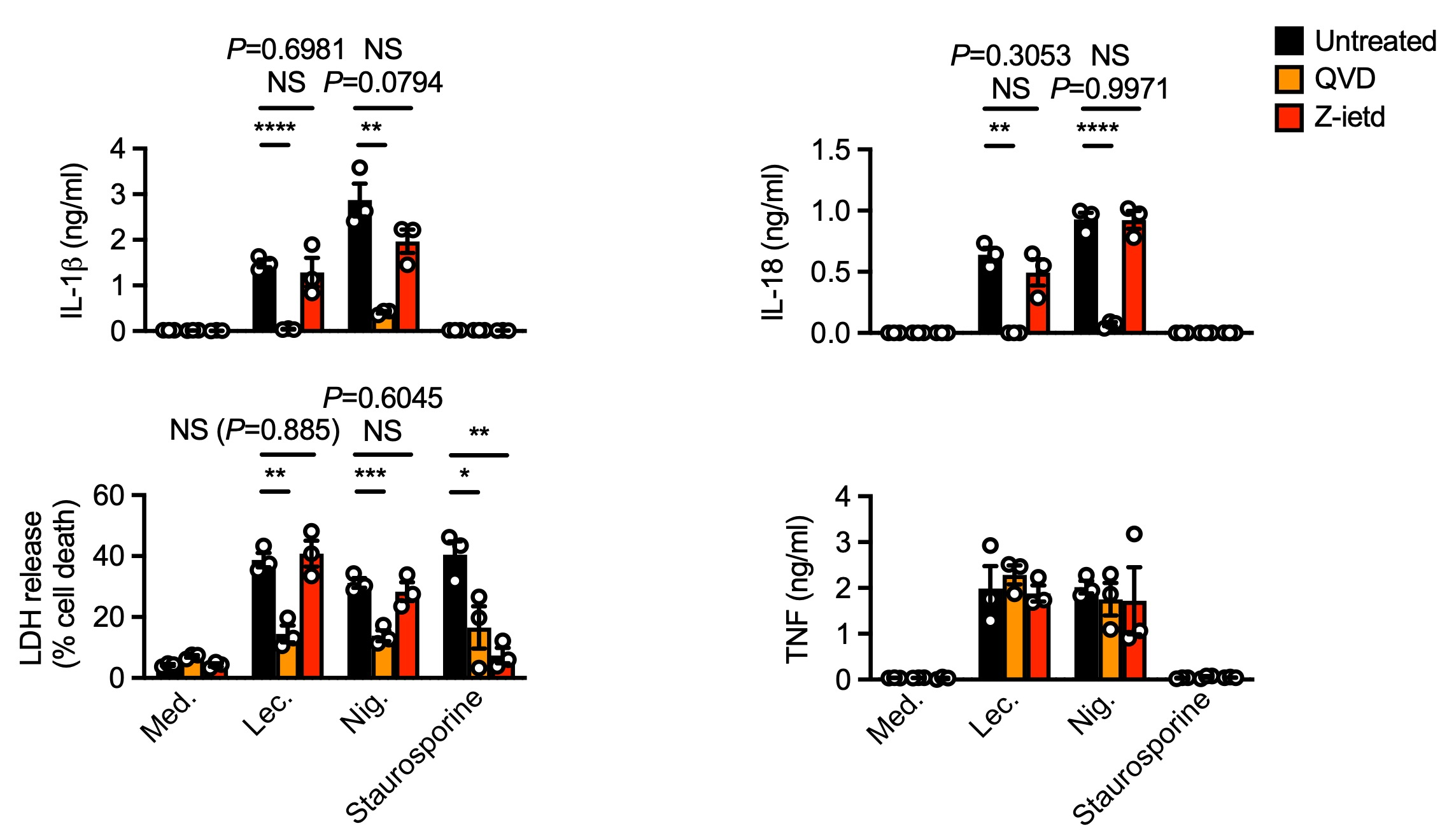
**

**Appendix Figure S9. Caspase-8 is not required for lecithinase mediated inflammasome activation.**

Release of IL-1β, IL-18, death and TNF of WT BMDMs left untreated [Medium alone (Med.)] or LPS-primed and assessed 3 hr after stimulation with lecithinase (Lec.) or 30 min after stimulation with nigericin (Nig.) or 12 hr after stimulation with Staurosporine in the absence or presence of Q-VD-OPh hydrate (QVD, 20 µM) or Z-IETD-FMK (Z-ietd, 20 µ M). Each symbol represents an independent biological replicate. NS, not significant. (one-way analysis of variance [ANOVA] with Dunnett’s multiple-comparisons test). Data are representative of three biological independent experiments (mean and s.e.m.).
