# Supplementary material for: Clostridium perfringens virulence factors are nonredundant activators of the NLRP3 inflammasome
Source: EMBO Rep. 2023 Apr 19;24(6):e54600. doi: 10.15252/embr.202254600 (PMC10240202; doi:10.15252/embr.202254600)
Supplement: Supplementary file 2 — Expanded View Figures PDF [file EMBR-24-e54600-s005.pdf]

Expanded View Figures

Figure EV1. PFO activates the NLRP3 inflammasome.

A Immunoblot analysis of caspase-1 (Casp-1) in WT or *Nlrp3*$^{-/-}$ BMDMs left untreated (Medium alone [Med.]) or LPS primed and assessed 4 h after stimulation with bacteria or the supernatant (Sup.) of WT *C. perfringens* (WT *C. per.*), *ΔpfoA C. perfringens* (*ΔpfoA*) or *Δcpa C. perfringens* (*Δcpa*).
B Release of IL-1β (top) and IL-18 (middle), and death (bottom) of BMDMs as treated in (A).
C Immunoblot analysis of caspase-1 in WT or *Nlrp3*$^{-/-}$ BMDMs left untreated or LPS primed and assessed 3 h after stimulation with perfringolysin O (PFO).
D Release of IL-1β (top) and IL-18 (middle), and death (bottom) of BMDMs as treated in (C).

Data information: Each symbol represents an independent biological replicate (B and D). NS, not significant, *$P < 0.05$, and ****$P < 0.0001$ (one-way ANOVA with Dunnett's multiple-comparisons test [B] or two-tailed t-test [D]). Data are representative of three independent biological experiments (A–D; mean and s.e.m. in B and D).
Source data are available online for this figure.

© 2023 The Authors

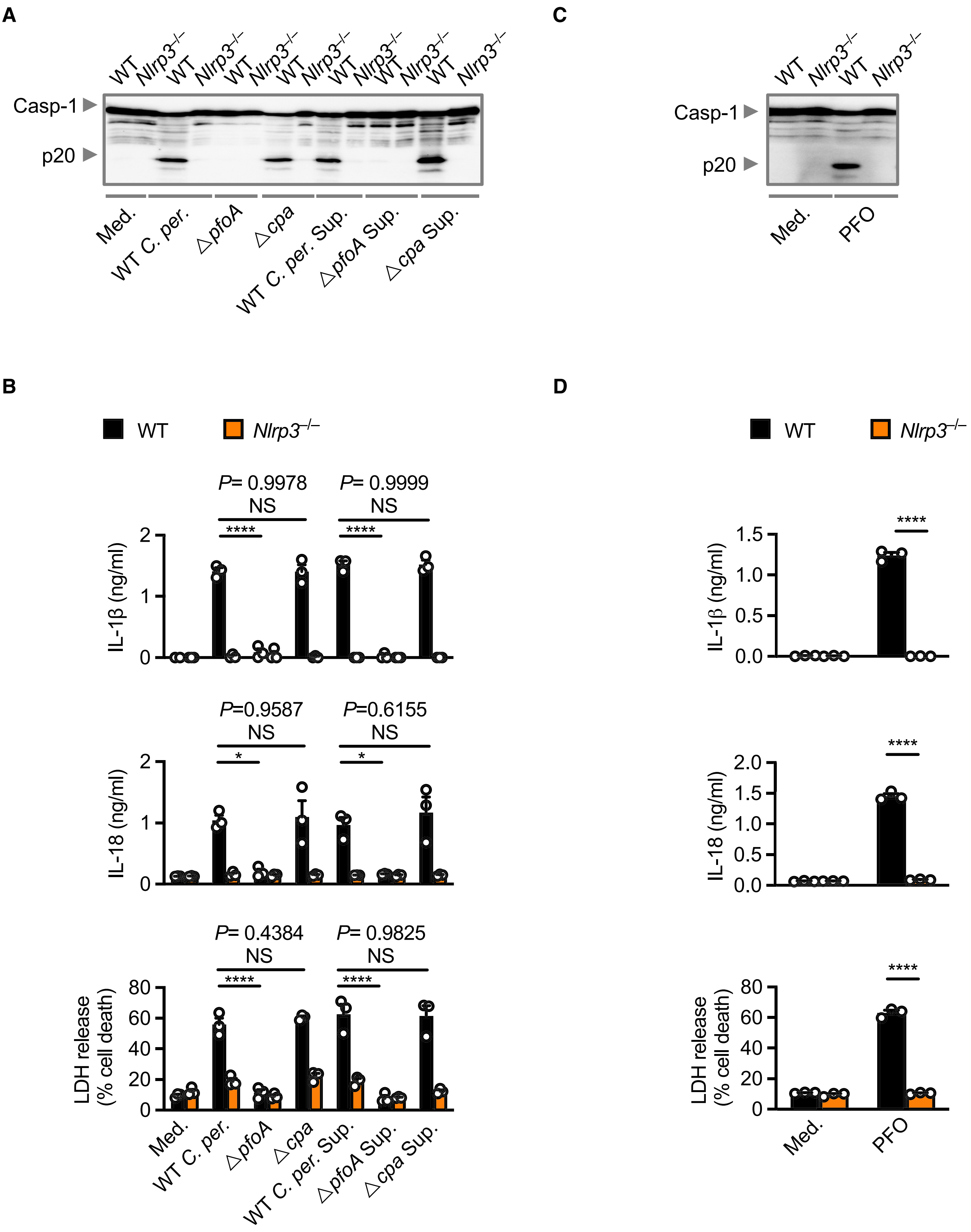

Figure EV1.

 © 2023 The Authors

Figure EV2. Lecithinase activates the NLRP3 inflammasome in murine cells.

A Microscopy analysis of death of BMDMs left untreated [Medium alone (Med.)] or LPS-primed and assessed 3 h after stimulation with lecithinase (Lec.). Arrowheads indicate dead cells.
B IncuCyte live-imaging analysis of the viability of WT or *Nlrp3*$^{-/-}$ BMDMs left untreated or LPS-primed and assessed after stimulation with lecithinase (Lec.) or ATP.
C Release of IL-6 and KC of BMDMs left untreated or LPS-primed and assessed 3 h after stimulation with lecithinase (Lec.).
D Immunoblot analysis of caspase-1 and gasdermin D of WT or *Nlrp3*$^{-/-}$ BMDMs left untreated or LPS-primed and assessed 12 h after stimulation with 0.1 mg/ml an alternatively sourced lecithinase (Cusabio).
E Release of IL-1β (left) and IL-18 (middle), and death (right) of BMDMs after treatment as in (D).
F Concentration of TNF and KC in the serum of WT mice, 8 h after intraperitoneal infection with 4×10^8 colony-forming units (CFUs) of *C. perfringens* (WT *C. perf.*, *n* = 14), *ΔpfoA C. perfringens* (*ΔpfoA*, *n* = 13), *Δcpa C. perfringens* (*Δcpa*, *n* = 11) or *ΔpfoAΔcpa C. perfringens* (*ΔpfoAΔcpa*, *n* = 12).

Data information: Each symbol represents an independent biological replicate (C and E) or represents an individual mouse (F). NS, not significant, $^{**}P < 0.01$, $^{***}P < 0.001$ and $^{****}P < 0.0001$ (two-tailed *t*-test [B and E] or one-way ANOVA with Dunnett's multiple-comparisons test [F]). Data are pooled from two independent biological experiments (B and F) or representative of three independent biological experiments (A, C, D and E; mean and s.e.m. in B, C, E and F).
Source data are available online for this figure.

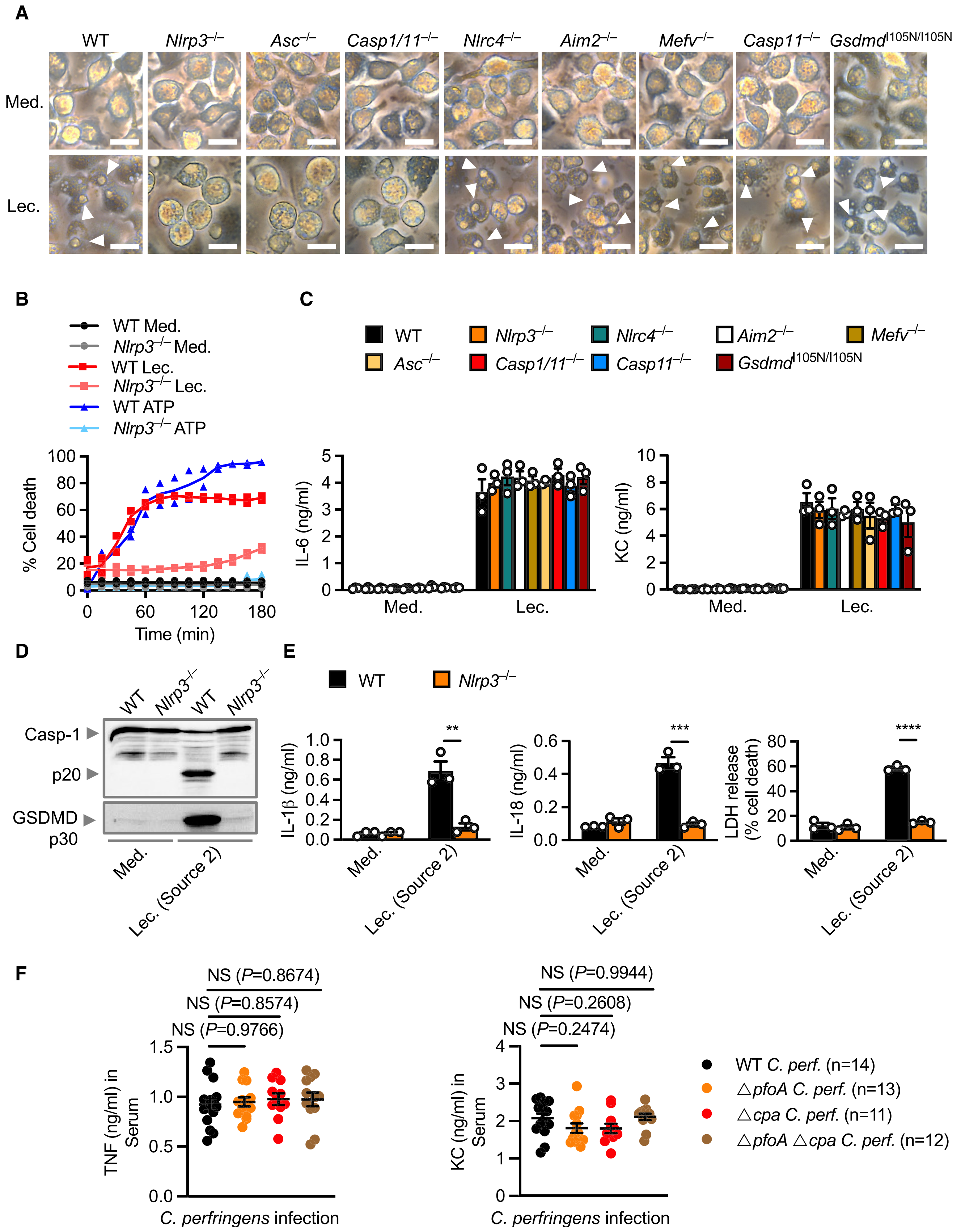

Figure EV2.

© 2023 The Authors

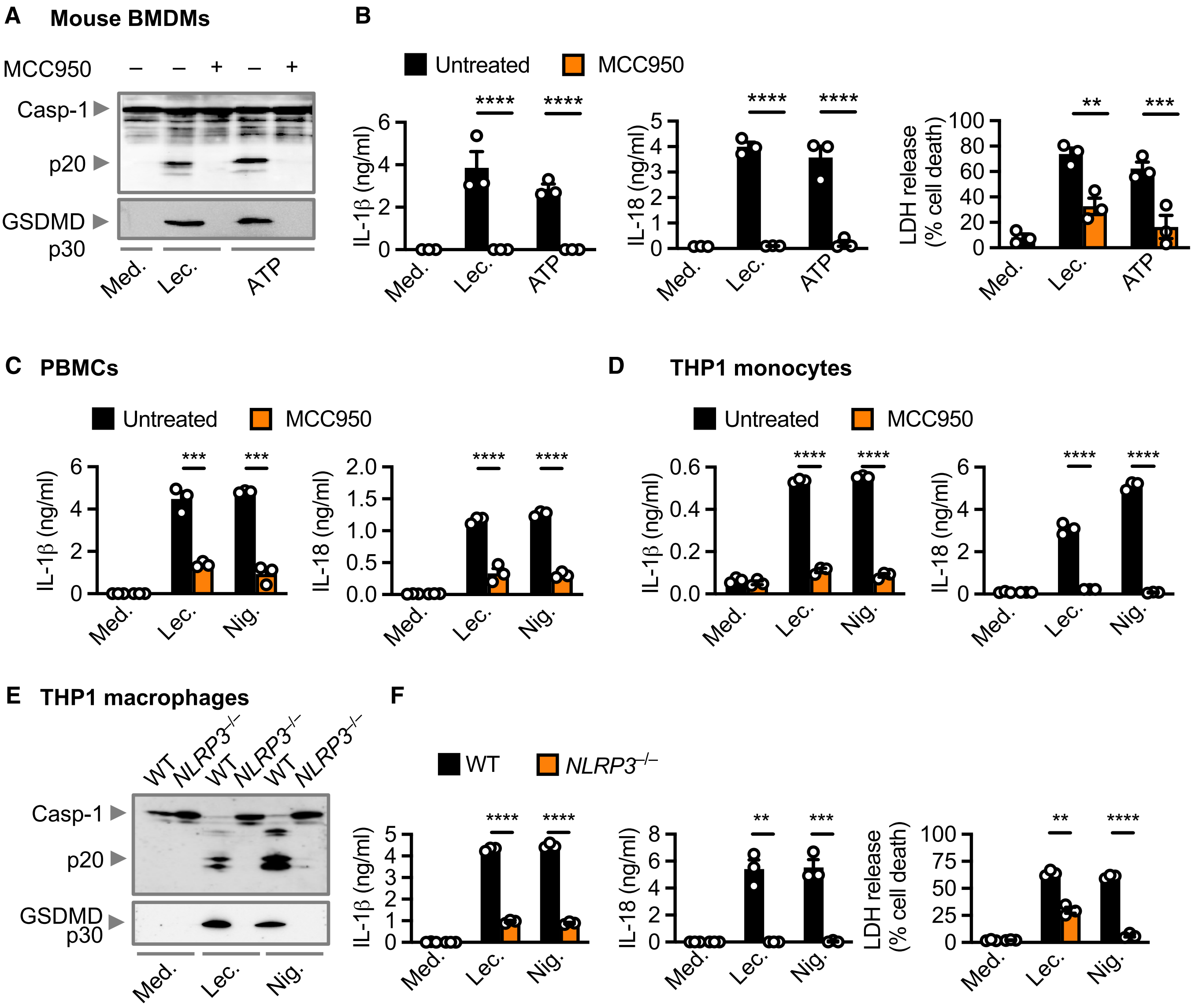

Figure EV3. Lecithinase activates the NLRP3 inflammasome in mouse macrophages, human cell lines and human blood-derived cells.

A Immunoblot analysis of caspase-1 and gasdermin D of WT BMDMs left untreated [Medium alone (Med.)] or LPS-primed and assessed 3 h after stimulation with lecithinase (Lec.) or 1 h after stimulation with ATP in the absence or presence of MCC950 (20 μM).

B Release of IL-1β (left) and IL-18 (middle), and death (right) of BMDMs after treatment as in (A).

C Release of IL-1β (left) and IL-18 (right) of human blood-derived peripheral blood mononuclear cells (PBMCs) from healthy control, left untreated or Pam3CSK4-primed and assessed 3 h after stimulation with lecithinase (Lec.) or 30 min after stimulation with nigericin (Nig.) in the absence or presence of MCC950 (20 μM).

D Release of IL-1β (left) and IL-18 (right) of WT THP-1 monocytes, left untreated or LPS-primed and assessed 3 h after treatment with lecithinase (Lec.) or 30 min nigericin (Nig.) in the absence or presence of MCC950 (20 μM).

E Immunoblot analysis of caspase-1 and gasdermin D of WT or *NLRP3*$^{-/-}$ THP-1 macrophages left untreated or Pam3CSK4-primed and assessed 3 h after stimulation with lecithinase (Lec.) or 30 min after stimulation with nigericin (Nig.).

F Release of IL-1β (left) and IL-18 (middle), and death (right) of THP1 cells as treated in (E).

Data information: Each symbol represents an independent biological replicate (B, C, D and F). NS, not significant, $^{**}P < 0.01$, $^{***}P < 0.001$ and $^{****}P < 0.0001$ (two-tailed *t*-test [B, C, D and F]). Data are representative of three independent biological experiments (A to F; mean and s.e.m. in B, C, D and F).
Source data are available online for this figure.

Figure EV4. Lecithinase-mediated inflammasome activation is independent of lysosomal protease activity.

A Immunoblot analysis of caspase-1 and gasdermin D of WT BMDMs left untreated [Medium alone (Med.)] or LPS-primed and assessed 3 h after stimulation with lecithinase (Lec.) or 30 min after stimulation with nigericin (Nig.) or 5 h after stimulation with LLOMe in the absence or presence of cathepsin B inhibitor (CA-074 Me, 20 μM).

B Release of IL-1β, IL-18, LDH, TNF and KC of WT BMDMs as treated in A.

C Immunoblot analysis of caspase-1 and gasdermin D of WT BMDMs left untreated or LPS-primed and assessed 3 h after stimulation with lecithinase (Lec.) or 30 min after stimulation with nigericin (Nig.) or 5 h after stimulation with LLOMe in the absence or presence of broad spectrum a lysosomal protease inhibitor (E64d, 2 μM).

D Release of IL-1β, IL-18, LDH, TNF and KC of WT BMDMs as treated in C.

Data information: Each symbol represents an independent biological replicate (B and D). NS, not significant. ***P* < 0.01, ****P* < 0.001 and *****P* < 0.0001 (two-tailed *t*-test [B and D]). Data are representative of three independent biological experiments (A–D; mean and s.e.m. in B and D).
Source data are available online for this figure.

 © 2023 The Authors

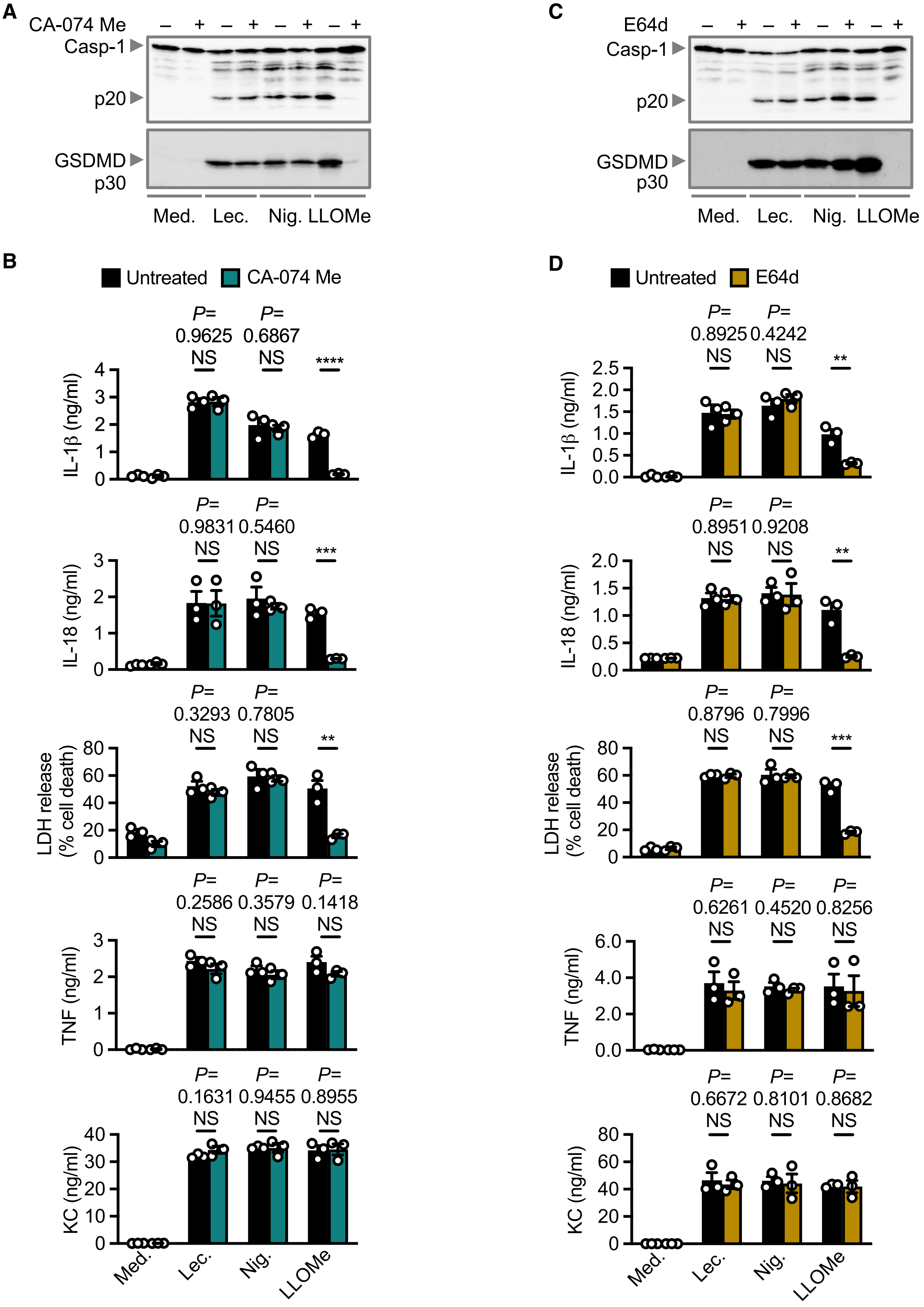
A
CA-074 Me
Casp-1
p20
GSDMD p30
Med. Lec. Nig. LLOMe
C
E64d
Casp-1
p20
GSDMD p30
Med. Lec. Nig. LLOMe
B
Untreated CA-074 Me
P= 0.9625 NS
P= 0.6867 NS
****
IL-1β (ng/ml)
P= 0.9831 NS
P= 0.5460 NS
***
IL-18 (ng/ml)
P= 0.3293 NS
P= 0.7805 NS
**
LDH release (% cell death)
P= 0.2586 NS
P= 0.3579 NS
P= 0.1418 NS
TNF (ng/ml)
P= 0.1631 NS
P= 0.9455 NS
P= 0.8955 NS
KC (ng/ml)
Med. Lec. Nig. LLOMe
D
Untreated E64d
P= 0.8925 NS
P= 0.4242 NS
**
IL-1β (ng/ml)
P= 0.8951 NS
P= 0.9208 NS
**
IL-18 (ng/ml)
P= 0.8796 NS
P= 0.7996 NS
***
LDH release (% cell death)
P= 0.6261 NS
P= 0.4520 NS
P= 0.8256 NS
TNF (ng/ml)
P= 0.6672 NS
P= 0.8101 NS
P= 0.8682 NS
KC (ng/ml)
Med. Lec. Nig. LLOMe

Figure EV4.

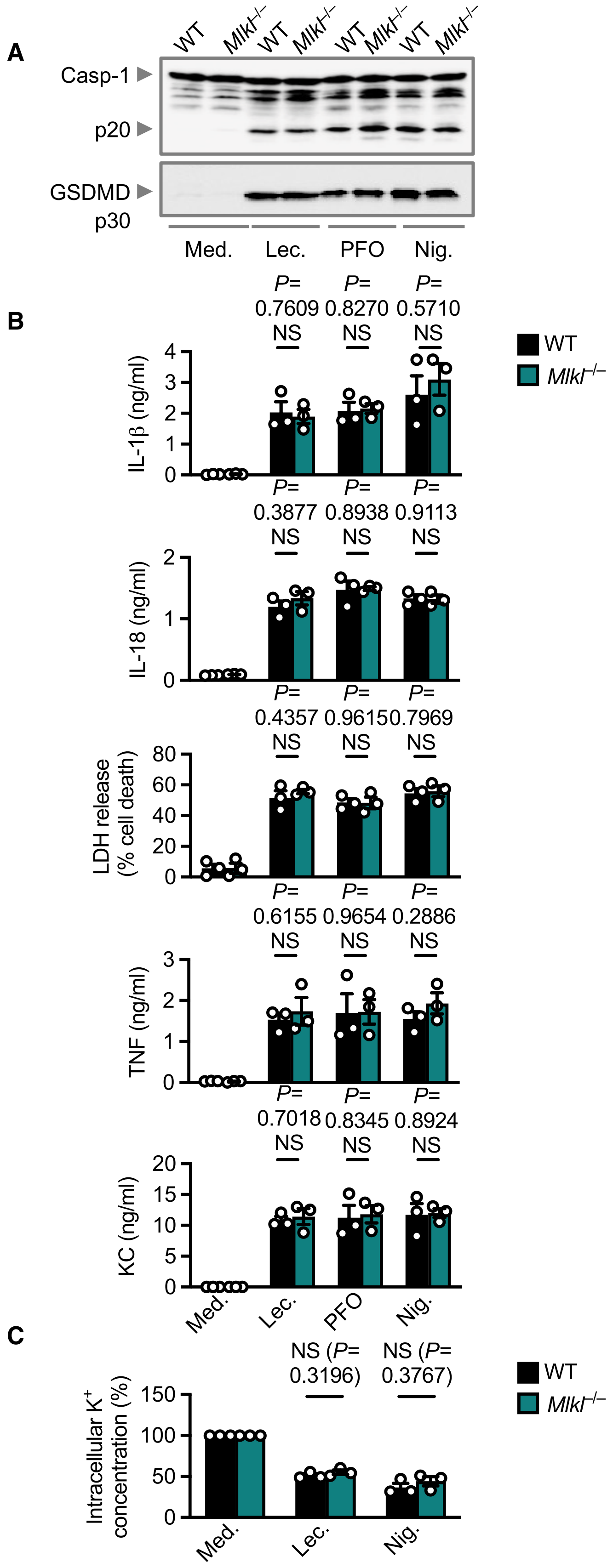

Figure EV5. MLKL is not required for activation of the inflammasome by lecithinase.

A Immunoblot analysis of caspase-1 and gasdermin D in WT or *Mlkl*$^{-/-}$ BMDMs left untreated [Medium alone (Med.)] or LPS-primed and assessed 3 h after stimulation with lecithinase (Lec.) or 3 h after stimulation with perfringolysin O (PFO) or 30 min after stimulation with nigericin (Nig.).

B Release of IL-1β, IL-18, LDH, TNF and KC of WT BMDMs as treated in (A).

C Inductively coupled plasma-optical emission spectrometry analysis of intracellular concentrations of K^+ of BMDMs left untreated or LPS primed and assessed 2 h after stimulation with lecithinase (Lec.), or 30 min after stimulation with nigericin (Nig.).

Data information: Each symbol represents an independent biological replicate (B and C). NS, not significant. (two-tailed *t*-test [B and C]). Data are representative of three independent biological experiments (A–C; mean and s.e.m. in B and C).
Source data are available online for this figure.

 © 2023 The Authors
