# Supplementary material for: Clostridium perfringens virulence factors are nonredundant activators of the NLRP3 inflammasome
Source: EMBO Rep. 2023 Apr 19;24(6):e54600. doi: 10.15252/embr.202254600 (PMC10240202; doi:10.15252/embr.202254600)
Supplement: Supplementary file 4 — PDF+ [file EMBR-24-e54600-s003.pdf]

Article

SOURCE DATA
TRANSPARENT PROCESS
OPEN ACCESS

EMBO reports

Clostridium perfringens virulence factors are nonredundant activators of the NLRP3 inflammasome

Anukriti Mathur[1,†], Callum Kay[1,†], Yansong Xue[1], Abhimanu Pandey[1], Jiwon Lee[2], Weidong Jing[1], Daniel Enosi Tuipulotu[1], Jordan Lo Pilato[1], Shouya Feng[1], Chinh Ngo[1], Anyang Zhao[1], Cheng Shen[1], Melanie Rug[2], Lisa A Miosge[1], Ines I Atmosukarto[3], Jason D Price[3], Sidra A Ali[4], Elizabeth E Gardiner[4], Avril AB Robertson[5], Milena M Awad[6], Dena Lyras[6], Nadeem O Kaakoush[7] & Si Ming Man[1,*]

Abstract

Inflammasome signaling is a central pillar of innate immunity triggering inflammation and cell death in response to microbes and danger signals. Here, we show that two virulence factors from the human bacterial pathogen *Clostridium perfringens* are nonredundant activators of the NLRP3 inflammasome in mice and humans. *C. perfringens* lecithinase (also known as phospholipase C) and *C. perfringens* perfringolysin O induce distinct mechanisms of activation. Lecithinase enters LAMP1$^+$ vesicular structures and induces lysosomal membrane destabilization. Furthermore, lecithinase induces the release of the inflammasome-dependent cytokines IL-1β and IL-18, and the induction of cell death independently of the pore-forming proteins gasdermin D, MLKL and the cell death effector protein ninjurin-1 or NINJ1. We also show that lecithinase triggers inflammation via the NLRP3 inflammasome *in vivo* and that pharmacological blockade of NLRP3 using MCC950 partially prevents lecithinase-induced lethality. Together, these findings reveal that lecithinase activates an alternative pathway to induce inflammation during *C. perfringens* infection and that this mode of action can be similarly exploited for sensing by a single inflammasome.

Keywords cell death; inflammasome; innate immunity; toxins
Subject Categories Immunology; Microbiology, Virology & Host Pathogen Interaction; Signal Transduction
DOI 10.15252/embr.202254600 | Received 4 January 2022 | Revised 18 March 2023 | Accepted 24 March 2023 | Published online 19 April 2023
EMBO Reports (2023) 24: e54600

Introduction

Clostridium perfringens is a human and animal pathogen that causes gastroenteritis and diarrhea associated with food poisoning. In severe cases, disease can progress to necrotizing enterocolitis and sepsis. The most serious clinical manifestation mediated by *C. perfringens* is trauma-associated gas gangrene or clostridial myonecrosis (Darke *et al*, 1977; Stevens *et al*, 2012; Stevens & Bryant, 2018). In untreated cases, clostridial myonecrosis mortality approaches 100% (Leiblein *et al*, 2020). However, even with appropriate management, including surgical debridement of the affected limb and antibiotic treatment, mortality still exceeds 50% when myonecrosis is accompanied by bacteraemia (Martinschek *et al*, 2012; Stevens *et al*, 2012). Many of the deaths are attributable to sepsis and multiorgan failure (McHenry *et al*, 1995).

During *C. perfringens* infection in humans, the pore-forming toxin perfringolysin O (PFO) released by the bacterium is thought to be the primary virulence factor driving diseases such as gas gangrene and enteritis (Verherstraeten *et al*, 2015; Gharieb *et al*, 2021). Indeed, PFO can trigger inflammation via the NLRP3 inflammasome, which, in part, contributes to the progression of gas gangrene (Yamamura *et al*, 2019). However, *C. perfringens* isolates that lack functional PFO can cause inflammation and disease in humans (Myers *et al*, 2006; Uzal *et al*, 2014), suggesting that other virulence factors are critical in the pathogenesis of this pathogen. *Clostridium perfringens* express an ensemble of toxins that may have a role in disease pathogenesis (Uzal *et al*, 2010). Combinations of the four major toxins called α (also known as lecithinase), β, ε and ι, and others such as enterotoxin and β2 toxin, give rise to distinct *C. perfringens*

1 Division of Immunology and Infectious Disease, The John Curtin School of Medical Research, The Australian National University, Canberra, ACT, Australia
2 Centre for Advanced Microscopy, The Australian National University, Canberra, ACT, Australia
3 Lipotek Pty Ltd. The John Curtin School of Medical Research, The Australian National University, Canberra, ACT, Australia
4 Division of Genome Science and Cancer, The Australian National University, Canberra, ACT, Australia
5 School of Chemistry and Molecular Biosciences, The University of Queensland, Brisbane, QLD, Australia
6 Infection and Immunity Program, Monash Biomedicine Discovery Institute and Department of Microbiology, Monash University, Clayton, VIC, Australia
7 School of Medical Sciences, UNSW Sydney, Sydney, NSW, Australia
*Corresponding author. Tel: +612 61256793; E-mail: siming.man@anu.edu.au
†These authors contributed equally to this work as first author

© 2023 The Authors. Published under the terms of the CC BY 4.0 license.

toxinotypes, which have characteristic disease associations. The presence of lecithinase is common to toxinotypes A–G; however, toxinotypes B–G also typically produce additional toxins. For example, toxinotype B produces lecithinase, β-toxin and ε-toxin, whereas toxinotype G produces lecithinase and NetB (Rood *et al*, 2018).

Inflammasomes are innate immune signaling complexes that assemble in the cytoplasm of host cells in response to a variety of pathogens, noxious molecules, and danger signals (Schroder & Tschopp, 2010; He *et al*, 2016; Rathinam & Fitzgerald, 2016). Recognition of these signals, termed pathogen-associated molecular patterns, danger-associated molecular patterns, and homeostasis-altering molecular processes by inflammasome sensors, triggers assembly of an inflammasome signaling hub leading to inflammation and pyroptosis (Man & Kanneganti, 2016; Liston & Masters, 2017). Several pattern-recognition receptors can instigate inflammasome formation, including NLRP1, NLRP3, NLRP6, NLRP9b, NLRP10, NAIP, NLRC4, AIM2, Pyrin, CARD8, and caspase-11 (Xue *et al*, 2019; Pandey *et al*, 2021). Most inflammasome sensors recruit the inflammasome adaptor protein apoptosis-associated speck-like protein containing a caspase activation and recruitment domain (ASC, also known as PYCARD). In turn, ASC can recruit the cysteine protease caspase-1, a process that induces autoproteolytic cleavage and activation of caspase-1. Activated caspase-1 proteolytically cleaves the propyroptotic executioner protein gasdermin D (GSDMD), which induces pores on the plasma cell membrane (He *et al*, 2015; Kayagaki *et al*, 2015; Shi *et al*, 2015). Caspase-1 also proteolytically cleaves the pro-inflammatory cytokines interleukin-1β (IL-1β) and IL-18, which then exit the cell via GSDMD pores. In addition, the membrane disruptor protein called nerve injury-induced protein 1, also known as NINJ1, via an undefined mechanism, oligomerizes to induce plasma membrane rupture and pyroptosis (Kayagaki *et al*, 2021).

Inflammasome sensors can recognize diverse classes of microbes and biomolecules. NLRP3 is the most versatile inflammasome sensor that can respond to a plethora of pathogen-associated molecular patterns, danger-associated molecular patterns, and homeostasis-altering molecular processes, including pore-forming toxins from bacteria (Greaney *et al*, 2015; Hayward *et al*, 2018; Man, 2018; Swanson *et al*, 2019; Jing *et al*, 2021). In this study, we identify the enzymatic toxin lecithinase (also known as alpha toxin or phospholipase C), and PFO as nonredundant toxins secreted by the human bacterial pathogen *C. perfringens* that converge on the activation of the NLRP3 inflammasome. We show that, unlike the pore-forming toxin PFO of *C. perfringens*, lecithinase triggers inflammasome activation via lysosomal membrane destabilization. Our results reveal that functionally distinct toxins from *C. perfringens* are targeted by a single inflammasome to initiate inflammation and cell death in the host. This host strategy may offer a single immune sensor the flexibility to respond to multiple toxins produced by the same bacterium at different stages of infection.

Results

Clostridium perfringens virulence factors are nonredundant activators of the NLRP3 inflammasome

Clostridium perfringens isolates that lack PFO can cause inflammation and disease (Myers *et al*, 2006; Uzal *et al*, 2014), suggesting that other virulence factors of this pathogen are important for disease pathogenesis. To investigate this possibility, we stimulated wild-type (WT) primary bone marrow-derived macrophages (BMDMs) with WT *C. perfringens* and an isogenic mutant strain of *C. perfringens* lacking PFO (called Δ*pfo C. perfringens*). We observed that WT *C. perfringens*, but not Δ*pfo C. perfringens*, induced hallmarks of inflammasome activation within 4 h, including cleavage of caspase-1, secretion of IL-1β and IL-18, and induction of cell death (determined by the release of lactate dehydrogenase [LDH]; Fig EV1A and B), consistent with the observation reported by a previous study (Yamamura *et al*, 2019). Furthermore, WT and Δ*pfo C. perfringens* had an impaired ability to induce any of these responses in *Nlrp3*$^{-/-}$ BMDMs (Fig EV1A and B), indicating a requirement for NLRP3. Indeed, recombinant PFO induced the activation of inflammasome in WT, but not *Nlrp3*$^{-/-}$, BMDMs (Fig EV1C and D). While these observations suggest that PFO is the sole virulence factor inducing activation of the NLRP3 inflammasome, we noticed that overnight stimulation of WT BMDMs with the supernatant of Δ*pfo C. perfringens* led to robust proteolytic cleavage of caspase-1 and GSDMD, secretion of IL-1β and IL-18, and induction of cell death (Fig 1A and B). Overnight stimulation of WT BMDMs with Δ*pfo C. perfringens* bacteria also led to the activation of inflammasome responses (Fig 1A and B). Furthermore, the Δ*pfo C. perfringens* supernatant or bacteria did not induce inflammasome responses in *Nlrp3*$^{-/-}$ BMDMs (Fig 1A and B). These results suggest that virulence factor/s other than PFO can, via NLRP3, trigger activation of these responses.

A candidate toxin which may be involved in the activation of inflammasome responses is the enzymatic toxin lecithinase, encoded by the *cpa* gene, which is ubiquitously present in type A *C. perfringens* strains that infect humans (Uzal *et al*, 2014). In response to stimulation with *C. perfringens* lacking lecithinase (called Δ*cpa C. perfringens*) or their supernatant, we observed cleavage of caspase-1 and GSDMD, secretion of IL-1β and IL-18, and induction of cell death in WT BMDMs (Fig 1A and B). To investigate whether lecithinase and PFO might have overlapping roles in triggering inflammasome activation, we stimulated WT and *Nlrp3*$^{-/-}$ BMDMs with an isogenic mutant strain of *C. perfringens* lacking both toxins (called Δ*pfoA*Δ*cpa*; Awad *et al*, 2001). Importantly, *C. perfringens* lacking both lecithinase and PFO or their supernatant did not induce inflammasome responses in WT and *Nlrp3*$^{-/-}$ BMDMs (Fig 1A and B). These results suggest that lecithinase and PFO are overlapping activators of the NLRP3 inflammasome and that absence of both toxins abrogates *C. perfringens*-induced inflammasome activation. The secretion of tumor necrosis factor (TNF) was similar between WT BMDMs and *Nlrp3*$^{-/-}$ BMDMs stimulated WT and mutant *C. perfringens* strains (Fig 1B), suggesting that the priming of the inflammasome is not affected.

To confirm that lecithinase is a genuine activator of the NLRP3 inflammasome, we stimulated WT BMDMs and BMDMs lacking various inflammasome sensors or components with purified *C. perfringens* lecithinase. Proteolytic cleavage of caspase-1 and GSDMD, secretion of IL-1β, IL-18 and induction of cell death, were impaired in *Nlrp3*$^{-/-}$ and *Asc*$^{-/-}$ and *Casp1/11*$^{-/-}$ BMDMs, whereas these responses were intact in WT, *Nlrc4*$^{-/-}$, *Aim2*$^{-/-}$, *Mefv*$^{-/-}$, and *Casp11*$^{-/-}$ BMDMs (Figs 1C and D, and EV2A and B). Secretion of noninflammasome-dependent cytokines TNF, IL-6 and keratinocyte chemoattractant (KC, also known as CXCL1) was not impaired in

 © 2023 The Authors

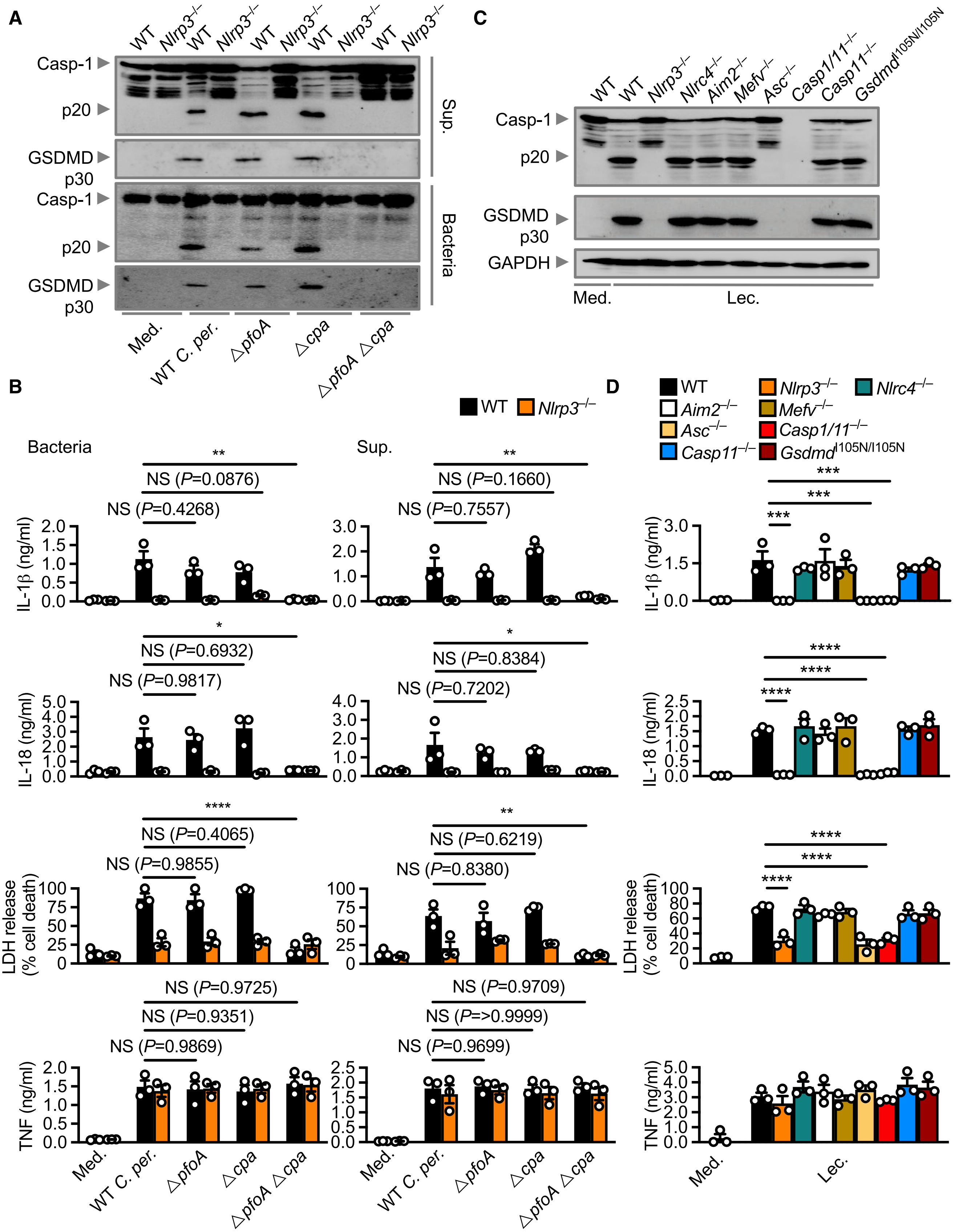
A
WT Nlrp3−/− WT Nlrp3−/− WT Nlrp3−/− WT Nlrp3−/− WT Nlrp3−/−
Casp-1
p20
GSDMD p30
Sup.
Bacteria
Med. WT C. per. △pfoA △cpa △pfoA △cpa
C
WT WT Nlrp3−/− Nlrc4−/− Aim2−/− Mefv−/− Asc−/− Casp1/11−/− Casp11−/− Gsdmd I105N/I105N
Casp-1
p20
GSDMD p30
GAPDH
Med. Lec.
B
WT Nlrp3−/−
Bacteria
Sup.
IL-1β (ng/ml)
IL-18 (ng/ml)
LDH release (% cell death)
TNF (ng/ml)
NS (P=0.0876)
NS (P=0.4268)
NS (P=0.1660)
NS (P=0.7557)
NS (P=0.6932)
NS (P=0.9817)
NS (P=0.8384)
NS (P=0.7202)
NS (P=0.4065)
NS (P=0.9855)
NS (P=0.6219)
NS (P=0.8380)
NS (P=0.9725)
NS (P=0.9351)
NS (P=0.9869)
NS (P=0.9709)
NS (P=>0.9999)
NS (P=0.9699)
Med. WT C. per. △pfoA △cpa △pfoA △cpa
D
WT Nlrp3−/− Nlrc4−/−
Aim2−/− Mefv−/−
Asc−/− Casp1/11−/−
Casp11−/− Gsdmd I105N/I105N
Med. Lec.

Figure 1.

Figure 1. *Clostridium perfringens* secretes lecithinase to activate the NLRP3 inflammasome.

A Immunoblot analysis of caspase-1 (Casp-1) and gasdermin D (GSDMD) in WT or *Nlrp3*$^{-/-}$ unprimed and LPS-primed BMDMs left untreated [Medium alone (Med.)] or assessed 20 h after stimulation with the supernatant (Sup.) of or assessed 20 h after infected with (MOI 100) WT *C. perfringens* (WT *C. per.*), *ΔpfoA C. perfringens* (*ΔpfoA*), *Δcpa C. perfringens* (*Δcpa*, lacking lecithinase) or *ΔpfoAΔcpa C. perfringens* (*ΔpfoAΔcpa*).

B Release of IL-1β (top), IL-18 (middle-top), LDH (middle-bottom) and TNF (bottom) of BMDMs after treatment as in (A).

C Immunoblot analysis of caspase-1, gasdermin D and GAPDH (loading control) in WT or mutant BMDMs left untreated or LPS-primed and assessed 3 h after stimulation with lecithinase (Lec.).

D Release of IL-1β (top), IL-18 LDH (middle-top), (middle-bottom) and TNF (bottom) of BMDMs after treatment as in (C).

Data information: Each symbol represents an independent biological replicate (B and D). NS, not significant, **P* < 0.05, ***P* < 0.01, ****P* < 0.001, and *****P* < 0.0001 (one-way ANOVA with Dunnett's multiple-comparisons test [B and D]). Data are representative of three independent biological experiments (A–D; mean and s.e.m. in B and D).
Source data are available online for this figure.

BMDMs (Figs 1D and EV2C). We confirmed our findings using *C. perfringens* lecithinase from an alternative source and verified that lecithinase induced activation of the NLRP3 inflammasome (Fig EV2D and E). Scanning electron microscopy and transmission electron microscopy techniques revealed that WT BMDMs stimulated with lecithinase exhibited substantial plasma membrane damage, cytoplasmic clearance, and architectural collapse (Appendix Fig S1). Pharmacological inhibition of NLRP3 using the small-molecule inhibitor MCC950 (Coll *et al*, 2015, 2019; Tapia-Abellan *et al*, 2019) impaired the ability of lecithinase to induce inflammasome responses in mouse BMDMs, primary human peripheral blood mononuclear cells (PBMCs), and human THP1 cells (Fig EV3A–D). Furthermore, *NLRP3*$^{-/-}$ THP1 cells stimulated with lecithinase had an impaired ability to undergo inflammasome activation compared with WT THP1 cells (Fig EV3E and F). These findings suggest that lecithinase triggers the activation of the NLRP3 inflammasome and that this process is functionally conserved between mice and humans.

Clostridium perfringens virulence factors activate the NLRP3 inflammasome *in vivo*

Given that lecithinase and PFO activated inflammasome responses in macrophages, we speculated that they might function synergistically to drive inflammasome activation *in vivo*. To assess this possibility, we intraperitoneally infected WT mice with either WT *C. perfringens* or its isogenic mutants, Δ*pfoA*, Δ*cpa*, and Δ*pfoA*Δ*cpa C. perfringens*. We observed elevated levels of serum and peritoneal IL-18 in mice infected with WT *C. perfringens* compared with mice infected with the isogenic mutants Δ*pfoA*, Δ*cpa*, or Δ*pfoA*Δ*cpa* (Fig 2A and B). Furthermore, mice infected with Δ*pfoA*Δ*cpa* had the lowest levels of IL-18 secretion, suggesting that both PFO and lecithinase are required to trigger inflammasome responses. Importantly, WT, *Nlrp3*$^{-/-}$ and *Casp1/11*$^{-/-}$ mice infected with WT *C. perfringens* revealed that *Nlrp3*$^{-/-}$ and *Casp1/11*$^{-/-}$ mice had reduced levels of IL-18 compared with WT mice (Fig 2C and D). These results suggest that *C. perfringens* infection triggers NLRP3 activation *in vivo*. Importantly, levels of TNF and KC were similar in the serum of mice infected with WT, Δ*pfoA*, Δ*cpa*, and Δ*pfoA*Δ*cpa C. perfringens* (Fig EV2F), suggesting that the reduction in IL-18 levels was not due to differences between the different bacterial strains in establishing systemic infection.

Injection of purified lecithinase into WT, *Nlrp3*$^{-/-}$ and *Casp1*$^{-/-}$ mice showed elevated levels of IL-18 in both serum and peritoneal fluid of WT mice compared with *Nlrp3*$^{-/-}$ and *Casp1*$^{-/-}$ mice (Fig 2E and F). In addition, we observed that lecithinase induced lethality in WT mice more rapidly than in *Nlrp3*$^{-/-}$ and *Casp1*$^{-/-}$ mice (Fig 2G). Pharmacological inhibition of NLRP3 using MCC950 reduced the secretion of IL-18 in WT mice and prolonged the

Figure 2. Lecithinase and perfringolysin O synergistically drive inflammation in the host via activation of the NLRP3 inflammasome.

A Concentration of IL-18 in the serum of WT mice, 8 h after intraperitoneal infection with 4 × 10^8 colony-forming units (CFUs) of *C. perfringens* (WT *C. perf.*, *n* = 14), *ΔpfoA C. perfringens* (*ΔpfoA*, *n* = 13), *Δcpa C. perfringens* (*Δcpa*, *n* = 11) or *ΔpfoAΔcpa C. perfringens* (*ΔpfoAΔcpa*, *n* = 12).

B Concentration of IL-18 in the peritoneal fluid of WT mice, 4 h after intraperitoneal infection with 4 × 10^8 CFUs of *C. perfringens* (WT *C. per*, *n* = 14), *ΔpfoA C. perfringens* (*ΔpfoA*, *n* = 15), *Δcpa C. perfringens* (*Δcpa*, *n* = 17) or *ΔpfoAΔcpa C. perfringens* (*ΔpfoAΔcpa*, *n* = 14).

C Concentration of IL-18 in the serum of WT mice (*n* = 10), *Nlrp3*$^{-/-}$ mice (*n* = 9), and *Casp1/11*$^{-/-}$ mice (*n* = 19), 6 h after intraperitoneal infection with 1 × 10^8 CFUs of *C. perfringens*.

D Concentration of IL-18 in the peritoneal fluid of WT mice (*n* = 10), *Nlrp3*$^{-/-}$ mice (*n* = 9), and *Casp1/11*$^{-/-}$ mice (*n* = 19), 3 h after intraperitoneal infection as described in (C).

E Concentration of IL-18 in the serum of WT mice (*n* = 10), *Nlrp3*$^{-/-}$ mice (*n* = 11), and *Casp1*$^{-/-}$ mice (*n* = 11), 4 h after intraperitoneal administration of 0.625 Units/ml of lecithinase (Lec.).

F Concentration of IL-18 in the peritoneal fluid of WT mice (*n* = 10), *Nlrp3*$^{-/-}$ mice (*n* = 11), and *Casp1*$^{-/-}$ mice (*n* = 11), 2 h after intraperitoneal administration of 0.625 Units/ml of lecithinase (Lec.).

G Survival of WT (*n* = 19), *Nlrp3*$^{-/-}$ (*n* = 26) and *Casp1*$^{-/-}$ (*n* = 10) mice, after intraperitoneal administration of 0.312 Units/ml of lecithinase (Lec.).

H Concentration of IL-18 in the serum of WT mice 4 h after intraperitoneal administration with either PBS (*n* = 20) or MCC950 (*n* = 19), followed by intraperitoneal administration of 0.625 Units/ml of lecithinase (Lec.) with a corresponding second dose of either PBS or MCC950.

I Concentration of IL-18 in the peritoneal fluid of WT mice administered with either PBS (*n* = 13) or with MCC950 (*n* = 14), 2 h after intraperitoneal administration of 0.625 Units/ml of lecithinase (Lec.). as in (H).

J Survival of WT mice administered with either PBS (*n* = 20) or MCC950 (*n* = 18), followed by intraperitoneal administration of 0.312 Units/ml of lecithinase (Lec.) as in (H).

Data information: Each symbol represents an individual mouse (A–F, H, and I). ***P* < 0.01, ****P* < 0.001, and *****P* < 0.0001 (two-sided log-rank test [G and J] or one-way ANOVA with Dunnett's multiple-comparisons test [A–F] or two-tailed *t*-test [H and I]). Data are pooled from two independent biological experiments (A–J; mean and s.e.m. in A to D, E, F, H, and I).
Source data are available online for this figure.

 © 2023 The Authors

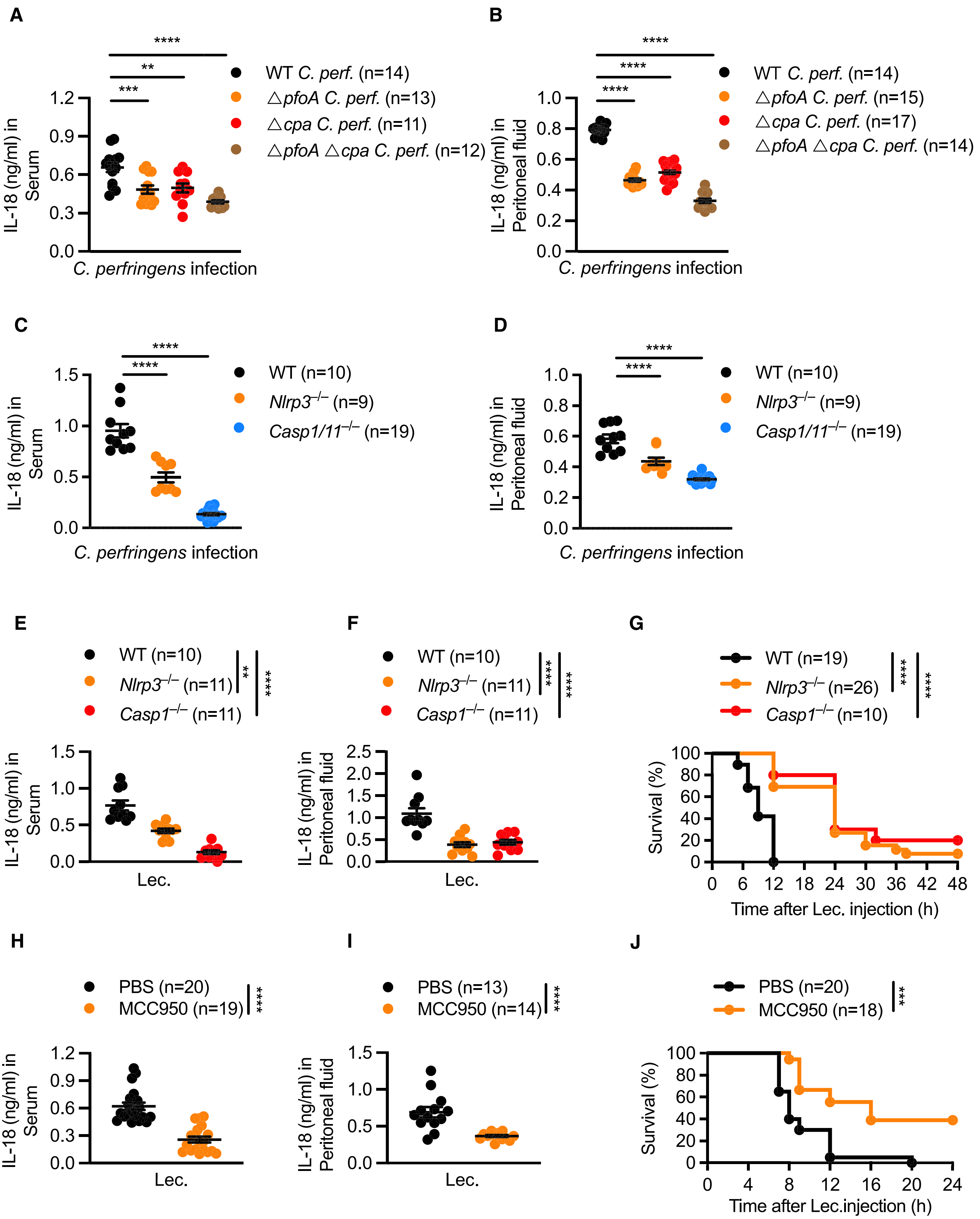
A
IL-18 (ng/ml) in Serum
C. perfringens infection
WT C. perf. (n=14)
△pfoA C. perf. (n=13)
△cpa C. perf. (n=11)
△pfoA △cpa C. perf. (n=12)
B
IL-18 (ng/ml) in Peritoneal fluid
C. perfringens infection
WT C. perf. (n=14)
△pfoA C. perf. (n=15)
△cpa C. perf. (n=17)
△pfoA △cpa C. perf. (n=14)
C
IL-18 (ng/ml) in Serum
C. perfringens infection
WT (n=10)
Nlrp3−/− (n=9)
Casp1/11−/− (n=19)
D
IL-18 (ng/ml) in Peritoneal fluid
C. perfringens infection
WT (n=10)
Nlrp3−/− (n=9)
Casp1/11−/− (n=19)
E
WT (n=10)
Nlrp3−/− (n=11)
Casp1−/− (n=11)
IL-18 (ng/ml) in Serum
Lec.
F
WT (n=10)
Nlrp3−/− (n=11)
Casp1−/− (n=11)
IL-18 (ng/ml) in Peritoneal fluid
Lec.
G
WT (n=19)
Nlrp3−/− (n=26)
Casp1−/− (n=10)
Survival (%)
Time after Lec. injection (h)
H
PBS (n=20)
MCC950 (n=19)
IL-18 (ng/ml) in Serum
Lec.
I
PBS (n=13)
MCC950 (n=14)
IL-18 (ng/ml) in Peritoneal fluid
Lec.
J
PBS (n=20)
MCC950 (n=18)
Survival (%)
Time after Lec.injection (h)

Figure 2.

survival of WT mice in response to lecithinase (Fig 2H–J). These results suggest that the NLRP3 inflammasome contributes to lecithinase-induced inflammation and lethality and that pharmacological blockade of the NLRP3 is potentially protective.

Lecithinase but not perfringolysin O requires phagocytosis to activate the inflammasome

Toxins have different mechanisms of action that may trigger activation of the inflammasome (Greaney *et al*, 2015; Jing *et al*, 2021). Lecithinase ruptured liposomes containing phospholipids and heat inactivation abrogated the phospholipase activity of lecithinase and its ability to activate the NLRP3 inflammasome (Appendix Fig S2A–C). Given its ability to cleave phospholipid moieties, lecithinase may emanate a signal directly from the plasma membrane to activate NLRP3 without requiring cellular entry (Uzal *et al*, 2014). We fluorescently labeled lecithinase with Alexa Fluor 568 (designated as AF568-lecithinase) and stimulated BMDMs with AF568-lecithinase to identify the distribution of lecithinase within the cell. Fluorescent-labeling of lecithinase did not affect its phospholipase activity or ability to induce inflammasome activation (Appendix Fig S2D–F). Confocal microscopy analysis revealed that AF568-lecithinase foci accumulated within BMDMs (Fig 3A and B). Furthermore, cell-fractionation experiments revealed that lecithinase localized to the cytoplasm rather than the plasma membrane (Fig 3C). Depletion of the plasma membrane cholesterol using methyl-β-cyclodextrin (MCD) or inhibition of actin polymerization using cytochalasin D largely prevented the accumulation of AF568-lecithinase in the cytoplasm of BMDMs (Fig 3A and B), suggesting that both membrane cholesterol and actin dynamics may be required for internalization of lecithinase. Inhibition of lecithinase binding to the plasma membrane using MCD abrogated lecithinase-induced inflammasome activation in WT BMDMs, whereas inhibition of phagocytosis using cytochalasin D or latrunculin B partially abrogated inflammasome activation in WT BMDMs. The presence of these inhibitors had no effect on nigericin-mediated inflammasome activation (Fig 3D–I). The secretion of TNF, KC, or IL-6 by BMDMs was not affected by MCD, cytochalasin D or latrunculin B (Appendix Fig S3A–C). MCD also blocked PFO-induced inflammasome activation; however, phagocytosis inhibitors cytochalasin B or cytochalasin D did not impair the ability of PFO to induce inflammasome activation (Fig 3D; Appendix Fig S4A and B). These results suggest that lecithinase, but not PFO, may require phagocytosis and cytosolic access to engage the NLRP3 inflammasome, and highlights that *C. perfringens* produces two functionally distinct toxins that may have different cellular pathways converging on inflammasome activation.

Lecithinase induces lysosomal destabilization

To identify the subcellular localization of lecithinase more precisely, we stimulated BMDMs with AF568-lecithinase followed by analysis with Correlative Light Electron Microscopy (CLEM). We found that AF568-lecithinase largely localized within small- to medium-sized, round-shaped vesicular structures of 0.3–2 μm in diameter (Fig 4A). Further inspection suggests putative sites of lysosomal destabilization (Fig 4A, white arrowheads). Upon internalization, endosomal compartments frequently fuse with lysosomes to enable degradation of pathogens and extracellular molecules (Luzio *et al*, 2007). We labeled the endogenous lysosomal marker LAMP1 in BMDMs and found that AF568-lecithinase puncta localized within LAMP1-positive vesicles (Fig 4B). Three-dimensional reconstruction of AF568-lecithinase revealed a layer of LAMP1 encased lecithinase (Fig 4C). The average distance between the peak signal from the LAMP-1 boundaries (left-side and right-side) and the peak signal from the AF568-lecithinase puncta was 0.6 μm (Fig 4D), consistent with the diameter range of the small-to-medium-sized vesicular structures observed using CLEM. These analyses suggest that lecithinase is found within LAMP1-positive lysosomes.

Crystalline substances, such as silica crystals, can induce lysosomal destabilization and activation of the inflammasome (Hornung *et al*, 2008), but whether enzymatic toxins can engage the lysosomal pathway to trigger inflammasome activation is largely unknown. To further investigate the role of lysosomes in lecithinase-mediated inflammasome activation, we used the dual lysosomal H^+ ATPase and phago-lysosomal fusion inhibitor bafilomycin A (Yamamoto *et al*, 1998; Mauvezin & Neufeld, 2015) and inhibitors of endo-lysosomal fusion, ammonium chloride (Hart & Young, 1991), and

Figure 3. Lecithinase requires cholesterol-dependent phagocytosis and intracellular access to induce inflammasome activation.

A Confocal microscopy analysis of CD11b (green) and lecithinase (red) in unprimed WT BMDMs left untreated [Medium alone (Med.)] or assessed 1 h after stimulation with Alexa Fluor 568 labeled lecithinase (AF568-Lec.) in the absence or presence of cytochalasin D (Cyto D; 50 μM) or methyl-β-cyclodextrin (MCD; 5 mM).
B Quantification of AF568-lecithinase (red) puncta shown in A, showing % of BMDMs positive for red puncta (number of cells counted = 200).
C Immunoblot analysis of His-lecithinase (Lec.) caspase-1 and CD14 of LPS-primed WT BMDMs left untreated or assessed 5 h after treatment with His-tagged lecithinase (His-Lec.). Membrane, membrane fraction; Cytoplasm, cytosolic fraction.
D Immunoblot analysis of caspase-1 and gasdermin D of unprimed or LPS-primed WT BMDMs left untreated or assessed 3 h after stimulation with lecithinase (Lec.), or 3 h after stimulation with perfringolysin O (PFO), or 30 min after stimulation with nigericin (Nig.) in the absence or presence of methyl-β-cyclodextrin (MCD; 5 mM).
E Release of IL-1β (left) and IL-18 (middle), and death (right) of WT BMDMs after treatment as in (D).
F Immunoblot analysis of caspase-1 and gasdermin D of unprimed or LPS-primed WT BMDMs left untreated or assessed 3 h after stimulation with lecithinase (Lec.), or 4 h after infection of with *S.* Typhimurium (*S.* Tm.; MOI, 5), or 30 min after stimulation with nigericin (Nig.) in the absence or presence of cytochalasin D (Cyto D; 50 μM).
G Release of IL-1β (left) and IL-18 (middle), and death (right) of WT BMDMs after treatment as in (F).
H Immunoblot analysis of caspase-1 and gasdermin D of unprimed or LPS-primed WT BMDMs left untreated or assessed 3 h after stimulation with lecithinase (Lec.), or 4 h after infection of with *S.* Typhimurium (*S.* Tm.; MOI, 5), or 30 min after stimulation with nigericin (Nig.) in the absence or presence of latrunculin B (Lat B; 1 μg/ml).
I Release of IL-1β (left) and IL-18 (middle), and death (right) of WT BMDMs after treatment as in (H).

Data information: Scale bar, 12.5 μm (A). Each symbol represents an independent biological replicate (B, E, G, I). NS, not significant. $^{**}P < 0.01$, $^{***}P < 0.001$ and $^{****}P < 0.0001$ (one-way ANOVA with Dunnett's multiple-comparisons test [B] or two-way ANOVAs using Sidak multiple comparison test [E, G, I]). Data are representative of three independent biological experiments (A–I; mean and s.e.m. in B, E, G, I).
Source data are available online for this figure.

 © 2023 The Authors

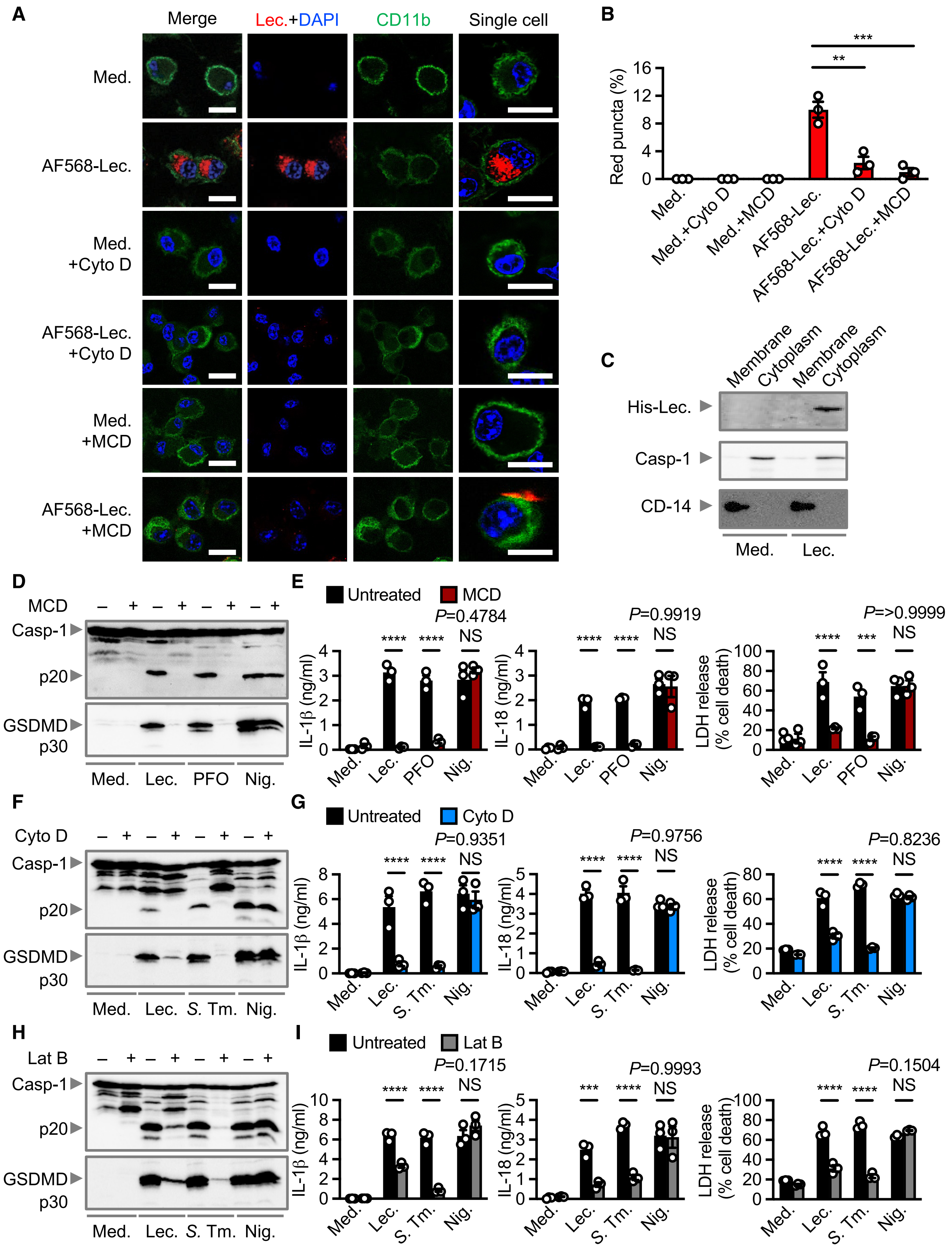
A
Merge
Lec.+DAPI
CD11b
Single cell
Med.
AF568-Lec.
Med. +Cyto D
AF568-Lec. +Cyto D
Med. +MCD
AF568-Lec. +MCD
B
Red puncta (%)
Med.
Med.+Cyto D
Med.+MCD
AF568-Lec.
AF568-Lec.+Cyto D
AF568-Lec.+MCD
C
Membrane
Cytoplasm
Membrane
Cytoplasm
His-Lec.
Casp-1
CD-14
Med.
Lec.
D
MCD
Casp-1
p20
GSDMD p30
Med.
Lec.
PFO
Nig.
E
Untreated
MCD
P=0.4784
P=0.9919
P=>0.9999
IL-1β (ng/ml)
IL-18 (ng/ml)
LDH release (% cell death)
F
Cyto D
G
Cyto D
P=0.9351
P=0.9756
P=0.8236
S. Tm.
H
Lat B
I
Lat B
P=0.1715
P=0.9993
P=0.1504
NS

Figure 3.

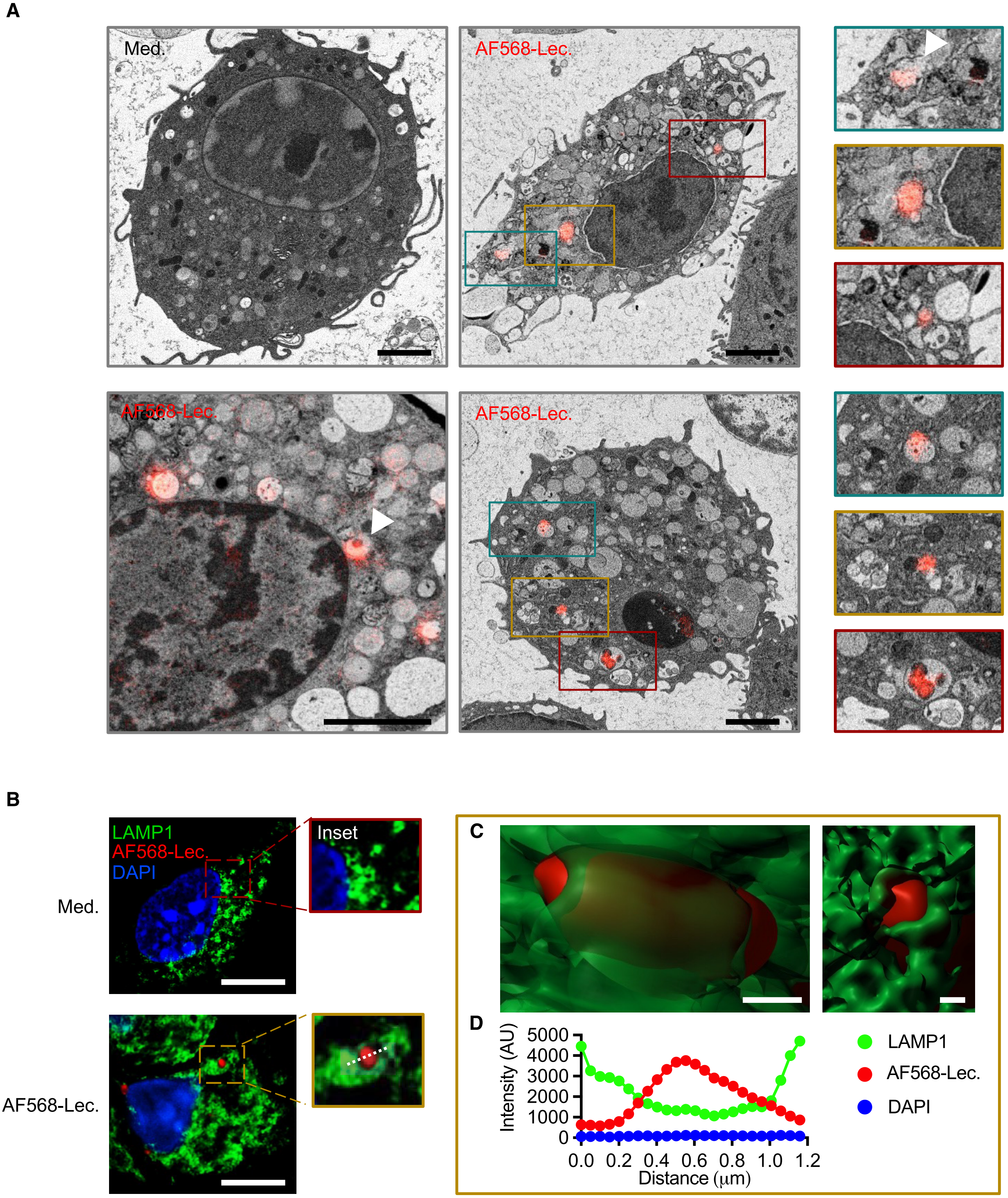

Figure 4.

© 2023 The Authors

◀

Figure 4. Lecithinase is internalized and gains access to the lysosome.

A Correlative light electron microscopy analysis of WT BMDMs left untreated (Medium alone [Med.]) or assessed 1 h after stimulation with Alexa Fluor 568 labeled lecithinase (AF568-Lec.). Arrowheads indicate putative sites of lysosomal destabilization.
B Confocal microscopy analysis of LAMP1 (green) and lecithinase (red) in WT BMDMs left untreated or assessed 1 h after stimulation with AF568-lecithinase.
C Three-dimensional surface rendering of LAMP1 (green) and lecithinase (red) shown in (B).
D Line scan values for LAMP1, AF568-lecithinase, and DAPI taken from the white dashed line in the gold-colored inset in B. These values were plotted and fitted with a single Gaussian equation (Arbitrary Unit [AU]).

Data information: Scale bar, 2 μm (A) 10 μm (B) 0.3 μm (C). Data are representative of two (A and B) independent biological experiments.
Source data are available online for this figure.

concanamycin A (Huss *et al*, 2002). We found that bafilomycin A blocked inflammasome activation in WT BMDMs in response to lecithinase, whereas ammonium chloride and concanamycin A partially inhibited inflammasome activation in WT BMDMs stimulated with lecithinase (Fig 5A–D; Appendix Fig S5A and B). In contrast, nigericin-induced inflammasome activation was not affected by bafilomycin A, ammonium chloride or concanamycin A (Fig 5A–D; Appendix Fig S5A and B). Neither bafilomycin A, ammonium chloride or concanamycin A affected the secretion of TNF, KC and/or IL-6 in BMDMs (Appendix Figs S5B and, S6A and B), suggesting that priming is not impaired.

To assess whether lecithinase induced lysosomal destabilization, WT BMDMs were loaded with acridine orange, a lysotrophic dye which preferentially accumulates in the acidic environment within the lysosome (Pierzynska-Mach *et al*, 2014). A loss of lysosomal stability leads to the release of acridine orange into the cytoplasm, and therefore, a loss in the fluorescence intensity of the dye, which can be quantified by flow cytometry (Wang *et al*, 2018). We observed a loss of fluorescence intensity of acridine orange in BMDMs stimulated with lecithinase (Fig 5E; Appendix Fig S7A), a level of loss fluorescence intensity similar to that observed in BMDMs stimulated with the lysosomal destabilization agent and activator of the NLRP3 inflammasome, LLOMe (Fig 5E; Hornung *et al*, 2008). In addition, we observed a similar level of lecithinase-induced loss of acridine orange fluorescence intensity between WT and *Nlrp3*$^{-/-}$ BMDMs (Fig 5E), suggesting that lysosomal destabilization occurs prior to the activation of NLRP3. Further, MCD, cytochalasin D, bafilomycin A or ammonium chloride partially prevented a loss of fluorescence intensity of acridine orange in BMDMs stimulated with lecithinase (Fig 5F), suggesting that internalization and lysosomal localization of lecithinase may be upstream of lysosomal membrane destabilization. Further, heat-inactivated lecithinase did not induce a substantial loss of acridine orange in BMDMs, and did not induce inflammasome activation (Fig 5F; Appendix Fig S2B and C).

Lysosomal destabilization can precede or succeed cell death (Lima *et al*, 2013; Frank & Vince, 2019). To evaluate the kinetics between lysosomal destabilization and induction of cell death, we designed a flow cytometric system to track lysosomal destabilization and cell death in BMDMs by quantifying the amount of lysosome-associated acridine orange leakage and accumulation of the live-dead dye DAPI in the nucleus, respectively (Fig 5G; Appendix Fig S7B). This flow cytometry system is defined by an acridine orange signal on the y-axis indicating intact or ruptured lysosomes, and the DAPI signal on the x-axis indicating live or dead cells, resulting in four quadrants (Q1: Live cells with intact lysosomes; Q2: Dead cells with intact lysosomes; Q3: Dead cells with ruptured lysosomes; Q4: Live cells with ruptured lysosomes; Appendix Fig S7B). BMDMs undergoing lysosomal destabilization followed by cell death over time will traverse in the order of Q1 → Q4 → Q3 (Fig 5G). In contrast, BMDMs undergoing cell death followed by

Figure 5. Endo-lysosomal transport and lysosomal destabilization is required for NLRP3 activation by lecithinase. ▶

A Immunoblot analysis of caspase-1 and gasdermin D of unprimed or LPS-primed WT BMDMs left untreated (Medium alone [Med.]) or assessed 3 h after stimulation with lecithinase (Lec.), or 30 min after stimulation with nigericin (Nig.) in the absence or presence of bafilomycin A (Baf A; 100 nM).
B Release of IL-1β (left) and IL-18 (middle), and death (right) of WT BMDMs after treatment as in (A).
C Immunoblot analysis of caspase-1 and gasdermin D of unprimed or LPS-primed WT BMDMs left untreated or assessed 3 h after stimulation with lecithinase (Lec.), or 16 h after infection with *F. novicida* (*F. novi.*, MOI 100), or 30 min after stimulation with nigericin (Nig.) in the absence or presence of ammonium chloride (NH_4Cl; 20 μM).
D Release of IL-1β (left) and IL-18 (middle), and death (right) of WT BMDMs after treatment as in (C).
E Flow cytometry analysis of WT or *Nlrp3*$^{-/-}$ BMDMs stained with acridine orange (1 μg/ml) and assessed 30 min after stimulation with lecithinase (Lec.) or 30 min after stimulation with LLOMe.
F Flow cytometry analysis of WT BMDMs stained with acridine orange (1 μg/ml) and pre-treated with various inhibitors (MCD, Cytochalasin D, Bafilomycin A, ammonium chloride) for 30 min, and assessed 60 min after stimulation with lecithinase (Lec.). WT BMDMs stained with acridine orange were also assessed 60 min after stimulation with heat-treated lecithinase (Heat).
G Schematics of the flow cytometry system used to determine the kinetics of lysosomal destabilization and cell death. An activator inducing lysosomal destabilization followed by cell death will result in a cell population transitioning from quadrants Q1 → Q4 → Q3 (teal arrow). An activator inducing cell death followed by lysosomal destabilization will result in a cell population transitioning from quadrants Q1 → Q2 → Q3 (gold arrow).
H Flow cytometry analysis of WT BMDMs stained with acridine orange (1 μg/ml) and DAPI (0.5 μg/ml) and assessed over time after stimulation with lecithinase (Lec.).
I Flow cytometry analysis of WT BMDMs stained with acridine orange (1 μg/ml) and DAPI (0.5 μg/ml) and assessed over time after stimulation with LLOMe.
J Flow cytometry analysis of WT BMDMs stained with acridine orange (1 μg/ml) and DAPI (0.5 μg/ml) and assessed over time after LPS transfection.

Data information: Each symbol represents an independent biological replicate (B, D, E, F, H, I, J). NS, not significant. **P* < 0.05, ***P* < 0.01, ****P* < 0.001 and *****P* < 0.0001 (two-way ANOVAs using Sidak multiple comparison test [B, D, E and F]). Data are representative of three independent biological experiments (A–F, H–J; mean and s.e.m. in B, D, E, F, H, I, J).
Source data are available online for this figure.

© 2023 The Authors

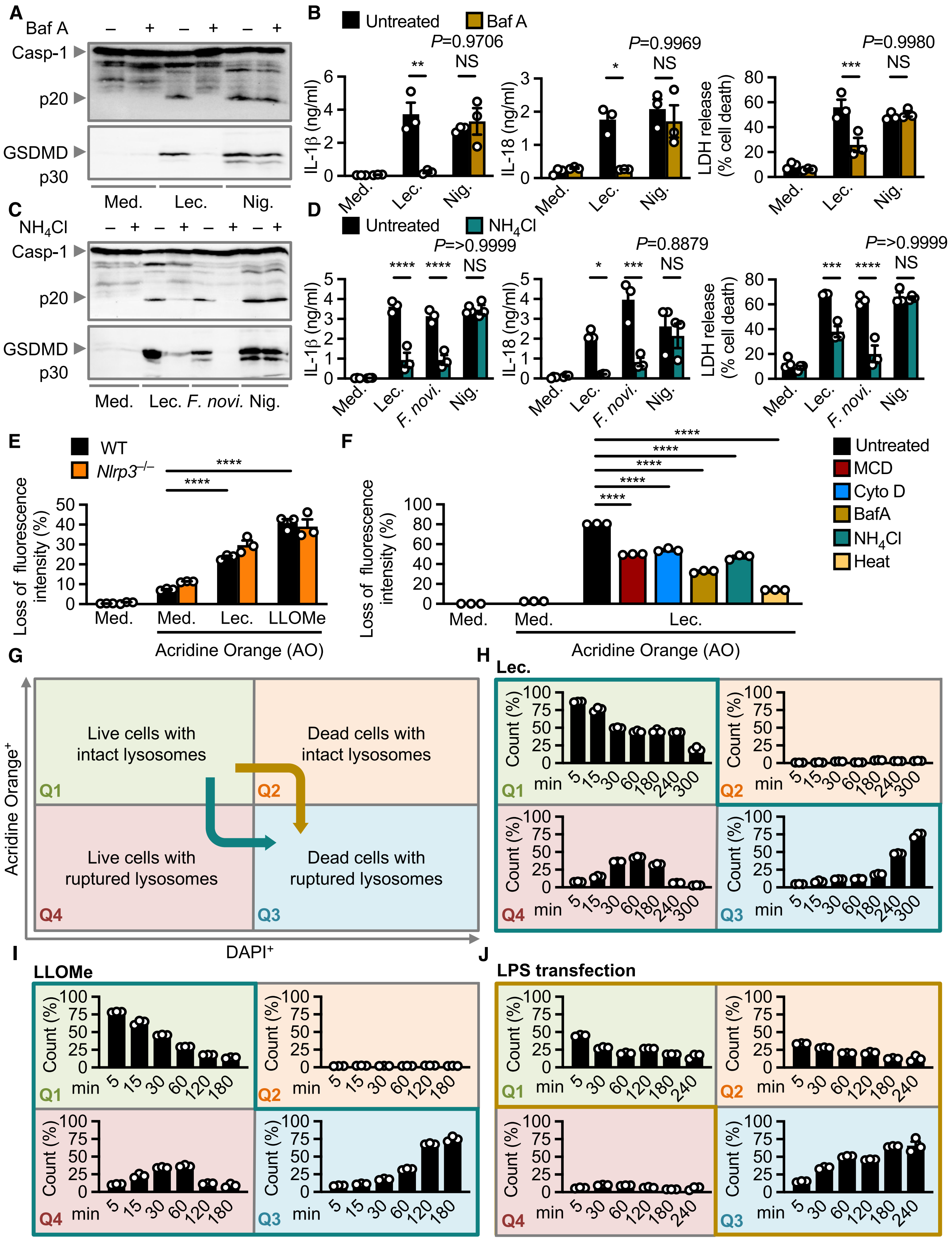

Figure 5.

© 2023 The Authors

lysosomal destabilization over time will traverse in the order of Q1 → Q2 → Q3 (Fig 5G).

Using this analysis, we observed a time-dependent transition of lecithinase-stimulated BMDMs from Q1 (Live cells with intact lysosomes) to Q4 (Live cells with ruptured lysosomes), eventually accumulating in Q3 (Dead cells with ruptured lysosomes; Fig 5H; Appendix Fig S7C). We observed a similar pattern of transition over time for BMDMs stimulated with LLOMe (Fig 5I; Appendix Fig S7C). These findings suggest that lecithinase triggers lysosomal destabilization before the activation of cell death. In contrast, following transfection of LPS, BMDMs transitioned from Q1 (Live cells with intact lysosomes) to Q2 (Dead cells with intact lysosomes), and ultimately accumulated in Q3 (Dead cells with ruptured lysosomes; Fig 5J; Appendix Fig S7C), suggesting that LPS-induced cell death occurs without lysosomal destabilization, but lysosomes eventually disintegrate within the cellular corpses. Together, these data not only suggest that lecithinase localizes to and ruptures lysosomes, but position lecithinase-induced lysosomal destabilization firmly upstream of inflammasome activation and cell death.

Lecithinase-induced NLRP3 inflammasome activation requires K^+ efflux prior to and after lysosomal destabilization, but not lysosomal cathepsin release

Unlike several inflammasome complexes which have a more well-defined mechanism of activation, such as AIM2 or NAIP-NLRC4, a unified mechanism for the activation of NLRP3 has not been fully established (Mathur *et al*, 2018). Lysosomal cathepsins are released following lysosomal destabilization (Campden & Zhang, 2019). To assess the role of cathepsins in lecithinase-mediated NLRP3 activation following lysosomal destabilization, we pretreated WT BMDMs with a cell-permeable cathepsin B inhibitor, CA-074 Me and a pan-cathepsin inhibitor, E64d, followed by stimulation with lecithinase, nigericin or LLOMe. We observed that lysosomal cathepsin activity was not required for lecithinase-induced or nigericin-induced inflammasome activation (Fig EV4A–D). These findings suggest that lysosomal cathepsins may not be a universal requirement for NLRP3 activators that act via destabilization of the endo-lysosomal pathway.

Another proposed mechanism of NLRP3 activation is via K^+ efflux following plasma membrane disruption (Petrilli *et al*, 2007; Munoz-Planillo *et al*, 2013; Ruhl & Broz, 2015). Therefore, it is possible that plasma membrane damage might lead to K^+ efflux-induced lysosomal destabilization or that lysosomal destabilization might lead to K^+ efflux. To determine whether lecithinase-mediated inflammasome activation required K^+ efflux, BMDMs were stimulated with lecithinase, followed by quantification of intracellular K^+ concentrations using inductively coupled plasma optical emission spectrometry (ICP-OES). Compared with untreated BMDMs, the intracellular K^+ concentration reduced substantially in BMDMs stimulated with lecithinase, similar to that observed in BMDMs stimulated with ATP (Appendix Fig S8A). Furthermore, supplementation of extracellular KCl in the cell culture media inhibited, in a dose-dependent manner, lecithinase-induced inflammasome activation (Appendix Fig S8B and C). Treatment of BMDMs with MCD prevented lecithinase-induced K^+ efflux, whereas cytochalasin D and bafilomycin A partially prevented lecithinase-induced K^+ efflux (Appendix Fig S8D). None of these treatments impaired nigericin-induced loss of intracellular K^+ concentration (Appendix Fig S8D). These results suggest that cholesterol-mediated attachment of lecithinase to the plasma membrane is an apical step leading to lecithinase-induced K^+ efflux, whereas phagocytosis and lysosomal destabilization partially contribute to the efflux of K^+. Therefore, whilst some efflux of K^+ occurs following binding of lecithinase to the plasma membrane, it is not sufficient to trigger NLRP3 activation. It is plausible that more substantial levels of K^+ efflux occur following internalization of lecithinase and lysosomal membrane destabilization, leading to the activation of the NLRP3 inflammasome.

Lecithinase-mediated plasma membrane rupture occurs independently of GSDMD, MLKL, NINJ1 and caspase-8

Following formation of the inflammasome, caspase-1 or caspase-11 mediates the proteolytic cleavage of GSDMD, liberating the N-terminal domain of GSDMD to oligomerize in the plasma membrane to form membrane pores (He *et al*, 2015; Kayagaki *et al*, 2015; Shi *et al*, 2015). In response to lecithinase, WT and *Gsdmd*$^{I105N/I105N}$ BMDMs (encoding a mutant GSDMD protein lacking the ability to form pores; Kayagaki *et al*, 2015; Aglietti *et al*, 2016; Sborgi *et al*, 2016) released similar levels of IL-1β, IL-18, and LDH and underwent cleavage of caspase-1 and GSDMD (Fig 6A and B). Similar levels of IL-1β and IL-18 were released by WT and *Gsdmd*$^{I105N/I105N}$ BMDMs, consistent with previous findings in response to certain stimuli, such as MSU crystals (Rashidi *et al*, 2019). We performed additional time-course experiments using cells which lacked GSDMD. We found that the release of IL-1β, IL-18 and LDH between WT and *Gsdmd*$^{-/-}$ BMDMs were again similar (Fig 6C and D). Kinetic analysis of cell death using Incucyte assays also revealed no difference between WT, *Gsdmd*$^{I105N/I105N}$ and *Gsdmd*$^{-/-}$ BMDMs treated with lecithinase (Fig 6E–H), confirming that GSDMD is not required for lecithinase-mediated release of IL-1β and IL-18 and cell death. Unlike lecithinase, transfection of LPS induced GSDMD-dependent responses (Fig 6A–D, F, and H), validating the functional role of GSDMD in our assays.

The pseudokinase mixed-lineage kinase domain-like (MLKL) is a functionally similar protein to GSDMD that mediates the final stages of necroptosis (Murphy, 2020). Upon phosphorylation by RIPK3, MLKL oligomerizes in the plasma membrane and drives necroptotic cell death (Sun *et al*, 2012; Murphy *et al*, 2013). We observed similar levels of inflammasome activation, secretion of IL-1β, IL-18, TNF, and KC, and cell death between WT and *Mlkl*$^{-/-}$ BMDMs treated with lecithinase, PFO and nigericin (Fig EV5A and B). Intracellular K^+ levels were also similar between WT and *Mlkl*$^{-/-}$ BMDMs treated with lecithinase (Fig EV5C), suggesting that MLKL is not involved in the conduit of K^+ ions. Therefore, lecithinase-induced cell death does not rely on GSDMD or MLKL pores in the plasma membrane.

Following inflammasome activation, larger cytoplasmic proteins such as LDH and HMGB1 cannot escape through the narrower GSDMD pores (Broz *et al*, 2020). These host-derived danger signals are instead released following physical rupture of the plasma membrane, a newly characterized programmed process dependent on the oligomerization of the membrane-associated protein NINJ1 on the plasma membrane (Kayagaki *et al*, 2021). As expected, caspase-1 and GSDMD activation and the secretion of IL-1β and IL-18 were

© 2023 The Authors

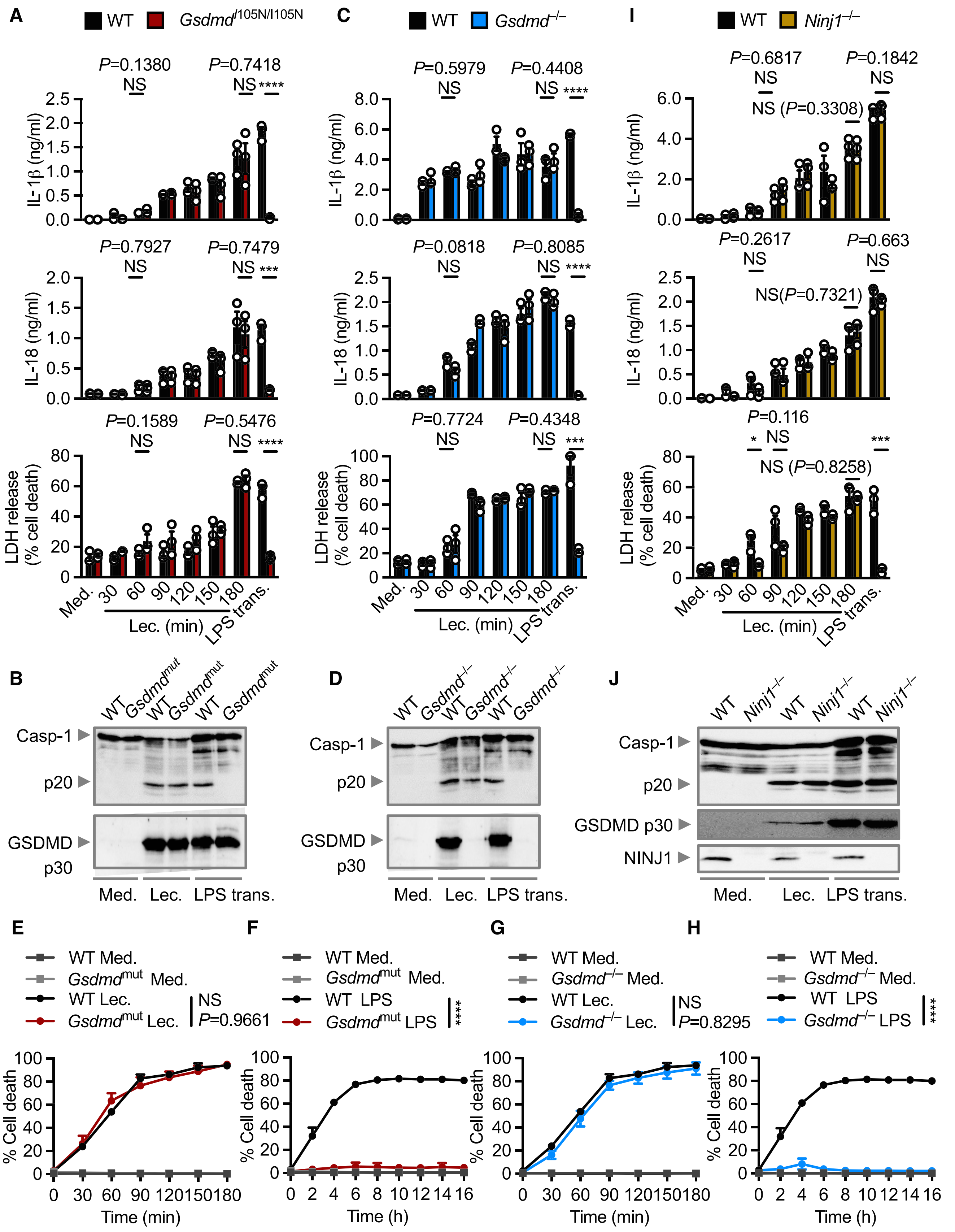

Figure 6.

© 2023 The Authors

Figure 6. GSDMD is not required for lecithinase-mediated cytokine release and cell death but NINJ1 is partially required for cell death.

A Release of IL-1β (top) and IL-18 (middle), and death (bottom) of WT or *Gsdmd*$^{I105N/I105N}$ BMDMs left untreated [Medium alone (Med.)] or LPS-primed and assessed for a range of time points after stimulation with lecithinase (Lec.) or 16 h after transfection with LPS (LPS trans.).

B Immunoblot analysis of caspase-1 and gasdermin D of WT or *Gsdmd*$^{I105N/I105N}$ BMDMs left untreated or LPS-primed and assessed 3 h after stimulation with lecithinase (Lec.) or 16 h after transfection with LPS (LPS trans.).

C Release of IL-1β (top) and IL-18 (middle), and death (bottom) of WT or *Gsdmd*$^{-/-}$ BMDMs left untreated or LPS-primed and assessed for a range of time points after stimulation with lecithinase (Lec.) or 16 h after transfection with LPS (LPS trans.).

D Immunoblot analysis of caspase-1 and gasdermin D of WT or *Gsdmd*$^{-/-}$ BMDMs left untreated or LPS-primed and assessed 3 h after stimulation with lecithinase (Lec.) or 16 h after transfection with LPS (LPS trans.).

E, F IncuCyte live-imaging analysis of the viability of BMDMs after treatment as in A.

G, H IncuCyte live-imaging analysis of the viability of BMDMs after treatment as in C.

I Release of IL-1β (top) and IL-18 (middle), and death (bottom) of WT or *Ninj1*$^{-/-}$ BMDMs left untreated or LPS-primed and assessed for a range of time points after stimulation with lecithinase (Lec.) or 16 h after transfection with LPS (LPS trans.).

J Immunoblot analysis of caspase-1, gasdermin D and NINJ1 of WT or *Ninj1*$^{-/-}$ BMDMs left untreated or LPS-primed and assessed 3 h after stimulation with lecithinase (Lec.) or 16 h after transfection with LPS (LPS trans.).

Data information: Each symbol represents an independent biological replicate (A, C and I). NS, not significant, *$P < 0.05$, ***$P < 0.001$ and ****$P < 0.0001$ (two-tailed *t*-test [A, C, E–I]). Data are representative of three independent biological experiments (A–J; mean and s.e.m. in A, C, E–I).
Source data are available online for this figure.

largely similar between WT and *Ninj1*$^{-/-}$ BMDMs in response to stimulation with lecithinase or transfection of LPS (Fig 6I and J). The release of LDH in *Ninj1*$^{-/-}$ BMDMs transfected with LPS was substantially impaired (Fig 6I). In response to lecithinase stimulation, we observed a very partial reduction in the release of LDH in *Ninj1*$^{-/-}$ BMDMs between 60 and 90 min of stimulation compared with WT BMDMs; however, there was no difference across other time points (Fig 6I). These data collectively suggest lecithinase-mediated plasma membrane destabilization is largely independent of NINJ1.

Finally, whilst we have identified a role for caspase-1 in lecithinase-induced plasma membrane destabilization, it is possible that other caspases may play a role. In particular, caspase-8 has been implicated in NLRP3 activation in response to certain stimuli (Allam *et al*, 2014; Gurung *et al*, 2014; Vince *et al*, 2018). We observed that pretreatment of WT BMDMs with the pan-caspase inhibitor QVD, but not the caspase-8-specific inhibitor Z-ietd, inhibited the secretion of IL-1β and IL-18 and cell death in response to lecithinase (Appendix Fig S9). Neither inhibitor blocked the secretion of TNF. Cell death induced by staurosporine, an inducer of apoptosis, was blocked by both QVD and Z-ietd. These data suggest that caspase-8 is not required for lecithinase-mediated NLRP3 activation.

Discussion

Pathogen-associated molecular patterns are sensed by pattern-recognition receptors, leading to activation of an immune response. Activation of inflammasome sensor proteins is a central mechanism driving host immunity. Here, we identified lecithinase produced by the human and animal bacterial pathogen *C. perfringens* as an activator of the NLRP3 inflammasome. Our study extends the concept that a single inflammasome sensor exploits immune sensing of functionally distinct toxins produced by the same pathogen, in this case, lecithinase and PFO of *C. perfringens*. A previous study has shown a role for PFO, but not lecithinase, in NLRP3 activation in BMDMs following 3 h of infection with *C. perfringens* (Yamamura *et al*, 2019). In our study, we initially stimulated WT BMDMs with *C. perfringens* for 4 h and identified a role for PFO but not lecithinase at this timepoint. However, overnight stimulation identified roles for both PFO and lecithinase in NLRP3 inflammasome activation. The differential kinetics between PFO and lecithinase in activating NLRP3 may be due to delayed or reduced production of lecithinase relative to PFO during the early phase of the infection, which is supported by studies showing that the JIR325 strain, a toxinotype A *C. perfringens*, used in our study has relatively low lecithinase production (Bullifent *et al*, 1996; Keyburn *et al*, 2006). Therefore, it is possible that *C. perfringens* from other toxinotypes may activate the inflammasome differently and would require further investigation.

In addition to *C. perfringens*, the ability of NLRP3 to sense multiple virulence factors from the same pathogen, such as *Bacillus cereus* and *S. aureus* (Munoz-Planillo *et al*, 2009; Enosi Tuipulotu *et al*, 2021), highlights the versatile nature of inflammasome sensors. In the case of *B. cereus*, HBL and NHE are both pore-forming toxins sensed by NLRP3 (Mathur *et al*, 2019; Fox *et al*, 2020), whereas in the case of *S. aureus*, a combination of hemolysins and leucocidins are sensed by NLRP3 (Munoz-Planillo *et al*, 2009; Perret *et al*, 2012; Wang *et al*, 2020). Whilst these two pathogens produce functionally similar toxins that are sensed by a similar mechanism by NLRP3, our work demonstrated that NLRP3 can also detect functionally distinct toxins which act via disparate mechanisms.

The majority of bacterial toxins that activate the NLRP3 inflammasome are pore-forming toxins; however, several enzymatic toxins which do not induce pore-formation, such as *Mycoplasma pneumoniae* CARDS toxin and Shiga toxin, have been reported to activate the NLRP3 inflammasome (Bose *et al*, 2014; Lee *et al*, 2016). Unlike pore-forming toxins that emanate a signal from the plasma membrane to trigger K^+ efflux and subsequent NLRP3 activation (Jing *et al*, 2022), lecithinase accesses the lysosome to trigger NLRP3 activation. Several exogenous activators of NLRP3, such as asbestos and silica crystals (Dostert *et al*, 2008; Hornung *et al*, 2008), and endogenous aggregates, including circulating ASC specks, neuronal amyloid-β fibrils and multiple myeloma-associated β_2-microglobulin fibrils (Halle *et al*, 2008; Franklin *et al*, 2014; Hofbauer *et al*, 2021), have been described to undergo a pathway of internalization, endo-lysosomal trafficking and lysosomal membrane destabilization. However, unlike many of these exogenous and endogenous activators, lecithinase-induced activation of the inflammasome does not appear to require cathepsin activities, similar to that in *Francisella*

© 2023 The Authors

novicida infection (Qi *et al*, 2016), indicating that lysosomal cathepsins are not universally required for inflammasome activation triggered by destabilization of lysosomal pathways.

Lysosomal destabilization can occur prior to K^+ efflux across the plasma membrane (Munoz-Planillo *et al*, 2013); however, it remains unclear precisely how lysosomal destabilization facilitates K^+ efflux and activation of NLRP3. One possibility is that the activator itself can escape the disrupted lysosome and damage the plasma membrane from the cytoplasmic side, increasing the permeability of the plasma membrane to K^+. It is also possible that the lysosomal destabilization releases a pro-pyroptotic factor from within the lysosome and causes plasma membrane damage in the cytoplasm. These scenarios may also offer an explanation as to why GSDMD is not required for cell death triggered by lecithinase and other phagocytosed NLRP3 activators, such as platelet activating factor and silica crystals (Deng *et al*, 2019; Rashidi *et al*, 2019). Future work investigating the link between the lysosomes, K^+ efflux and NLRP3 activation is required, and will deepen our understanding of the pathways that lead to activation of the NLRP3 inflammasome. Indeed, other studies have identified a role for the lysosome-tethered Ragulator-Rag complex in GSDMD oligomerization and pyroptosis (Evavold *et al*, 2021; Zheng *et al*, 2021; Tsujimoto *et al*, 2022), highlighting the importance of the lysosome in inflammasome-driven cell death.

Whilst NINJ1 was recently identified as a terminal host component of mammlian cells required for lytic cell death (Kayagaki *et al*, 2021), many questions surrounding its activation mechanism and physiological role remain unanswered. This initial study found a considerable reduction in cell death and LDH release in *Ninj1*$^{-/-}$ BMDMs compared with WT BMDMs in response to stimulation with *S*. Typhimurium and *Citrobacter rodentium* (Kayagaki *et al*, 2021). They also found that NINJ1 deficiency was protective in mice infected with *C. rodentium* (Kayagaki *et al*, 2021). Given that we found minimal difference in cell death between WT and *Ninj1*$^{-/-}$ BMDMs in response to lecithinase, the cellular and physiological roles of NINJ1 may be more subtle for certain cell-death inducers.

In conclusion, we identified that functionally and mechanistically dissimilar toxins of *C. perfringens* are exploited for innate immune sensing by an inflammasome sensor protein. Further studies investigating the interplay between lecithinase and PFO or therapeutic blockade of NLRP3 would provide more treatment options for patients with *C. perfringens* infection.

Materials and Methods

Mice

C57BL/6, *Gsdmd*$^{I105N/I105N}$ (Kayagaki *et al*, 2015), *Ninj1*$^{-/-}$ (Kayagaki *et al*, 2021) and *Mefv*$^{-/-}$ (Feng *et al*, 2022) mice were sourced from The Australian National University. *Nlrp3*$^{-/-}$ (Kovarova *et al*, 2012), *Casp1*$^{-/-}$ (Rauch *et al*, 2017), *Casp11*$^{-/-}$ (Wang *et al*, 1998) and *Casp1/11*$^{-/-}$ (Kuida *et al*, 1995) mice were sourced from The Jackson Laboratory. *Gsdmd*$^{-/-}$ (Kayagaki *et al*, 2015), *Nlrc4*$^{-/-}$ (Mariathasan *et al*, 2004) and *Asc*$^{-/-}$ (Mariathasan *et al*, 2004) mice were sourced from the University of Queensland. *Aim2*$^{-/-}$ mice (Jones *et al*, 2010) were sourced from Genentech. *Mlkl*$^{-/-}$ (Murphy *et al*, 2013) mice were sourced from The Walter and Eliza Hall Institute. All mice are on, or backcrossed to, the C57BL/6 background for at least 10 generation. Male and female mice of 6–8 weeks old were used. Mice were bred and maintained at The Australian National University under specific pathogen-free conditions. All animal studies were conducted in accordance with the Protocol Number A2020/19 approved by The Australian National University Animal Ethics Committee.

Mammalian cell culture

Primary bone marrow-derived macrophages (BMDMs) were cultured for 5–6 days in DMEM (11995073, ThermoFisher Scientific) supplemented with 10% FBS (F8192, Sigma), 30% L929 conditioned media and 1% penicillin and streptomycin (10378016, Gibco ThermoFisher) as described previously (Man *et al*, 2016). BMDMs were seeded in antibiotic-free media at a concentration of 1×10^6 cells per well in 12-well plates. THP-1 cells were cultured in Rosewell Park Memorial Institute (RPMI)-1640 media (11875093, ThermoFisher Scientific) supplemented with 10% FBS, 1% penicillin and streptomycin, and 1% L-Glutamine (A2916801, Gibco™ ThermoFisher Scientific) at 37°C in 5% CO_2. To induce differentiation of THP-1 monocytes into adherent macrophages, seeded cells were treated with 100 nM phorbol 12-myristate 13-acetate (PMA; 16561-29-8, Sigma-Aldrich) for 24 h and then rested in fresh PMA-free media for another 24 h. Cells were seeded in antibiotic-free media at a concentration of 1×10^6 cells per well in 12-well plates.

Peripheral blood mononuclear cells (PBMCs)

Human blood was obtained with consent from healthy donors in accordance with the Declaration of Helsinki, under protocols approved by the Human Research Ethics Committee of The Australian National University (Protocol 2016/317). PBMCs were isolated from blood by density gradient centrifugation over Lymphoprep™ (07851, STEMCELL Technologies) and suspended at 1×10^7 cells/ml in RPMI-1640 supplemented with 10% FBS, 1% penicillin and streptomycin, and 1% L-Glutamine. PBMCs were seeded in antibiotic-free media at a concentration of 2×10^6 cells per well in 48-well plates.

Bacterial culture

Clostridium perfringens (WT), Δ*pfoA C. perfringens* (Δ*pfoA*), Δ*cpa C. perfringens* (Δ*cpa*) and Δ*pfoA*Δ*cpa C. perfringens* (Δ*pfoA*Δ*cpa*) strains were on the JIR325 background and belong to the type A strain (Lyristis *et al*, 1994). The toxin profile consists mainly of lecithinase, as well as some other toxins such as PFO (Awad *et al*, 2001). These bacteria were grown in brain heart infusion (BHI) media (211059, BD Biosciences) inside anaerobic jars for 24 h at 37°C (AG0025A, Thermo Fisher Scientific). Anaerobic gas-generating sachets (AN0025A, Thermo Fisher Scientific) were used to generate anaerobic conditions. *Salmonella* Typhimurium SL1344 was grown in Luria-Bertani (LB) media (244620, BD Biosciences) overnight under aerobic conditions at 37°C. *F. novicida* strain EXO186 (Feng *et al*, 2022) was grown in BBL Trypticase Soy Broth (TSB; 211768, BD) supplemented with 0.2% L-cysteine (BP376-100, Thermo Fisher Scientific) overnight under aerobic conditions at 37°C. Bacteria were then sub-cultured (1:10) into fresh LB the next day, followed by 3-h incubation.

© 2023 The Authors

Stimulation of BMDMs

For stimulation of BMDMs with bacterial toxins, BMDMs primed with 500 ng/ml ultrapure LPS from *E. coli* (ALX-581-014-L002, Enzo Life Sciences) were treated with 1.5 Units/ml of lecithinase (P7633, Sigma) or lecinthinase which had been heat inactivated (at 100°C for 10 min) for 3 h, 0.1 mg/ml of lecithinase (His-tag, CSB-YP314672CMB, Cusabio) for 12 h, 0.6 mg/ml of perfringolysin O (PFO, RPC2043, Biomatik) for 3 h, and 10 μM of Leu-Leu methyl ester hydrobromide (LLOMe, L7393, Sigma-Aldrich) for 5 h. To activate the canonical NLRP3 inflammasome, LPS-primed BMDMs were stimulated with 5 mM ATP (10127531001, Roche) for 1 h or 10 μM nigericin (Nig., N7143, Sigma-Aldrich) for 30 min. To activate caspase-11, 5 μg of LPS (tlrl-smlps, InvivoGen) were resuspended in PBS and mixed with 0.3 μl of Xfect polymer in Xfect reaction buffer (631318, Clontech Laboratories, Inc.). After 30 min, LPS complexes were added to BMDMs in Opti-MEM (31985–070, ThermoFisher Scientific) and left stimulated for 16 h. To activate the NLRC4 inflammasome, BMDMs were infected with *S.* Typhimurium at MOI of 5 for 4 h. To activate the AIM2 inflammasome, BMDMs were infected with *F. novicida* at MOI of 100 for 16 h (Man *et al*, 2015).

For inhibition experiments BMDMs were pretreated for 30 min with the following inhibitors; 20 μM of the selective and potent NLRP3 inhibitor, MCC950 (Coll *et al*, 2015, 2019; Tapia-Abellan *et al*, 2019), 5 mM, 25 mM, 50 mM or 75 mM of potassium chloride (P9541, Sigma-Aldrich), 50 μM of cytochalasin D (Cyto D, C8273, Sigma-Aldrich), 50 μM cytochalasin B (CytoB, C6762, Sigma), 1 μg/ml of Latrunculin B (Lat B, ab144291, Abcam), 5 mM of methyl-β-cyclodextrin (MCD, C4555, Sigma-Aldrich), 100 nM of bafilomycin A (B1793, Sigma-Aldrich), 10 μM of ammonium chloride (A9434, Sigma-Aldrich), 100 nM of concanamycin A (C9705, Sigma-Aldrich), 20 μM of CA-074 methyl ester (CA-70 Me, C5857, Sigma-Aldrich), 2 μM E-64d (E3132, Sigma-Aldrich), 20 μM of Q-VD-OPh hydrate (QVD, A1901, APEx BIO) and 20 μM of Z-IETD-FMK (Z-ietd, inh-ietd, ThermoFisher Scientific).

For bacterial supernatant, the overnight bacterial culture of *C. perfringens* (WT) and its isogenic mutant strains was centrifuged at 4,000 *g* for 10 min. The supernatant was filter-sterilized using low-protein-binding 0.45-μm filters (SLHV033RS, Merck) to remove the bacteria. LPS-primed BMDMs were stimulated by 200 μl of the supernatant of *C. perfringens* (WT) and its isogenic mutant strains for 20 h. For infection experiments, BMDMs were stimulated with *C. perfringens* WT and mutant strains (MOI 100) for 20 h. Cell culture supernatants were collected for ELISA and LDH assays. Cell culture supernatants and cell lysates were collected for immunoblot analysis.

Stimulation of THP1 cells and PBMCs

WT THP-1 cells and THP-1 cells with a genetic deletion of the gene encoding NLRP3 were sourced from InvivoGen (thp-konlrp3z, InvivoGen). THP-1 monocytes and macrophages were primed using 500 ng/ml ultrapure LPS from *E. coli* for 3 h. Cells were treated with 36 μg/ml of lecithinase or 10 μM nigericin. For inhibition experiments, THP1 cells were pretreated for 30 min with 20 μM of MCC950 (Enosi Tuipulotu *et al*, 2023). PBMCs were primed using 1 μg/ml Pam3CSK4 (tlrl-pms, InvivoGen) for 3 h, pretreated with or without 20 μM MCC950 for 30 min, and then stimulated with 1.5×10^{-3} Units/ml lecithinase or 10 μM nigericin (N7143, Sigma) for 1 h. Cell culture supernatants were collected for ELISA and LDH assays. Cell culture supernatants and cell lysates were collected for immunoblot analysis.

Immunoblotting analysis

For caspase-1 immunoblotting, BMDMs and supernatant were lysed in lysis buffer and sample loading buffer containing SDS and 100 mM DTT. Proteins were separated on 8–12% polyacrylamide gels. Following electrophoretic transfer of proteins onto PVDF membranes (IPVH00010, Millipore), membranes were blocked in 5% skim milk in TBST and incubated overnight with primary antibodies against mouse caspase-1 (1:1,000 dilution, AG-20B-0042, Adipogen), human caspase-1 (1:1,000 dilution, 3866, Cell Signaling Technologies), mouse GSDMD (1:3,000 dilution, ab209845, Abcam), human GSDMD (1:1,000 dilution, ab215203, Abcam), NINJ1 (1:1,000 dilution, A16406, ABclonal), CD14 (1:1,000 dilution, 17000-1-AP, Proteintech), and GAPDH (1:10,000 dilution, 5174, Cell Signaling Technologies). PVDF membranes were then incubated with HRP-conjugated secondary antibody (1:5,000) for 1 h and proteins were visualized using the Super Signal Femto substrate (34095, ThermoFisher Scientific) and the ChemiDoc™ Touch Imaging System (BioRad).

Fluorescent labeling of lecithinase

Alexa Fluor 568 Protein Labeling Kit (A10238, Invitrogen) was used to label lecithinase for immunofluorescence studies. Briefly, the concentration of lecithinase was determined using the Pierce BCA Protein Assay Kit (23225, ThermoFisher Scientific), and an aliquot of lecithinase was diluted to 2 mg/ml in 500 μl ddH_2O 0.1 M bicarbonate (50 μl) was added to give an optimal pH (~ 8.3) for labeling. This 550 μl reaction volume was added to a vial of Alexa Fluor 568 reactive dye and incubated at room temperature for 1 h with gentle rotation. The reactive dye contains a succinimidyl ester moiety that reacts with primary amines of proteins, producing stable protein–dye conjugates. Excess dye was separated from labeled protein by gel filtration using a Bio-Rad BioGel P-30 Fine size exclusion resin. The column was assembled by filling a supplied plastic column with resin until ~ 3 cm from the top and was equilibrated with PBS to ensure proper flow. Next, the labeled protein mixture was added to the column and allowed to settle into the resin, before elution of the protein with elution buffer (10 mM potassium phosphate, 150 mM NaCl, 0.2 mM sodium azide, pH 7.2). The faster running purple band containing labeled lecithinase was collected in 1 ml fractions, whilst the slower running, unincorporated dye was not collected.

Immunofluorescence staining

For immunofluorescence staining to visualize lecithinase, BMDMs were stimulated with 60 μg/ml of Alexa Fluor 568 labeled lecithinase for 2 h with or without inhibitors, 50 μM cytochalasin D or 5 mM MCD, washed three times with PBS and fixed with 4% paraformaldehyde at room temperature for 15 min, followed by blocking in 1% BSA in PBS for 1 h. Cells were incubated with a rat FITC-conjugated anti-CD11b antibody (1:200 dilution in 1% BSA, 101205,

© 2023 The Authors

BioLegend). For visualization of lecithinase with lysosomes, 4% paraformaldehyde fixed cells were blocked with 10% goat serum with 0.1% saponin in PBS for 1 h. Cells were incubated with a rat LAMP1 antibody (1:500 dilution in 10% goat serum with 0.1% saponin) overnight at 4°C. PBS containing 0.05% Tween-20 was used to wash between incubation steps. An anti-rat secondary Alex Fluor 488 antibody (112545143, Jackson ImmunoResearch) was used at 1:500 dilution in 10% goat serum with 0.1% saponin for 1 h at room temperature. The samples were mounted with VECTASHIELD® Hardset™ Mounting Medium with DAPI (H-1500, Vector Laboratories, Inc.) and analyzed using either a Leica SP5 confocal microscope or a Zeiss LSM 800 confocal microscope. Z-stack images of samples were used to generate a three-dimensional structure using the Imaris Viewer software 9.5.

Scanning electron microscopy

Lecithinase- or ATP-treated BMDMs were washed with PBS, fixed with 2.5% glutaraldehyde in PBS overnight and further washed with PBS. Cells were fixed in 1% osmium tetroxide in double distilled water for 1 h and gradually dehydrated in a series of ethanol. Dehydrated samples were transferred to a critical point dryer to replace ethanol by liquid carbon dioxide and dried completely. Samples were then sputter-coated with platinum (3 nm thickness) at 15 mA for 2 min using the EMI TECH K550 Sputter coater and visualized under a Zeiss UltraPlus Field emission scanning electron microscope in secondary electron mode at 5 kV.

Transmission electron microscopy

Lecithinase- or ATP-treated BMDMs were washed with PBS and fixed with 2.5% glutaraldehyde, 2% paraformaldehyde in PBS overnight and further washed with PBS. Cells were postfixed in 1% osmium tetroxide in double distilled water for 1 h and dehydrated in a graded ethanol series and embedded in LR white resin (C023, ProSciTech). Samples were polymerized under N_2 in a 60°C oven overnight. Thin sections were cut at 70 nm and viewed using a Hitachi HA7100 transmission electron microscope at 100 kV.

Correlative light and electron microscopy

WT BMDMs left untreated or treated with AF568-lecithinase for 90 min were washed with PBS. Cells were pelleted and high pressure frozen (Leica EM-ICE). Cells were then freeze substituted and embedded in resin (Leica EM AFS2). The cells were ultramicrotomed at 300 nm thin sections (Leica EM UC7) and then viewed on confocal microscope (ZEISS LSM800) to capture the fluorescence signal within the cells and on scanning electron microscope (Zeiss UltraPlus FE SEM) to capture ultrastructure. The SEM images were captured at 2 kV accelerating voltage using the energy selective backscattered (EsB) detector. After the SEM images were captured, the correlation of AF568-lecithinase into subcellular structures was then performed via a shuttle-and-find system (ZEISS).

Separation of membrane and cytosolic fraction

Untreated or treated BMDMs were washed three times with PBS followed by separation of membrane and cytosolic fractions using the Mem-PER Plus Membrane Protein Extraction Kit according to the manufacturer's instructions (89842, ThermoFisher Scientific).

Flow cytometry

For evaluation of lysosomal destabilization, cells were incubated for 15 min with acridine orange (1 μg/ml, 113000, Sigma-Aldrich), washed three times, and then stimulated with either lecithinase or 1 μM Leu-Leu methyl ester hydrobromide (LLOMe, L7393, Sigma-Aldrich). For inhibition experiments, BMDMs loaded with acridine orange were pretreated for 30 min with inhibitors described earlier. Lysosomal destabilization was assessed by flow cytometry as loss of emission at 600–650 nm. For quantification of the kinetics of lysosomal destabilization and cell death, cells were loaded with acridine orange as described above and then treated with either lecithinase or LLOMe or transfected with LPS over different time points. The cells were washed and resuspended in FACS buffer in the presence or absence of DAPI (0.5 μg/ml, D9542, Sigma-Aldrich). An LSRII (BD Biosciences) was used for flow cytometry. Data were acquired by DIVA (BD Biosciences) and analyzed with the FlowJo v10.7 software (Tree Star).

Liposome studies

Liposomes were synthesized as described previously (Mathur *et al*, 2019) and left untreated or treated with 1.5 Units/ml of lecithinase, the solvent containing lecithinase (ddH_2O) or heat inactivated lecithinase (treated at 100°C for 10 min), or BSA (1 μg/ml), or treated with Alexa Fluor 568 labeled lecithinase for 1 h at 4°C. The liposomes were sonicated at 100 amplitude for 5 min as control (CTRL). The released dye was captured by a cation exchanger resin Dowex (10–15 mg per well). The absorbance (OD) of residual dye was measured at a wavelength of 595 nm using the Infinite 200 PRO system (Tecan).

Lactate dehydrogenase assay

Levels of lactate dehydrogenase released by cells were determined using the CytoTox 96 Non-Radioactive Cytotoxicity Assay according to the manufacturer's instructions (G1780, Promega).

Incucyte analysis

BMDMs were stimulated with either 1.5 Units/ml of lecithinase, 5 mM ATP, or 5 μg of transfected LPS, in the presence of the SYTOX Green nuclear stain that penetrates compromised plasma membranes (1 μM; S7020; Life Technologies). Cell death was monitored over 3–16 h using the IncuCyte Zoom imaging system (Essen Biosciences).

Cytokine analysis

Cytokine concentrations from BMDMs were quantified using a multiplex ELISA (MCYTOMAG-70 K, EMD Millipore) and IL-18 ELISA (BMS618-3TEN, ThermoFisher) according to the manufacturers' instructions. Cytokine concentrations from THP-1 cells and PBMCs were quantified using a human IL-1β ELISA (BMS224-2TEN, ThermoFisher) or human IL-18 ELISA (BMS267-2TEN, ThermoFisher) according to the manufacturer's instructions.

 © 2023 The Authors

ICP-OES analysis

The intracellular concentrations of K^+ ions were determined by inductively coupled plasma-optical emission spectroscopy (ICP-OES) analysis. In brief, BMDMs were stimulated with recombinant 1.5 Units/ml of lecithinase for 2 h or 5 mM ATP for 30 min or 10 μM nigericin for 30 min, washed three times with PBS followed by lysis with concentrated nitric acid (HNO_3). For inhibition experiments, BMDMs were pretreated for 30 min with inhibitors described earlier. The cell lysates were analyzed using a PerkinElmer OPTIMA 7300 ICP Optical Emission Spectrometer (PerkinElmer).

Animal studies

To investigate the physiological role of the inflammasome pathway during *C. perfringens* infection, WT, *Nlrp3*$^{-/-}$ and *Casp1/11*$^{-/-}$ mice were injected via an intraperitoneal route with 1×10^8 CFUs of bacteria. The mice were monitored and ethically culled at a defined humane endpoint for conducting cytokine measurement analysis in serum and peritoneal fluid of the infected mice. To evaluate contributions from PFO and lecithinase in driving inflammation in host, WT mice were injected intraperitoneally with 4×10^8 CFUs of *C. perfringens* (WT), Δ*pfoA C. perfringens* (Δ*pfoA*), Δ*cpa C. perfringens* (Δ*cpa*) and Δ*pfoA*Δ*cpa C. perfringens* (Δ*pfoA*Δ*cpa*) strains and assessed for cytokine levels in the serum and peritoneal fluid. For survival analysis, 8-week-old WT, *Nlrp3*$^{-/-}$ and *Casp1*$^{-/-}$ mice were injected via an intraperitoneal route with 0.312 Units/ml of lecithinase, monitored and ethically culled at a defined humane endpoint. For cytokine measurement in serum and peritoneal fluid, mice were injected via an intraperitoneal route with 0.625 Units/ml of lecithinase. To investigate the effects of MCC950, mice were injected, via an intraperitoneal route, with 50 mg/kg of MCC950 dissolved in PBS or with vehicle control PBS. 1 h later, mice were injected, via an intraperitoneal route, with 0.625 (for survival analysis) or 0.312 (for cytokine measurement) Units/ml of lecithinase along with a second dose of 50 mg/kg of MCC950 or a second dose of PBS. The peritoneal fluid and serum were collected after 2–8 h for analysis by ELISA.

Statistical analysis

The GraphPad Prism 9.0 software was used for data analysis. Data are shown as mean ± s.e.m. Statistical significance was determined by *t*-test (two-tailed) for two groups or One-way ANOVA (with Dunnett's or Tukey's multiple comparisons tests) for three or more groups. $P < 0.05$ was considered statistically significant. No statistical methods were used to calculate sample size.

Data availability

All data are available in the main text or the supplementary materials. No primary datasets have been generated and deposited.

Expanded View for this article is available online.

Acknowledgments
The authors thank members of the Man lab for suggestions and technical assistance, V. M. Dixit (Genentech, USA), K. Schroder (University of Queensland, Australia). K. Lawlor (Hudson Institute of Medical Research, Australia), J. Vince (The Walter and Eliza Hall Institute, Australia), and J. Murphy (The Walter and Eliza Hall Institute, Australia) for reagents, and H. Vohra (ANU Australia), C. Gillespie (ANU, Australia), H. Chen (ANU, Australia), H. Sohail (ANU, Australia), D. Webb (ANU, Australia) and N.-H. Kim (ANU Australia) for technical assistance. The authors acknowledge the facilities and the scientific and technical assistance of Microscopy Australia at the Centre for Advanced Microscopy (ANU, Australia), a facility that is funded by the University and the Federal Government. Funded by the National Health and Medical Research Council of Australia under Grants APP1141504, APP1162103 and APP1163358 (S.M.M.), the CSL Centenary Fellowship (S.M.M.), the Gretel and Gordon Bootes Medical Research Foundation (A.M.), the John Curtin School of Medical Research International Ph.D. Scholarship (A.M.) and the UNSW Scientia Fellowship (N.O.K.). Open access publishing facilitated by Australian National University, as part of the Wiley - Australian National University agreement via the Council of Australian University Librarians.

Author contributions
Anukriti Mathur: Conceptualization; data curation; formal analysis; investigation; visualization; methodology; writing – original draft; writing – review and editing. **Callum Kay:** Conceptualization; data curation; formal analysis; investigation; visualization; methodology; writing – original draft; writing – review and editing. **Yansong Xue:** Investigation; methodology; writing – review and editing. **Abhimanu Pandey:** Investigation; visualization; methodology; writing – review and editing. **Jiwon Lee:** Investigation; visualization; methodology; writing – review and editing. **Weidong Jing:** Investigation; writing – review and editing. **Daniel Enosi Tuipulotu:** Investigation; writing – review and editing. **Jordan Lo Pilato:** Investigation; writing – review and editing. **Shouya Feng:** Investigation; writing – review and editing. **Chinh Ngo:** Investigation; writing – review and editing. **Anyang Zhao:** Investigation; writing – review and editing. **Cheng Shen:** Investigation; writing – review and editing. **Melanie Rug:** Resources; writing – review and editing. **Lisa A Miosge:** Resources; writing – review and editing. **Ines I Atmosukarto:** Resources; writing – review and editing. **Jason D Price:** Resources; writing – review and editing. **Sidra A Ali:** Resources; writing – review and editing. **Elizabeth E Gardiner:** Resources; writing – review and editing. **Avril AB Robertson:** Resources; writing – review and editing. **Milena M Awad:** Resources; writing – review and editing. **Dena Lyras:** Resources; writing – review and editing. **Nadeem O Kaakoush:** Investigation; writing – review and editing. **Si Ming Man:** Conceptualization; supervision; funding acquisition; writing – original draft; project administration; writing – review and editing.

Disclosure and competing interests statement
I.I.A. is Director of Lipotek, a niche biotech company with a focus on liposome technology. I.I.A. and J.D.P. are shareholders of Lipotek. A.A.B.R. is a named inventor on inflammasome inhibitor patents (WO2018215818, WO2017140778 and WO2016131098). All other authors have no conflict of interest.

References

Aglietti RA, Estevez A, Gupta A, Ramirez MG, Liu PS, Kayagaki N, Ciferri C, Dixit VM, Dueber EC (2016) GsdmD p30 elicited by caspase-11 during pyroptosis forms pores in membranes. *Proc Natl Acad Sci U S A* 113: 7858 – 7863

Allam R, Lawlor KE, Yu EC, Mildenhall AL, Moujalled DM, Lewis RS, Ke F, Mason KD, White MJ, Stacey KJ *et al* (2014) Mitochondrial apoptosis is

© 2023 The Authors

dispensable for NLRP3 inflammasome activation but non-apoptotic caspase-8 is required for inflammasome priming. *EMBO Rep* 15: 982 – 990

Awad MM, Ellemor DM, Boyd RL, Emmins JJ, Rood JI (2001) Synergistic effects of alpha-toxin and perfringolysin O in *Clostridium perfringens*-mediated gas gangrene. *Infect Immun* 69: 7904 – 7910

Bose S, Segovia JA, Somarajan SR, Chang TH, Kannan TR, Baseman JB (2014) ADP-ribosylation of NLRP3 by *Mycoplasma pneumoniae* CARDS toxin regulates inflammasome activity. *mBio* 5: e02186-14

Broz P, Pelegrin P, Shao F (2020) The gasdermins, a protein family executing cell death and inflammation. *Nat Rev Immunol* 20: 143 – 157

Bullifent HL, Moir A, Awad MM, Scott PT, Rood JI, Titball RW (1996) The level of expression of α-toxin by different strains of *Clostridium perfringens* is dependent on differences in promoter structure and genetic background. *Anaerobe* 2: 365 – 371

Campden RI, Zhang Y (2019) The role of lysosomal cysteine cathepsins in NLRP3 inflammasome activation. *Arch Biochem Biophys* 670: 32 – 42

Coll RC, Robertson AA, Chae JJ, Higgins SC, Munoz-Planillo R, Inserra MC, Vetter I, Dungan LS, Monks BG, Stutz A *et al* (2015) A small-molecule inhibitor of the NLRP3 inflammasome for the treatment of inflammatory diseases. *Nat Med* 21: 248 – 255

Coll RC, Hill JR, Day CJ, Zamoshnikova A, Boucher D, Massey NL, Chitty JL, Fraser JA, Jennings MP, Robertson AAB *et al* (2019) MCC950 directly targets the NLRP3 ATP-hydrolysis motif for inflammasome inhibition. *Nat Chem Biol* 15: 556 – 559

Darke SG, King AM, Slack WK (1977) Gas gangrene and related infection: classification, clinical features and aetiology, management and mortality. A report of 88 cases. *Br J Surg* 64: 104 – 112

Deng M, Guo H, Tam JW, Johnson BM, Brickey WJ, New JS, Lenox A, Shi H, Golenbock DT, Koller BH *et al* (2019) Platelet-activating factor (PAF) mediates NLRP3-NEK7 inflammasome induction independently of PAFR. *J Exp Med* 216: 2838 – 2853

Dostert C, Petrilli V, Van Bruggen R, Steele C, Mossman BT, Tschopp J (2008) Innate immune activation through Nalp3 inflammasome sensing of asbestos and silica. *Science* 320: 674 – 677

Enosi Tuipulotu D, Feng S, Pandey A, Zhao A, Ngo C, Mathur A, Lee J, Shen C, Fox D, Xue Y *et al* (2023) Immunity against *Moraxella catarrhalis* requires guanylate-binding proteins and caspase-11- NLRP3 inflammasomes. *EMBO J* 42: e112558

Enosi Tuipulotu D, Mathur A, Ngo C, Man SM (2021) *Bacillus cereus*: epidemiology, virulence factors, and host-pathogen interactions. *Trends Microbiol* 29: 458 – 471

Evavold CL, Hafner-Bratkovic I, Devant P, D'Andrea JM, Ngwa EM, Borsic E, Doench JG, LaFleur MW, Sharpe AH, Thiagarajah JR *et al* (2021) Control of gasdermin D oligomerization and pyroptosis by the Ragulator-rag-mTORC1 pathway. *Cell* 184: 4495 – 4511.e19

Feng S, Enosi Tuipulotu D, Pandey A, Jing W, Shen C, Ngo C, Tessema MB, Li FJ, Fox D, Mathur A *et al* (2022) Pathogen-selective killing by guanylate-binding proteins as a molecular mechanism leading to inflammasome signaling. *Nat Commun* 13: 4395

Fox D, Mathur A, Xue Y, Liu Y, Tan WH, Feng S, Pandey A, Ngo C, Hayward JA, Atmosukarto II *et al* (2020) *Bacillus cereus* non-haemolytic enterotoxin activates the NLRP3 inflammasome. *Nat Commun* 11: 760

Frank D, Vince JE (2019) Pyroptosis versus necroptosis: similarities, differences, and crosstalk. *Cell Death Differ* 26: 99 – 114

Franklin BS, Bossaller L, De Nardo D, Ratter JM, Stutz A, Engels G, Brenker C, Nordhoff M, Mirandola SR, Al-Amoudi A *et al* (2014) The adaptor ASC has extracellular and 'prionoid' activities that propagate inflammation. *Nat Immunol* 15: 727 – 737

Gharieb R, Saad M, Abdallah K, Khedr M, Farag E, Abd El-Fattah A (2021) Insights on toxin genotyping, virulence, antibiogram profiling, biofilm formation and efficacy of disinfectants on biofilms of *Clostridium perfringens* isolated from poultry, animals and humans. *J Appl Microbiol* 130: 819 – 831

Greaney AJ, Leppla SH, Moayeri M (2015) Bacterial exotoxins and the Inflammasome. *Front Immunol* 6: 570

Gurung P, Anand PK, Malireddi RK, Vande Walle L, Van Opdenbosch N, Dillon CP, Weinlich R, Green DR, Lamkanfi M, Kanneganti TD (2014) FADD and caspase-8 mediate priming and activation of the canonical and noncanonical Nlrp3 inflammasomes. *J Immunol* 192: 1835 – 1846

Halle A, Hornung V, Petzold GC, Stewart CR, Monks BG, Reinheckel T, Fitzgerald KA, Latz E, Moore KJ, Golenbock DT (2008) The NALP3 inflammasome is involved in the innate immune response to amyloid-beta. *Nat Immunol* 9: 857 – 865

Hart PD, Young MR (1991) Ammonium chloride, an inhibitor of phagosome-lysosome fusion in macrophages, concurrently induces phagosome-endosome fusion, and opens a novel pathway: studies of a pathogenic *Mycobacterium* and a nonpathogenic yeast. *J Exp Med* 174: 881 – 889

Hayward JA, Mathur A, Ngo C, Man SM (2018) Cytosolic recognition of microbes and pathogens: inflammasomes in action. *Microbiol Mol Biol Rev* 82: e00015-18

He WT, Wan H, Hu L, Chen P, Wang X, Huang Z, Yang ZH, Zhong CQ, Han J (2015) Gasdermin D is an executor of pyroptosis and required for interleukin-1beta secretion. *Cell Res* 25: 1285 – 1298

He Y, Hara H, Nunez G (2016) Mechanism and regulation of NLRP3 inflammasome activation. *Trends Biochem Sci* 41: 1012 – 1021

Hofbauer D, Mougiakakos D, Broggini L, Zaiss M, Buttner-Herold M, Bach C, Spriewald B, Neumann F, Bisht S, Nolting J *et al* (2021) Beta2-microglobulin triggers NLRP3 inflammasome activation in tumor-associated macrophages to promote multiple myeloma progression. *Immunity* 54: 1772 – 1787.e9

Hornung V, Bauernfeind F, Halle A, Samstad EO, Kono H, Rock KL, Fitzgerald KA, Latz E (2008) Silica crystals and aluminum salts activate the NALP3 inflammasome through phagosomal destabilization. *Nat Immunol* 9: 847 – 856

Huss M, Ingenhorst G, Konig S, Gassel M, Drose S, Zeeck A, Altendorf K, Wieczorek H (2002) Concanamycin a, the specific inhibitor of V-ATPases, binds to the V(o) subunit c. *J Biol Chem* 277: 40544 – 40548

Jing W, Lo Pilato J, Kay C, Man SM (2021) Activation mechanisms of inflammasomes by bacterial toxins. *Cell Microbiol* 23: e13309

Jing W, Pilato JL, Kay C, Feng S, Tuipulotu DE, Mathur A, Shen C, Ngo C, Zhao A, Miosge LA *et al* (2022) *Clostridium septicum* alpha-toxin activates the NLRP3 inflammasome by engaging GPI-anchored proteins. *Sci Immunol* 7: eabm1803

Jones JW, Kayagaki N, Broz P, Henry T, Newton K, O'Rourke K, Chan S, Dong J, Qu Y, Roose-Girma M *et al* (2010) Absent in melanoma 2 is required for innate immune recognition of *Francisella tularensis*. *Proc Natl Acad Sci U S A* 107: 9771 – 9776

Kayagaki N, Stowe IB, Lee BL, O'Rourke K, Anderson K, Warming S, Cuellar T, Haley B, Roose-Girma M, Phung QT *et al* (2015) Caspase-11 cleaves gasdermin D for non-canonical inflammasome signalling. *Nature* 526: 666 – 671

Kayagaki N, Kornfeld OS, Lee BL, Stowe IB, O'Rourke K, Li Q, Sandoval W, Yan D, Kang J, Xu M *et al* (2021) NINJ1 mediates plasma membrane rupture during lytic cell death. *Nature* 591: 131 – 136

Keyburn AL, Sheedy SA, Ford ME, Williamson MM, Awad MM, Rood JI, Moore RJ (2006) Alpha-toxin of *Clostridium perfringens* is not an essential

 © 2023 The Authors

virulence factor in necrotic enteritis in chickens. *Infect Immun* 74: 6496–6500

Kovarova M, Hesker PR, Jania L, Nguyen M, Snouwaert JN, Xiang Z, Lommatzsch SE, Huang MT, Ting JP, Koller BH (2012) NLRP1-dependent pyroptosis leads to acute lung injury and morbidity in mice. *J Immunol* 189: 2006–2016

Kuida K, Lippke JA, Ku G, Harding MW, Livingston DJ, Su MS, Flavell RA (1995) Altered cytokine export and apoptosis in mice deficient in interleukin-1 beta converting enzyme. *Science* 267: 2000–2003

Lee MS, Kwon H, Lee EY, Kim DJ, Park JH, Tesh VL, Oh TK, Kim MH (2016) Shiga toxins activate the NLRP3 Inflammasome pathway to promote both production of the Proinflammatory cytokine interleukin-1beta and Apoptotic cell death. *Infect Immun* 84: 172–186

Leiblein M, Wagner N, Adam EH, Frank J, Marzi I, Nau C (2020) Clostridial gas gangrene – a rare but deadly infection: case series and comparison to other necrotizing soft tissue infections. *Orthop Surg* 12: 1733–1747

Lima HJr, Jacobson LS, Goldberg MF, Chandran K, Diaz-Griffero F, Lisanti MP, Brojatsch J (2013) Role of lysosome rupture in controlling Nlrp3 signaling and necrotic cell death. *Cell Cycle* 12: 1868–1878

Liston A, Masters SL (2017) Homeostasis-altering molecular processes as mechanisms of inflammasome activation. *Nat Rev Immunol* 17: 208–214

Luzio JP, Pryor PR, Bright NA (2007) Lysosomes: fusion and function. *Nat Rev Mol Cell Biol* 8: 622–632

Lyristis M, Bryant AE, Sloan J, Awad MM, Nisbet IT, Stevens DL, Rood JI (1994) Identification and molecular analysis of a locus that regulates extracellular toxin production in *Clostridium perfringens*. *Mol Microbiol* 12: 761–777

Man SM (2018) Inflammasomes in the gastrointestinal tract: infection, cancer and gut microbiota homeostasis. *Nat Rev Gastroenterol Hepatol* 15: 721–737

Man SM, Kanneganti TD (2016) Converging roles of caspases in inflammasome activation, cell death and innate immunity. *Nat Rev Immunol* 16: 7–21

Man SM, Karki R, Malireddi RKS, Neale G, Vogel P, Yamamoto M, Lamkanfi M, Kanneganti T-D (2015) The transcription factor IRF1 and guanylate-binding proteins target activation of the AIM2 inflammasome by *Francisella* infection. *Nat Immunol* 16: 467–475

Man SM, Karki R, Sasai M, Place DE, Kesavardhana S, Temirov J, Frase S, Zhu Q, Malireddi RKS, Kuriakose T *et al* (2016) IRGB10 liberates bacterial ligands for sensing by the AIM2 and Caspase-11-NLRP3 Inflammasomes. *Cell* 167: 382–396.e17

Mariathasan S, Newton K, Monack DM, Vucic D, French DM, Lee WP, Roose-Girma M, Erickson S, Dixit VM (2004) Differential activation of the inflammasome by caspase-1 adaptors ASC and Ipaf. *Nature* 430: 213–218

Martinschek A, Evers B, Lampl L, Gerngross H, Schmidt R, Sparwasser C (2012) Prognostic aspects, survival rate, and predisposing risk factors in patients with Fournier's gangrene and necrotizing soft tissue infections: evaluation of clinical outcome of 55 patients. *Urol Int* 89: 173–179

Mathur A, Hayward JA, Man SM (2018) Molecular mechanisms of inflammasome signaling. *J Leukoc Biol* 103: 233–257

Mathur A, Feng S, Hayward JA, Ngo C, Fox D, Atmosukarto II, Price JD, Schauer K, Martlbauer E, Robertson AAB *et al* (2019) A multicomponent toxin from *Bacillus cereus* incites inflammation and shapes host outcome via the NLRP3 inflammasome. *Nat Microbiol* 4: 362–374

Mauvezin C, Neufeld TP (2015) Bafilomycin A1 disrupts autophagic flux by inhibiting both V-ATPase-dependent acidification and Ca-P60A/SERCA-dependent autophagosome-lysosome fusion. *Autophagy* 11: 1437–1438

McHenry CR, Piotrowski JJ, Petrinic D, Malangoni MA (1995) Determinants of mortality for necrotizing soft-tissue infections. *Ann Surg* 221: 558–563; discussion 563–555

Munoz-Planillo R, Franchi L, Miller LS, Nunez G (2009) A critical role for hemolysins and bacterial lipoproteins in *Staphylococcus aureus*-induced activation of the Nlrp3 inflammasome. *J Immunol* 183: 3942–3948

Munoz-Planillo R, Kuffa P, Martinez-Colon G, Smith BL, Rajendiran TM, Nunez G (2013) K^+ efflux is the common trigger of NLRP3 inflammasome activation by bacterial toxins and particulate matter. *Immunity* 38: 1142–1153

Murphy JM (2020) The killer Pseudokinase mixed lineage kinase domain-like protein (MLKL). *Cold Spring Harb Perspect Biol* 12: a036376

Murphy JM, Czabotar PE, Hildebrand JM, Lucet IS, Zhang JG, Alvarez-Diaz S, Lewis R, Lalaoui N, Metcalf D, Webb AI *et al* (2013) The pseudokinase MLKL mediates necroptosis via a molecular switch mechanism. *Immunity* 39: 443–453

Myers GS, Rasko DA, Cheung JK, Ravel J, Seshadri R, DeBoy RT, Ren Q, Varga J, Awad MM, Brinkac LM *et al* (2006) Skewed genomic variability in strains of the toxigenic bacterial pathogen, *Clostridium perfringens*. *Genome Res* 16: 1031–1040

Pandey A, Shen C, Feng S, Man SM (2021) Cell biology of inflammasome activation. *Trends Cell Biol* 31: 924–939

Perret M, Badiou C, Lina G, Burbaud S, Benito Y, Bes M, Cottin V, Couzon F, Juruj C, Dauwalder O *et al* (2012) Cross-talk between *Staphylococcus aureus* leukocidins-intoxicated macrophages and lung epithelial cells triggers chemokine secretion in an inflammasome-dependent manner. *Cell Microbiol* 14: 1019–1036

Petrilli V, Papin S, Dostert C, Mayor A, Martinon F, Tschopp J (2007) Activation of the NALP3 inflammasome is triggered by low intracellular potassium concentration. *Cell Death Differ* 14: 1583–1589

Pierzynska-Mach A, Janowski PA, Dobrucki JW (2014) Evaluation of acridine orange, LysoTracker red, and quinacrine as fluorescent probes for long-term tracking of acidic vesicles. *Cytometry A* 85: 729–737

Qi X, Man SM, Malireddi RK, Karki R, Lupfer C, Gurung P, Neale G, Guy CS, Lamkanfi M, Kanneganti TD (2016) Cathepsin B modulates lysosomal biogenesis and host defense against *Francisella novicida* infection. *J Exp Med* 213: 2081–2097

Rashidi M, Simpson DS, Hempel A, Frank D, Petrie E, Vince A, Feltham R, Murphy J, Chatfield SM, Salvesen GS *et al* (2019) The pyroptotic cell death effector Gasdermin D is activated by gout-associated uric acid crystals but is dispensable for cell death and IL-1beta release. *J Immunol* 203: 736–748

Rathinam VA, Fitzgerald KA (2016) Inflammasome complexes: emerging mechanisms and effector functions. *Cell* 165: 792–800

Rauch I, Deets KA, Ji DX, von Moltke J, Tenthorey JL, Lee AY, Philip NH, Ayres JS, Brodsky IE, Gronert K *et al* (2017) NAIP-NLRC4 Inflammasomes coordinate intestinal epithelial cell expulsion with eicosanoid and IL-18 release via activation of Caspase-1 and -8. *Immunity* 46: 649–659

Rood JI, Adams V, Lacey J, Lyras D, McClane BA, Melville SB, Moore RJ, Popoff MR, Sarker MR, Songer JG *et al* (2018) Expansion of the *Clostridium perfringens* toxin-based typing scheme. *Anaerobe* 53: 5–10

Ruhl S, Broz P (2015) Caspase-11 activates a canonical NLRP3 inflammasome by promoting K(+) efflux. *Eur J Immunol* 45: 2927–2936

Sborgi L, Ruhl S, Mulvihill E, Pipercevic J, Heilig R, Stahlberg H, Farady CJ, Muller DJ, Broz P, Hiller S (2016) GSDMD membrane pore formation constitutes the mechanism of pyroptotic cell death. *EMBO J* 35: 1766–1778

Schroder K, Tschopp J (2010) The inflammasomes. *Cell* 140: 821–832

Shi J, Zhao Y, Wang K, Shi X, Wang Y, Huang H, Zhuang Y, Cai T, Wang F, Shao F (2015) Cleavage of GSDMD by inflammatory caspases determines pyroptotic cell death. *Nature* 526: 660–665

Stevens DL, Bryant AE (2018) Necrotizing soft-tissue infections. *N Engl J Med* 378: 971

© 2023 The Authors

Stevens DL, Aldape MJ, Bryant AE (2012) Life-threatening clostridial infections. *Anaerobe* 18: 254 – 259

Sun L, Wang H, Wang Z, He S, Chen S, Liao D, Wang L, Yan J, Liu W, Lei X *et al* (2012) Mixed lineage kinase domain-like protein mediates necrosis signaling downstream of RIP3 kinase. *Cell* 148: 213 – 227

Swanson KV, Deng M, Ting JP (2019) The NLRP3 inflammasome: molecular activation and regulation to therapeutics. *Nat Rev Immunol* 19: 477 – 489

Tapia-Abellan A, Angosto-Bazarra D, Martinez-Banaclocha H, de Torre-Minguela C, Ceron-Carrasco JP, Perez-Sanchez H, Arostegui JI, Pelegrin P (2019) MCC950 closes the active conformation of NLRP3 to an inactive state. *Nat Chem Biol* 15: 560 – 564

Tsujimoto K, Jo T, Nagira D, Konaka H, Park JH, Yoshimura SI, Ninomiya A, Sugihara F, Hirayama T, Itotagawa E *et al* (2022) The lysosomal Ragulator complex activates NLRP3 inflammasome *in vivo* via HDAC6. *EMBO J* 42: e111389

Uzal FA, Vidal JE, McClane BA, Gurjar AA (2010) *Clostridium perfringens* toxins involved in mammalian veterinary diseases. *Open Toxinology J* 2: 24 – 42

Uzal FA, Freedman JC, Shrestha A, Theoret JR, Garcia J, Awad MM, Adams V, Moore RJ, Rood JI, McClane BA (2014) Towards an understanding of the role of *Clostridium perfringens* toxins in human and animal disease. *Future Microbiol* 9: 361 – 377

Verherstraeten S, Goossens E, Valgaeren B, Pardon B, Timbermont L, Haesebrouck F, Ducatelle R, Deprez P, Wade KR, Tweten R *et al* (2015) Perfringolysin O: the underrated *Clostridium perfringens* toxin? *Toxins* 7: 1702 – 1721

Vince JE, De Nardo D, Gao W, Vince AJ, Hall C, McArthur K, Simpson D, Vijayaraj S, Lindqvist LM, Bouillet P *et al* (2018) The mitochondrial apoptotic effectors BAX/BAK activate Caspase-3 and -7 to trigger NLRP3 inflammasome and Caspase-8 driven IL-1beta activation. *Cell Rep* 25: 2339 – 2353.e4

Wang S, Miura M, Jung YK, Zhu H, Li E, Yuan J (1998) Murine caspase-11, an ICE-interacting protease, is essential for the activation of ICE. *Cell* 92: 501 – 509

Wang F, Gomez-Sintes R, Boya P (2018) Lysosomal membrane permeabilization and cell death. *Traffic* 19: 918 – 931

Wang X, Eagen WJ, Lee JC (2020) Orchestration of human macrophage NLRP3 inflammasome activation by *Staphylococcus aureus* extracellular vesicles. *Proc Natl Acad Sci U S A* 117: 3174 – 3184

Xue Y, Enosi Tuipulotu D, Tan WH, Kay C, Man SM (2019) Emerging activators and regulators of inflammasomes and pyroptosis. *Trends Immunol* 40: 1035 – 1052

Yamamoto A, Tagawa Y, Yoshimori T, Moriyama Y, Masaki R, Tashiro Y (1998) Bafilomycin A1 prevents maturation of autophagic vacuoles by inhibiting fusion between autophagosomes and lysosomes in rat hepatoma cell line, H-4-II-E cells. *Cell Struct Funct* 23: 33 – 42

Yamamura K, Ashida H, Okano T, Kinoshita-Daitoku R, Suzuki S, Ohtani K, Hamagaki M, Ikeda T, Suzuki T (2019) Inflammasome activation induced by Perfringolysin O of *Clostridium perfringens* and its involvement in the progression of gas gangrene. *Front Microbiol* 10: 2406

Zheng Z, Deng W, Bai Y, Miao R, Mei S, Zhang Z, Pan Y, Wang Y, Min R, Deng F *et al* (2021) The lysosomal rag-Ragulator complex licenses RIPK1– and caspase-8–mediated pyroptosis by *Yersinia*. *Science* 372: eabg0269

License: This is an open access article under the terms of the Creative Commons Attribution License, which permits use, distribution and reproduction in any medium, provided the original work is properly cited.

Expanded View Figures

Figure EV1. PFO activates the NLRP3 inflammasome.

A Immunoblot analysis of caspase-1 (Casp-1) in WT or *Nlrp3*$^{-/-}$ BMDMs left untreated (Medium alone [Med.]) or LPS primed and assessed 4 h after stimulation with bacteria or the supernatant (Sup.) of WT *C. perfringens* (WT *C. per.*), *ΔpfoA C. perfringens* (*ΔpfoA*) or *Δcpa C. perfringens* (*Δcpa*).
B Release of IL-1β (top) and IL-18 (middle), and death (bottom) of BMDMs as treated in (A).
C Immunoblot analysis of caspase-1 in WT or *Nlrp3*$^{-/-}$ BMDMs left untreated or LPS primed and assessed 3 h after stimulation with perfringolysin O (PFO).
D Release of IL-1β (top) and IL-18 (middle), and death (bottom) of BMDMs as treated in (C).

Data information: Each symbol represents an independent biological replicate (B and D). NS, not significant, *$P < 0.05$, and ****$P < 0.0001$ (one-way ANOVA with Dunnett's multiple-comparisons test [B] or two-tailed t-test [D]). Data are representative of three independent biological experiments (A–D; mean and s.e.m. in B and D).
Source data are available online for this figure.

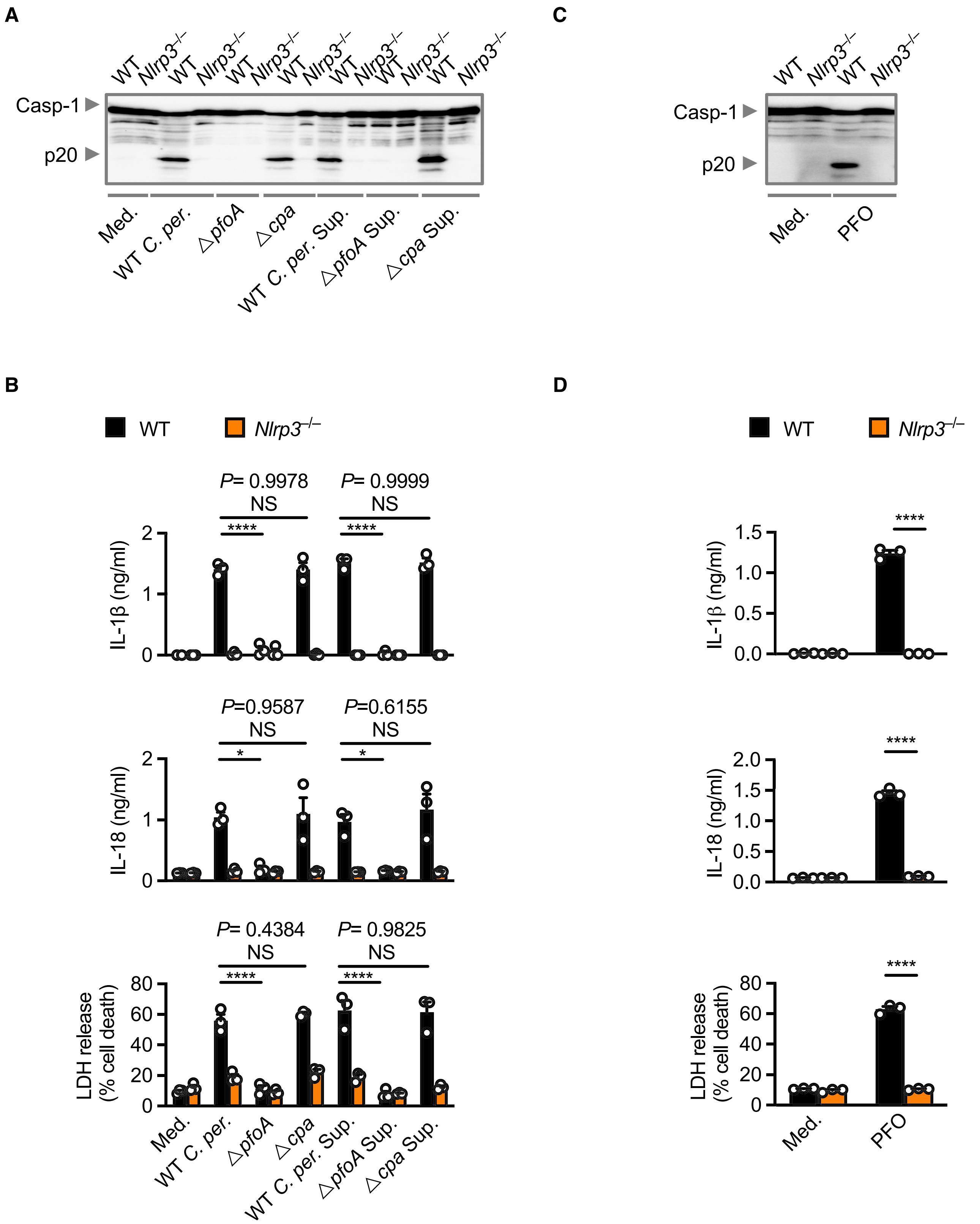

Figure EV1.

 © 2023 The Authors

Figure EV2. Lecithinase activates the NLRP3 inflammasome in murine cells.

A Microscopy analysis of death of BMDMs left untreated [Medium alone (Med.)] or LPS-primed and assessed 3 h after stimulation with lecithinase (Lec.). Arrowheads indicate dead cells.
B IncuCyte live-imaging analysis of the viability of WT or *Nlrp3*$^{-/-}$ BMDMs left untreated or LPS-primed and assessed after stimulation with lecithinase (Lec.) or ATP.
C Release of IL-6 and KC of BMDMs left untreated or LPS-primed and assessed 3 h after stimulation with lecithinase (Lec.).
D Immunoblot analysis of caspase-1 and gasdermin D of WT or *Nlrp3*$^{-/-}$ BMDMs left untreated or LPS-primed and assessed 12 h after stimulation with 0.1 mg/ml an alternatively sourced lecithinase (Cusabio).
E Release of IL-1β (left) and IL-18 (middle), and death (right) of BMDMs after treatment as in (D).
F Concentration of TNF and KC in the serum of WT mice, 8 h after intraperitoneal infection with 4×10^8 colony-forming units (CFUs) of *C. perfringens* (WT *C. perf.*, *n* = 14), *ΔpfoA C. perfringens* (*ΔpfoA*, *n* = 13), *Δcpa C. perfringens* (*Δcpa*, *n* = 11) or *ΔpfoAΔcpa C. perfringens* (*ΔpfoAΔcpa*, *n* = 12).

Data information: Each symbol represents an independent biological replicate (C and E) or represents an individual mouse (F). NS, not significant, $^{**}P < 0.01$, $^{***}P < 0.001$ and $^{****}P < 0.0001$ (two-tailed *t*-test [B and E] or one-way ANOVA with Dunnett's multiple-comparisons test [F]). Data are pooled from two independent biological experiments (B and F) or representative of three independent biological experiments (A, C, D and E; mean and s.e.m. in B, C, E and F).
Source data are available online for this figure.

© 2023 The Authors

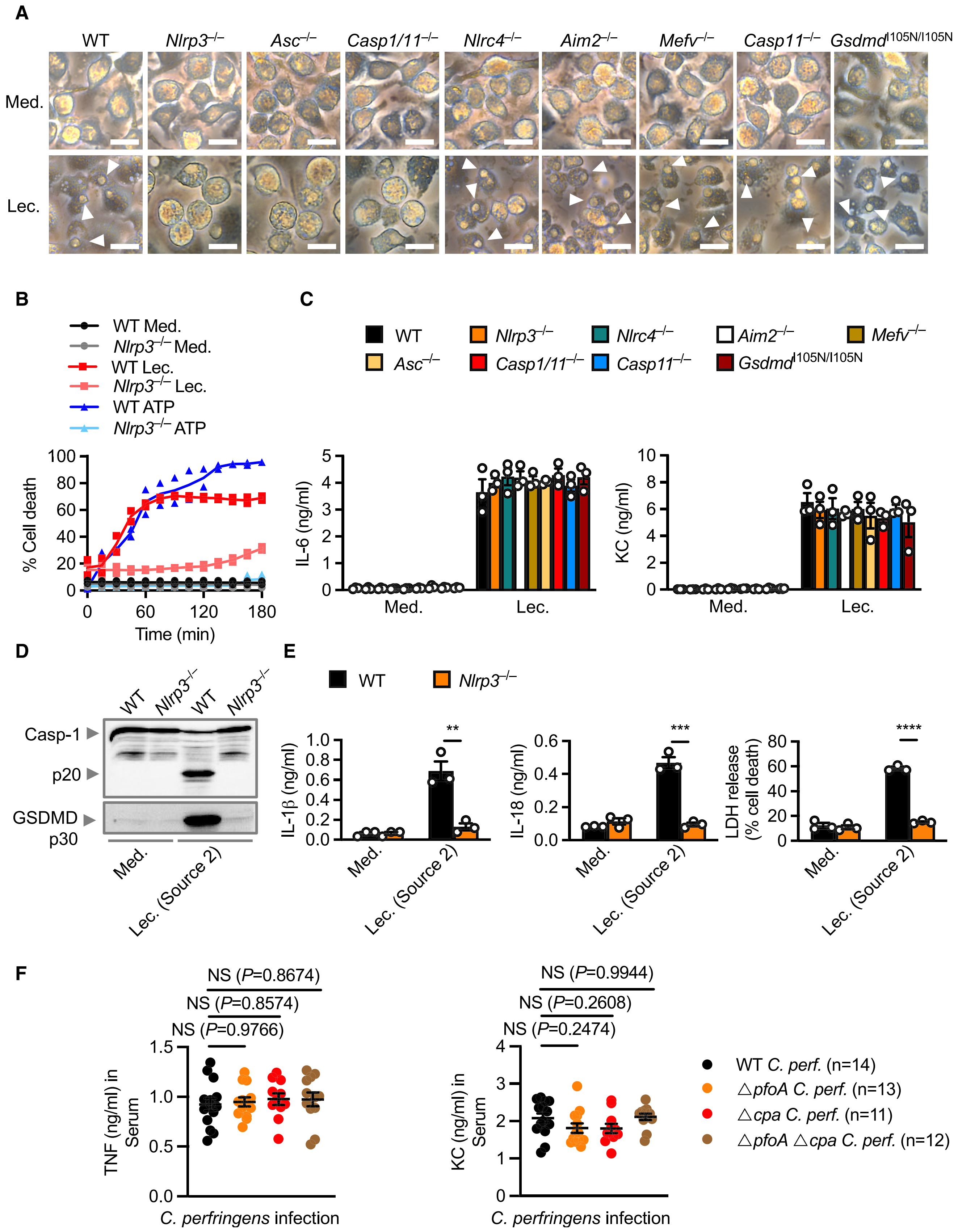

Figure EV2.

 © 2023 The Authors

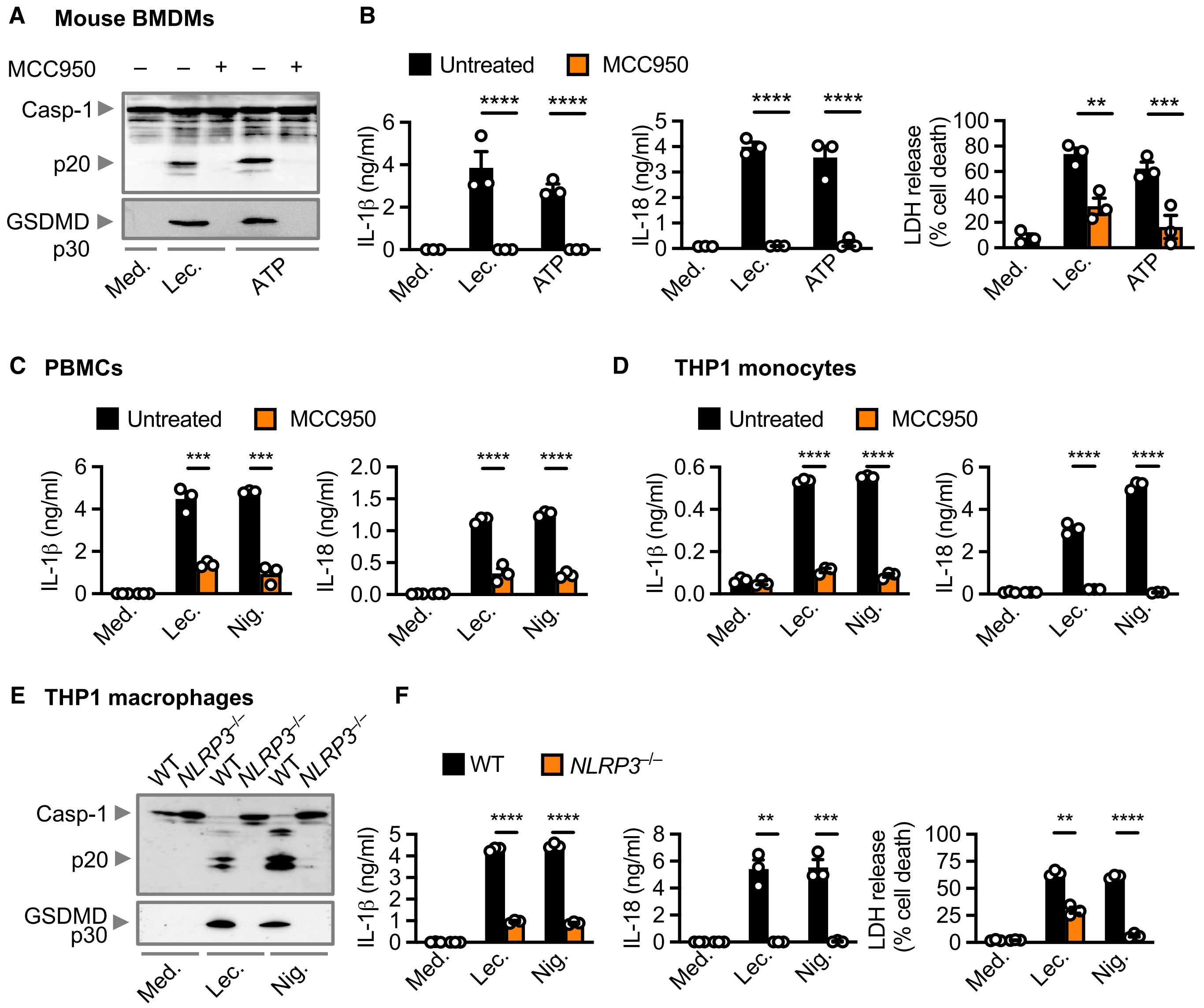

Figure EV3. Lecithinase activates the NLRP3 inflammasome in mouse macrophages, human cell lines and human blood-derived cells.

A Immunoblot analysis of caspase-1 and gasdermin D of WT BMDMs left untreated [Medium alone (Med.)] or LPS-primed and assessed 3 h after stimulation with lecithinase (Lec.) or 1 h after stimulation with ATP in the absence or presence of MCC950 (20 μM).

B Release of IL-1β (left) and IL-18 (middle), and death (right) of BMDMs after treatment as in (A).

C Release of IL-1β (left) and IL-18 (right) of human blood-derived peripheral blood mononuclear cells (PBMCs) from healthy control, left untreated or Pam3CSK4-primed and assessed 3 h after stimulation with lecithinase (Lec.) or 30 min after stimulation with nigericin (Nig.) in the absence or presence of MCC950 (20 μM).

D Release of IL-1β (left) and IL-18 (right) of WT THP-1 monocytes, left untreated or LPS-primed and assessed 3 h after treatment with lecithinase (Lec.) or 30 min nigericin (Nig.) in the absence or presence of MCC950 (20 μM).

E Immunoblot analysis of caspase-1 and gasdermin D of WT or *NLRP3*$^{-/-}$ THP-1 macrophages left untreated or Pam3CSK4-primed and assessed 3 h after stimulation with lecithinase (Lec.) or 30 min after stimulation with nigericin (Nig.).

F Release of IL-1β (left) and IL-18 (middle), and death (right) of THP1 cells as treated in (E).

Data information: Each symbol represents an independent biological replicate (B, C, D and F). NS, not significant, $**P < 0.01$, $***P < 0.001$ and $****P < 0.0001$ (two-tailed *t*-test [B, C, D and F]). Data are representative of three independent biological experiments (A to F; mean and s.e.m. in B, C, D and F).
Source data are available online for this figure.

Figure EV4. Lecithinase-mediated inflammasome activation is independent of lysosomal protease activity.

A Immunoblot analysis of caspase-1 and gasdermin D of WT BMDMs left untreated [Medium alone (Med.)] or LPS-primed and assessed 3 h after stimulation with lecithinase (Lec.) or 30 min after stimulation with nigericin (Nig.) or 5 h after stimulation with LLOMe in the absence or presence of cathepsin B inhibitor (CA-074 Me, 20 μM).

B Release of IL-1β, IL-18, LDH, TNF and KC of WT BMDMs as treated in A.

C Immunoblot analysis of caspase-1 and gasdermin D of WT BMDMs left untreated or LPS-primed and assessed 3 h after stimulation with lecithinase (Lec.) or 30 min after stimulation with nigericin (Nig.) or 5 h after stimulation with LLOMe in the absence or presence of broad spectrum a lysosomal protease inhibitor (E64d, 2 μM).

D Release of IL-1β, IL-18, LDH, TNF and KC of WT BMDMs as treated in C.

Data information: Each symbol represents an independent biological replicate (B and D). NS, not significant. **$P < 0.01$, ***$P < 0.001$ and ****$P < 0.0001$ (two-tailed *t*-test [B and D]). Data are representative of three independent biological experiments (A–D; mean and s.e.m. in B and D).
Source data are available online for this figure.

 © 2023 The Authors

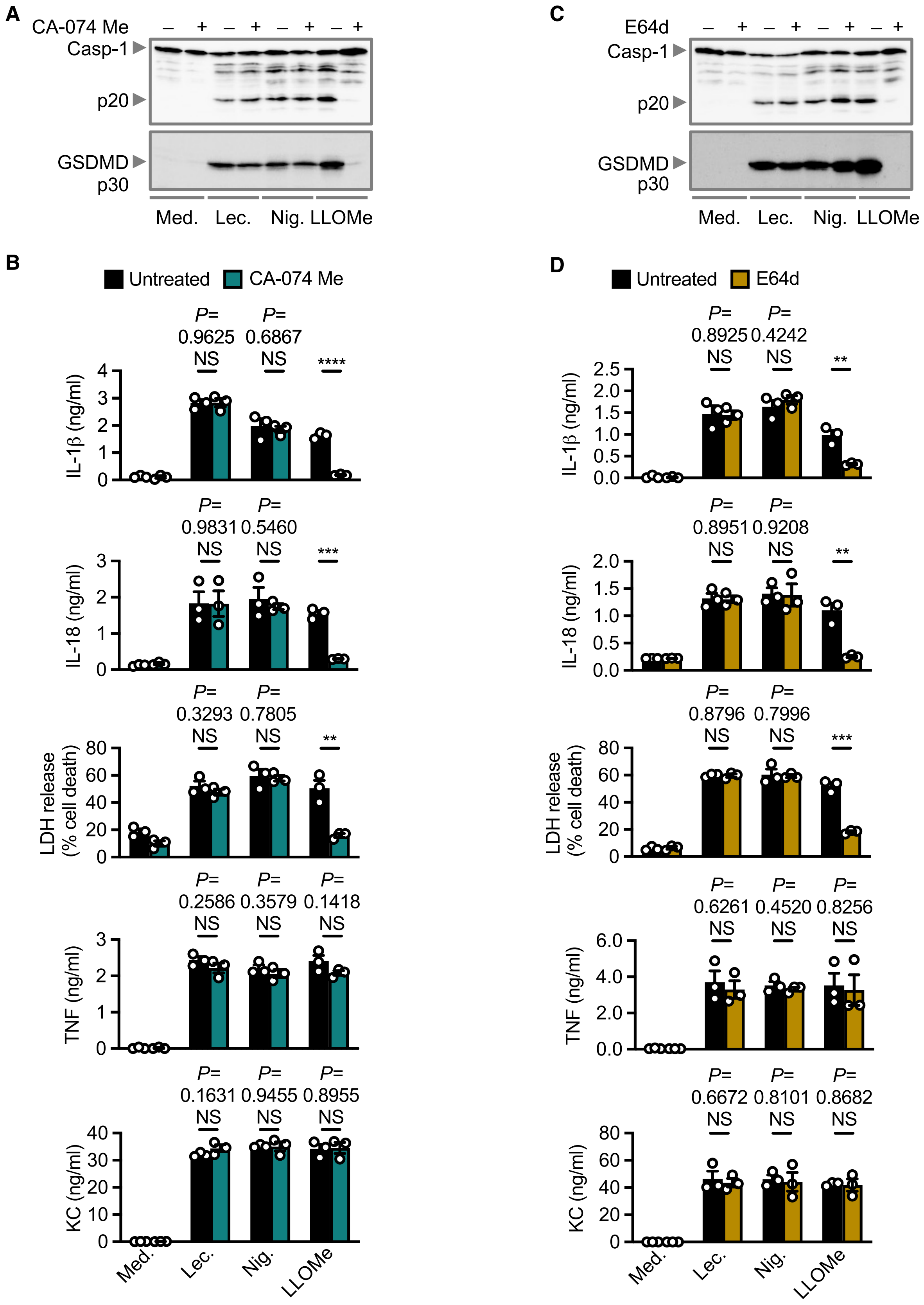
A
CA-074 Me
Casp-1
p20
GSDMD p30
Med. Lec. Nig. LLOMe
C
E64d
Casp-1
p20
GSDMD p30
Med. Lec. Nig. LLOMe
B
Untreated CA-074 Me
P= 0.9625 NS
P= 0.6867 NS
****
IL-1β (ng/ml)
P= 0.9831 NS
P= 0.5460 NS
***
IL-18 (ng/ml)
P= 0.3293 NS
P= 0.7805 NS
**
LDH release (% cell death)
P= 0.2586 NS
P= 0.3579 NS
P= 0.1418 NS
TNF (ng/ml)
P= 0.1631 NS
P= 0.9455 NS
P= 0.8955 NS
KC (ng/ml)
Med. Lec. Nig. LLOMe
D
Untreated E64d
P= 0.8925 NS
P= 0.4242 NS
**
IL-1β (ng/ml)
P= 0.8951 NS
P= 0.9208 NS
**
IL-18 (ng/ml)
P= 0.8796 NS
P= 0.7996 NS
***
LDH release (% cell death)
P= 0.6261 NS
P= 0.4520 NS
P= 0.8256 NS
TNF (ng/ml)
P= 0.6672 NS
P= 0.8101 NS
P= 0.8682 NS
KC (ng/ml)
Med. Lec. Nig. LLOMe

Figure EV4.

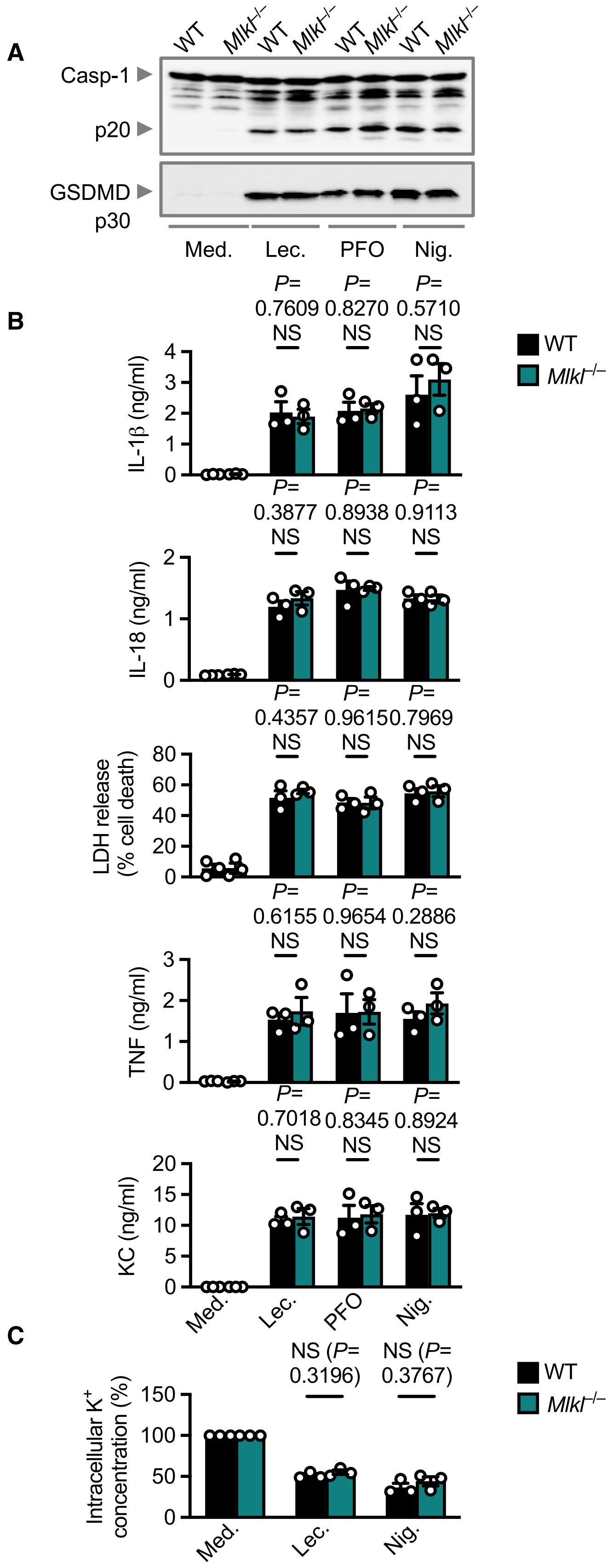

Figure EV5. MLKL is not required for activation of the inflammasome by lecithinase.

A Immunoblot analysis of caspase-1 and gasdermin D in WT or *Mlkl*$^{-/-}$ BMDMs left untreated [Medium alone (Med.)] or LPS-primed and assessed 3 h after stimulation with lecithinase (Lec.) or 3 h after stimulation with perfringolysin O (PFO) or 30 min after stimulation with nigericin (Nig.).

B Release of IL-1β, IL-18, LDH, TNF and KC of WT BMDMs as treated in (A).

C Inductively coupled plasma-optical emission spectrometry analysis of intracellular concentrations of K^+ of BMDMs left untreated or LPS primed and assessed 2 h after stimulation with lecithinase (Lec.), or 30 min after stimulation with nigericin (Nig.).

Data information: Each symbol represents an independent biological replicate (B and C). NS, not significant. (two-tailed *t*-test [B and C]). Data are representative of three independent biological experiments (A–C; mean and s.e.m. in B and C).
Source data are available online for this figure.

 © 2023 The Authors
