# Supplementary material for: Clostridium perfringens virulence factors are nonredundant activators of the NLRP3 inflammasome
Source: EMBO Rep. 2023 Apr 19;24(6):e54600. doi: 10.15252/embr.202254600 (PMC10240202; doi:10.15252/embr.202254600)

Figure 1A

- WT, *Nlrp3*$^{-/-}$ BMDMs
- Media, WT *C. perfringens* infection, △*pfoA* infection, △*cpa* infection, △*pfoA* △*cpa* infection

Caspase-1

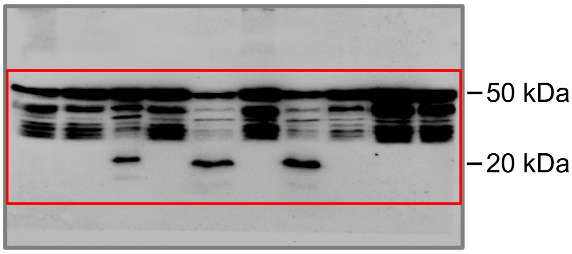

GSDMD

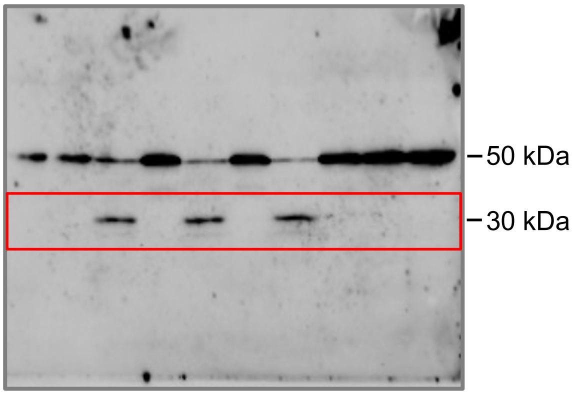

Caspase-1

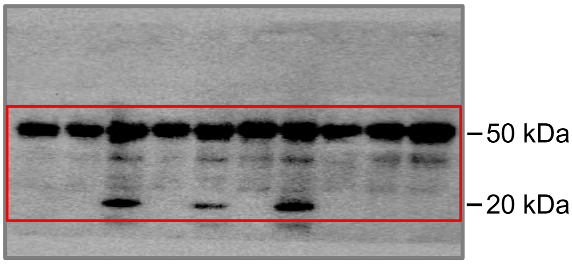

GSDMD

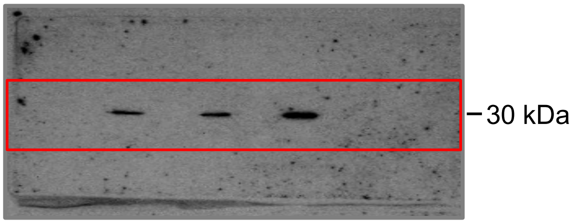

Supplement: Supplementary file 5 — Source Data for Figure 1 [file EMBR-24-e54600-s008.zip › Figure 1/Fig 1A western blot.pdf]
