# Supplementary material for: Clostridium perfringens virulence factors are nonredundant activators of the NLRP3 inflammasome
Source: EMBO Rep. 2023 Apr 19;24(6):e54600. doi: 10.15252/embr.202254600 (PMC10240202; doi:10.15252/embr.202254600)

Figure 3H

- Untreated/LatB-treated WT BMDMs
- Media, lecithinase, *S.* Typhimurium, nigericin

Caspase-1

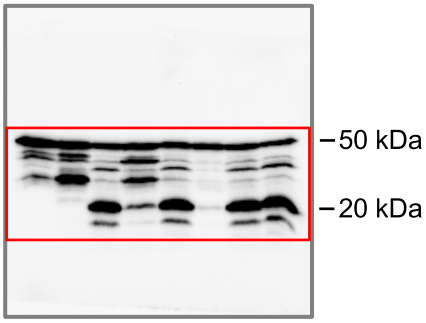

GSDMD

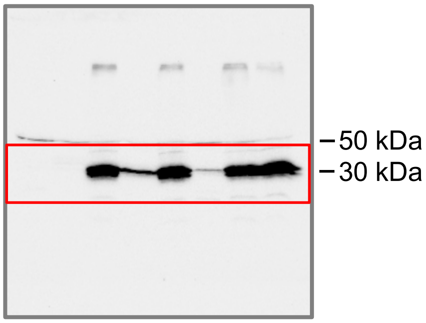

Supplement: Supplementary file 7 — Source Data for Figure 3 [file EMBR-24-e54600-s011.zip › Figure 3/Fig 3H western blot.pdf]
