# Supplementary material for: Clostridium perfringens virulence factors are nonredundant activators of the NLRP3 inflammasome
Source: EMBO Rep. 2023 Apr 19;24(6):e54600. doi: 10.15252/embr.202254600 (PMC10240202; doi:10.15252/embr.202254600)

Figure 3A

- Media
- Merge/DAPI

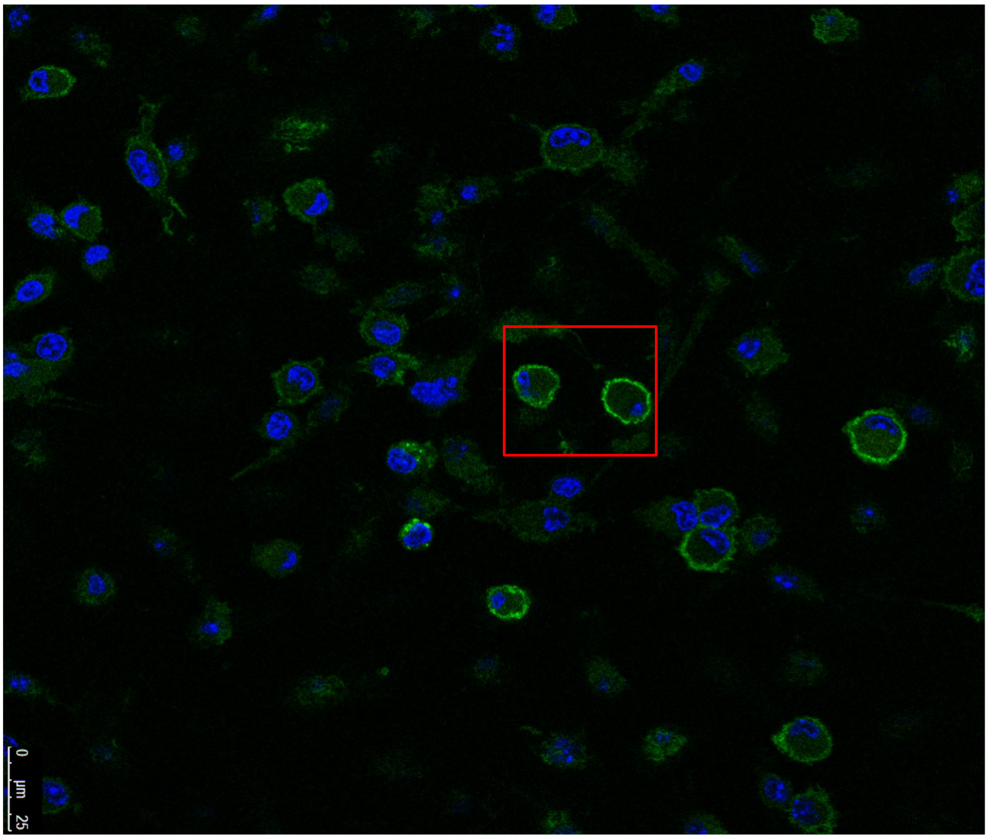

Figure 3A

- Media
- Lec.+ DAPI

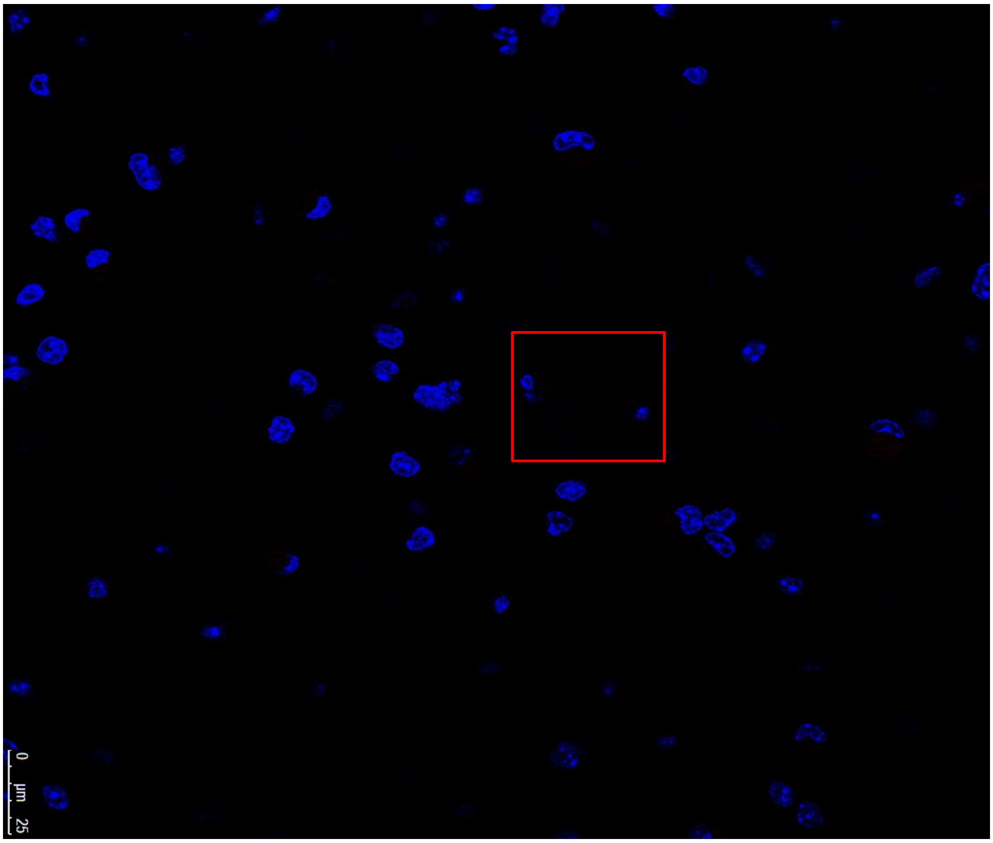

Figure 3A

- Media
- CD11b

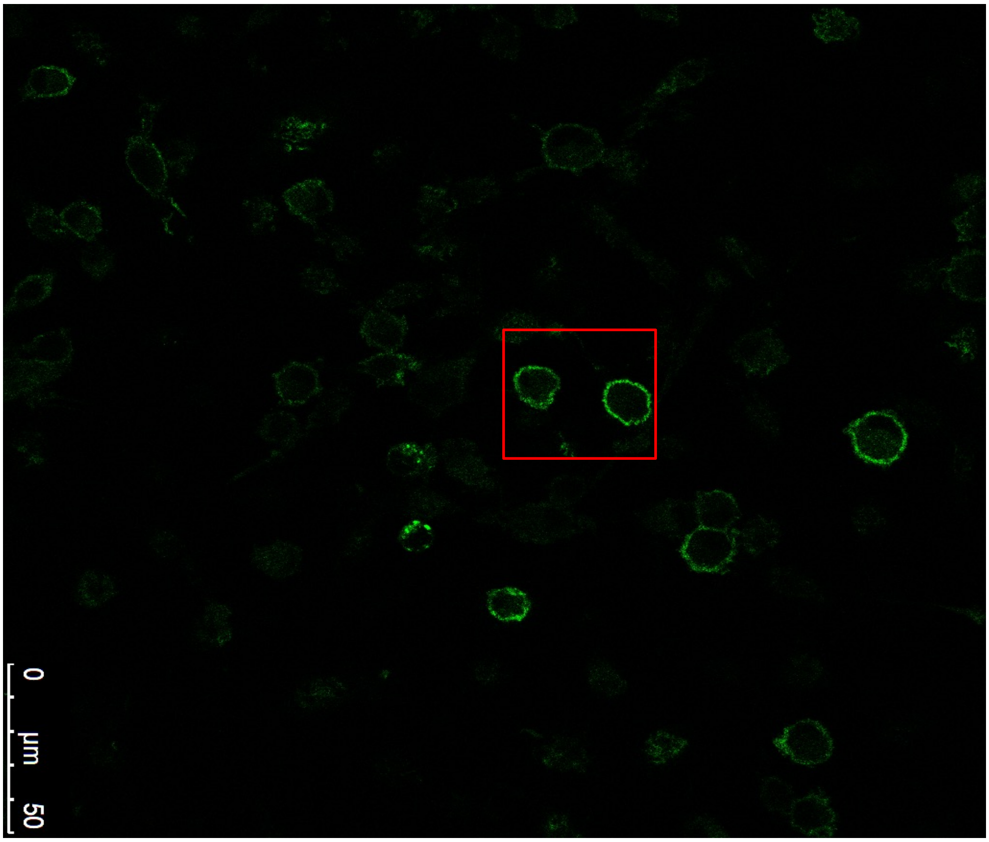

Figure 3A

- Media
- Single cell

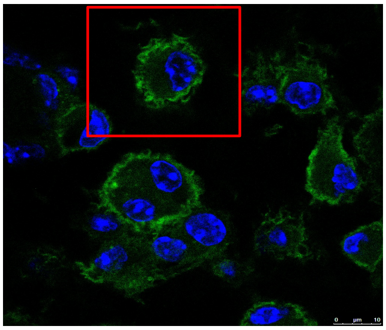

Figure 3A

- AF568-Lec.
- Merge/DAPI

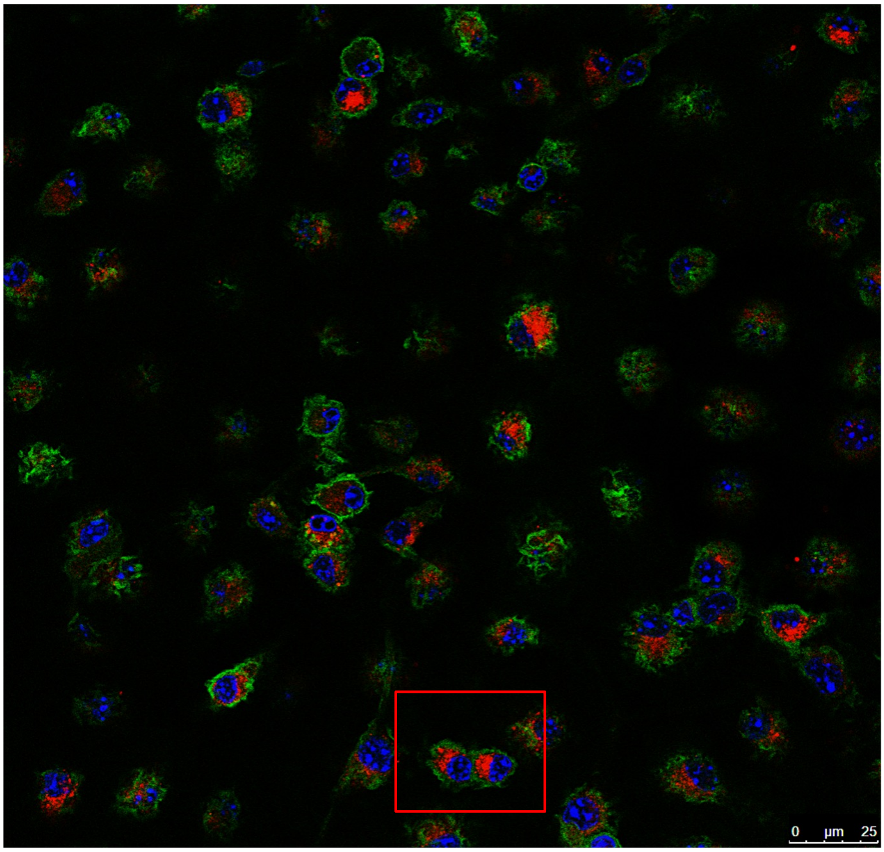

Figure 3A

- AF568-Lec.
- Lec.+ /DAPI

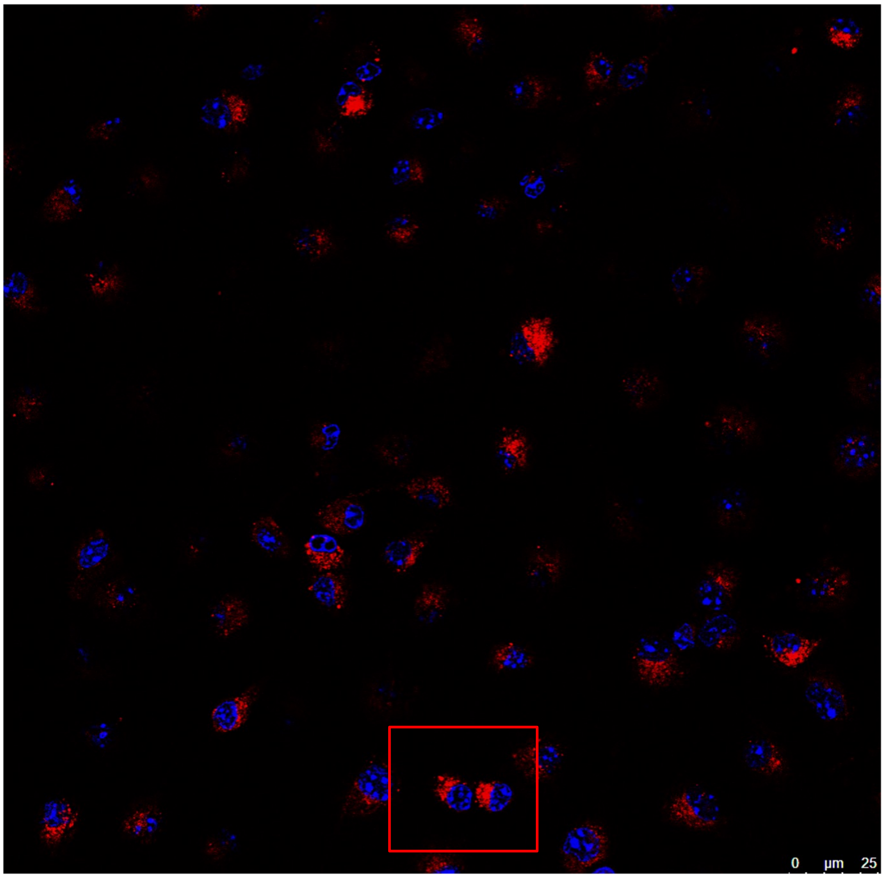

Figure 3A

- AF568-Lec.
- CD11b

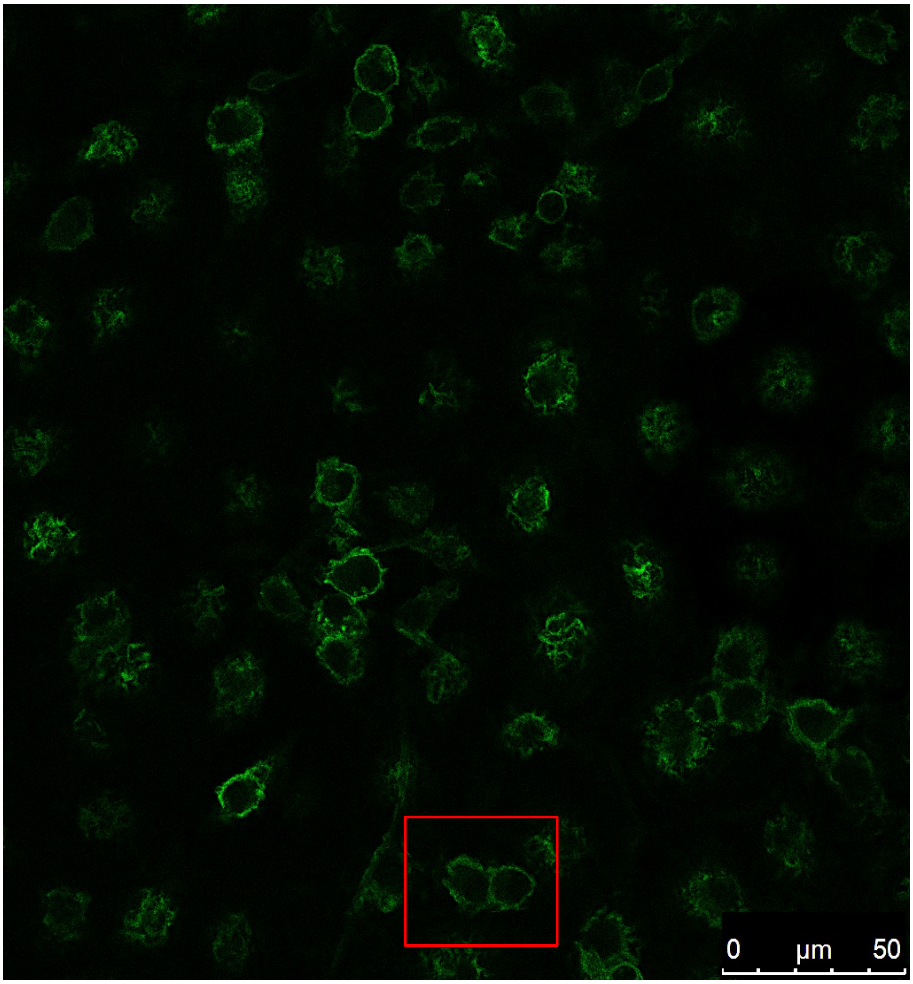

Figure 3A

- AF568-Lec.
- Single cell

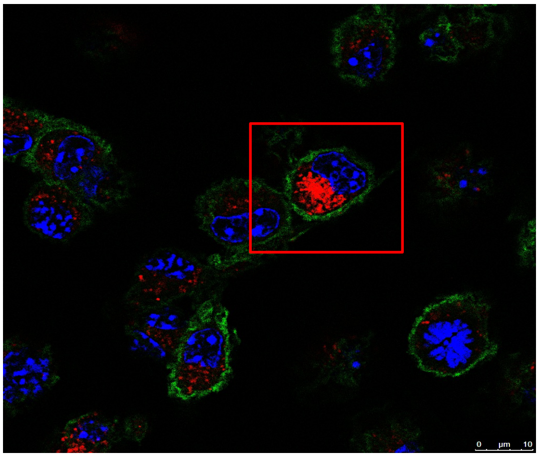

Figure 3A

- Med.+Cyto D
- Merge/DAPI

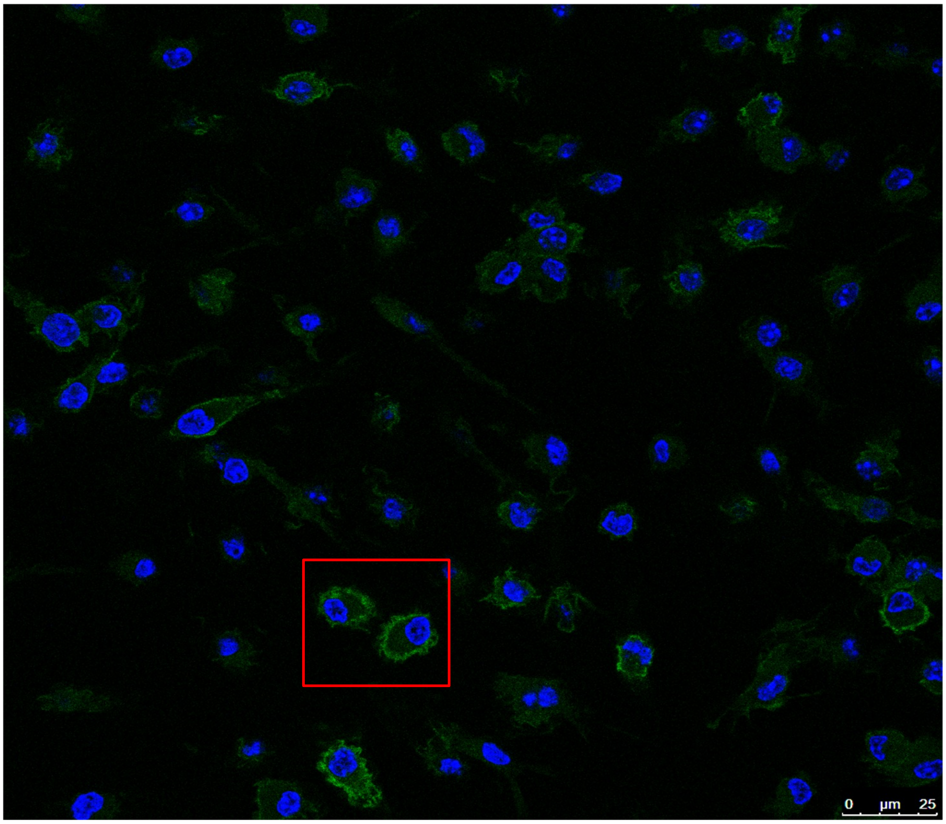

Figure 3A

- Med.+Cyto D
- Lec.+DAPI

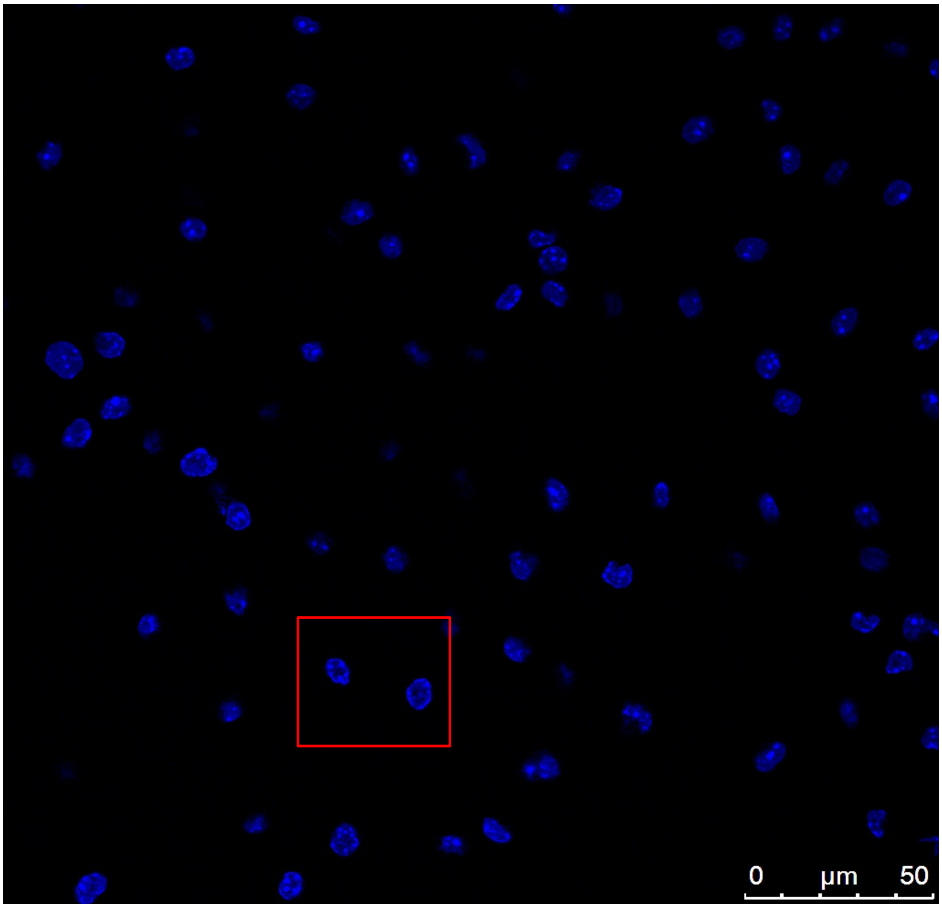

Figure 3A

- Med.+Cyto D
- CD11b

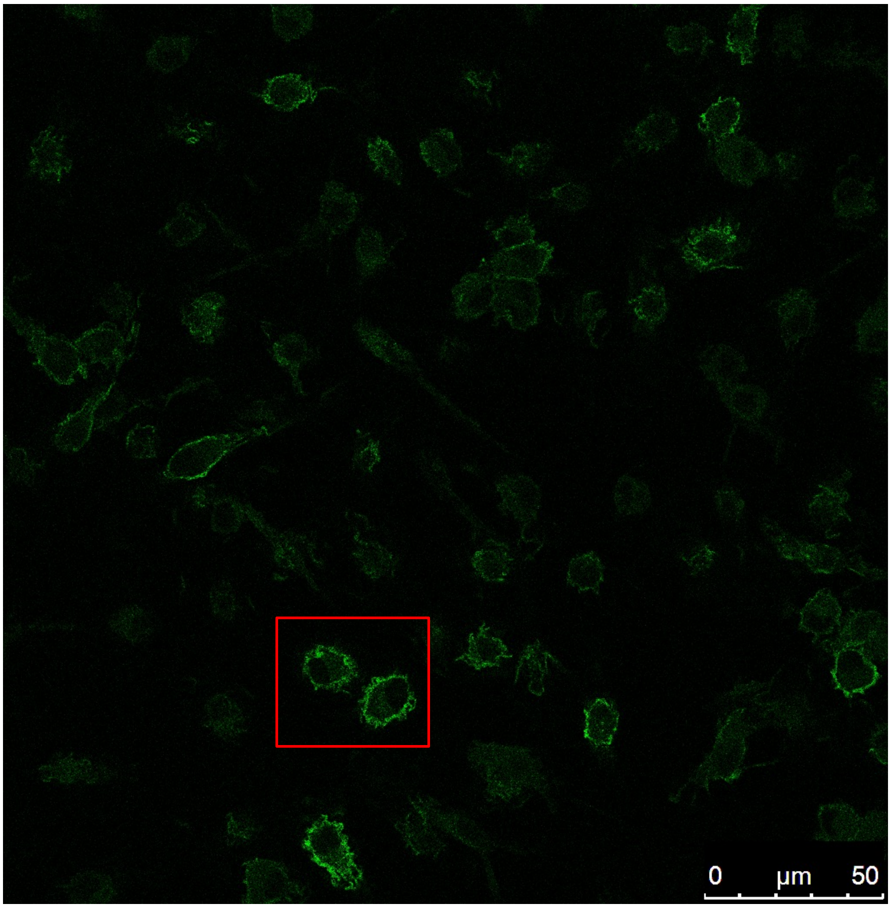

Figure 3A

- Med.+CytoD
- Single cell

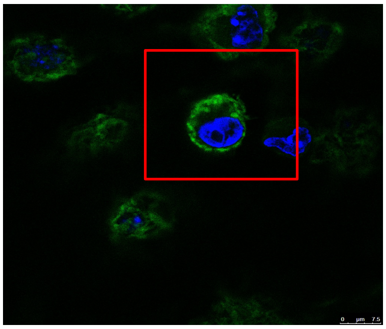

Figure 3A

- AF568-Lec.+CytoD
- Merge/DAPI

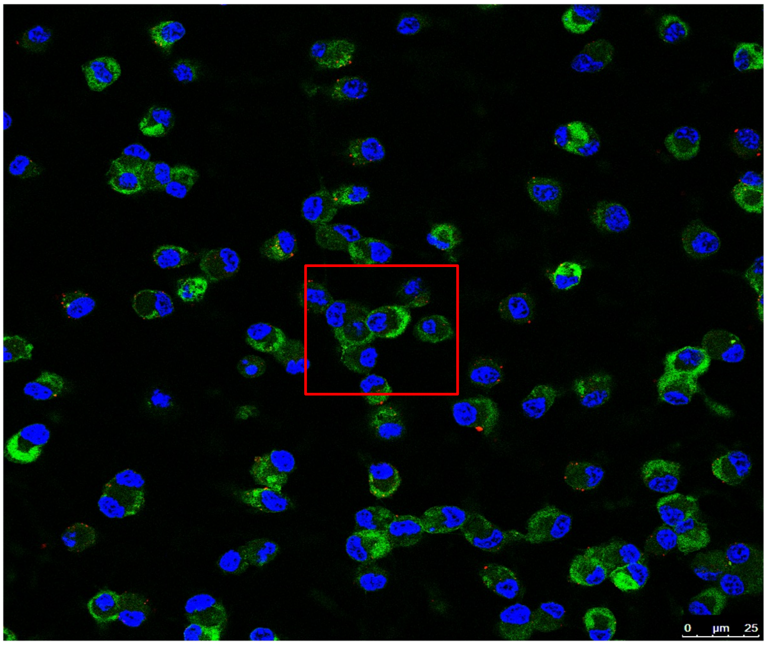

Figure 3A

- AF568-Lec.+CytoD
- Lec.+DAPI

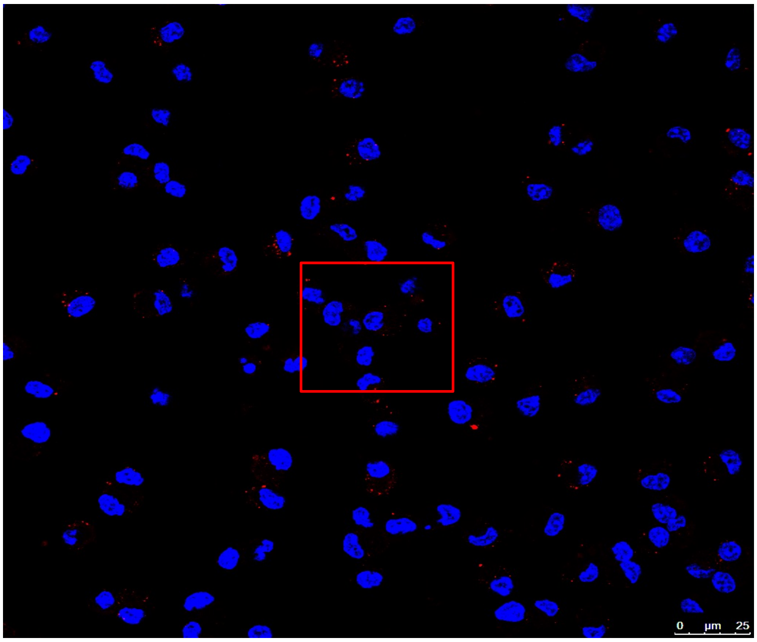

Figure 3A

- AF568-Lec+CytoD.
- CD11b

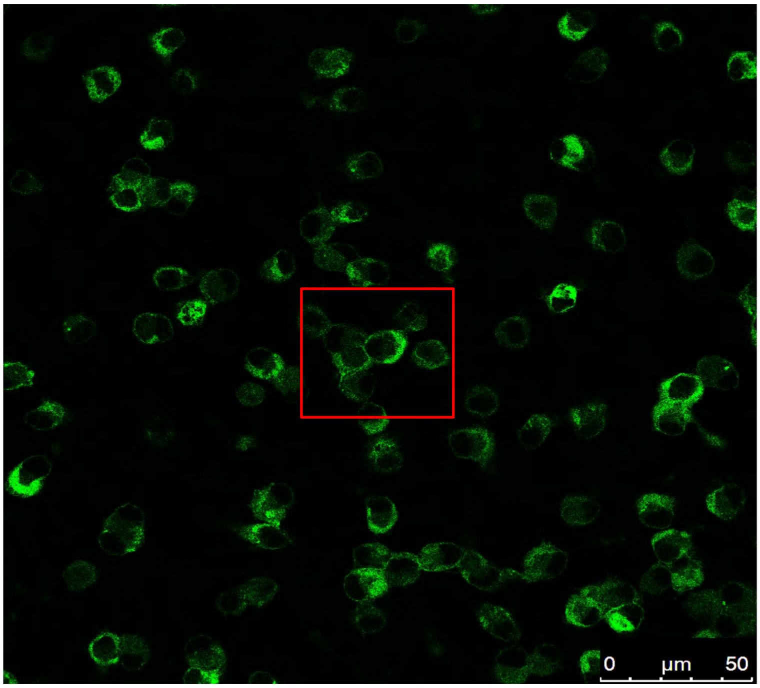

Figure 3A

- AF568-Lec.+CytoD
- Single cell

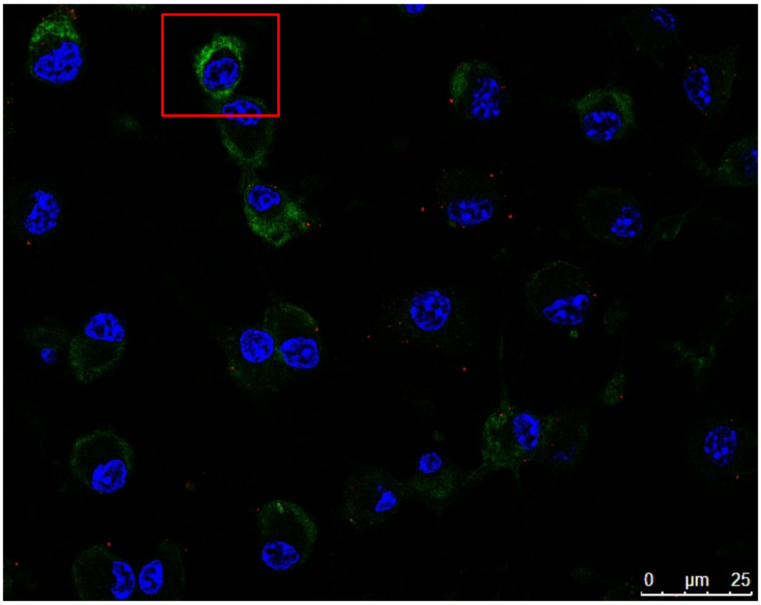

Figure 3A

- Med.+MCD
- Merge/DAPI

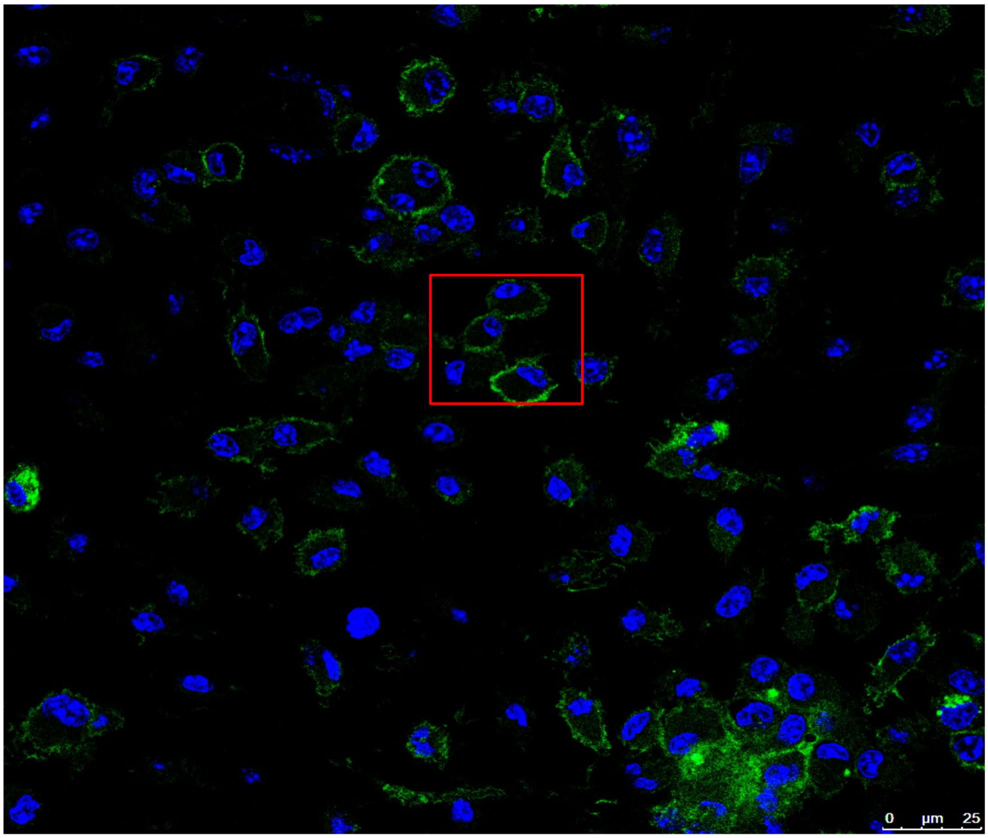

Figure 3A

- Med.+MCD
- Lec.+DAPI

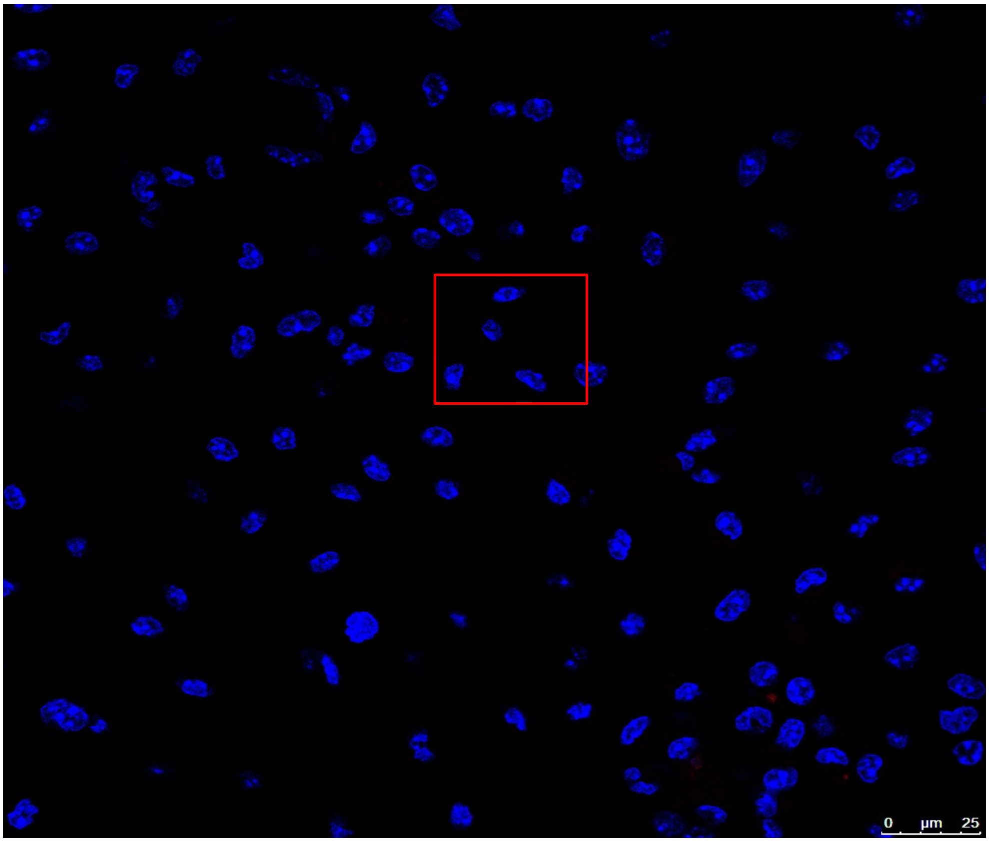

Figure 3A

- Med. + MCD
- CD11b

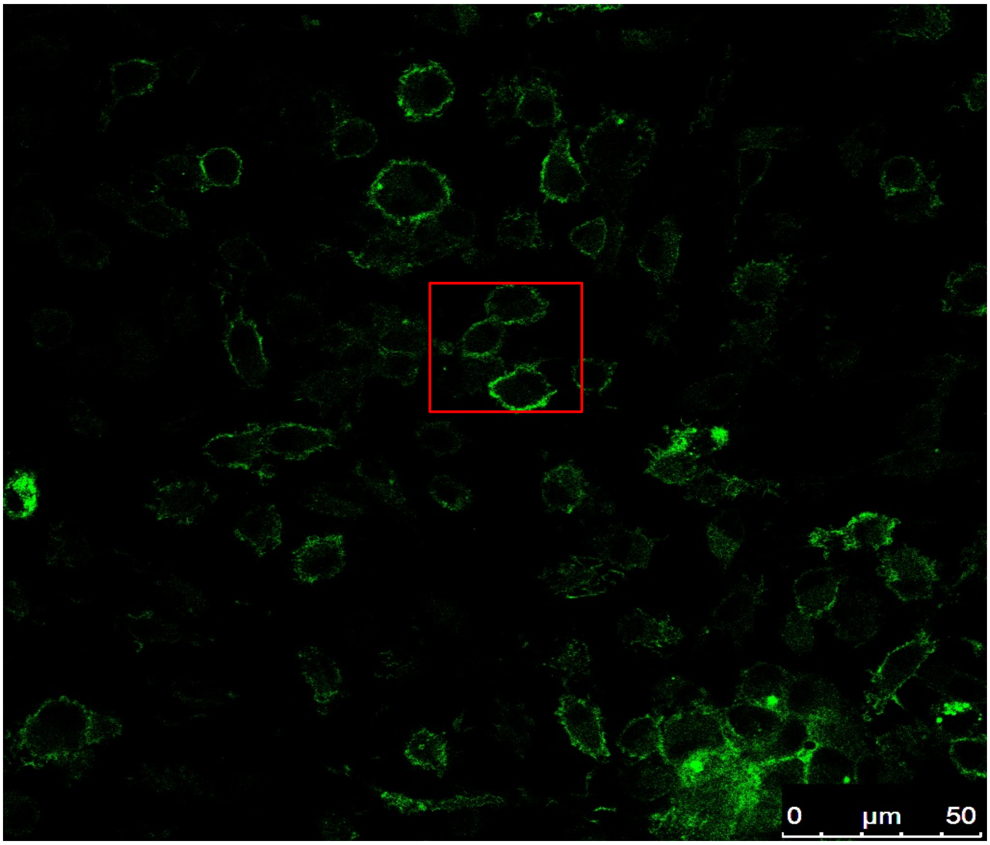

Figure 3A

- Med. + MCD
- Single cell

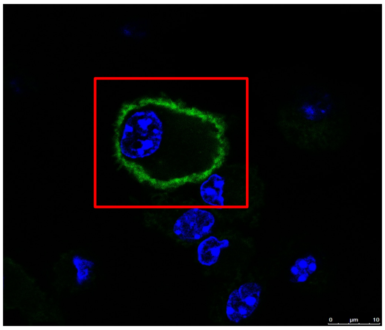

Figure 3A

- AF568-Lec+MCD.
- CD11b

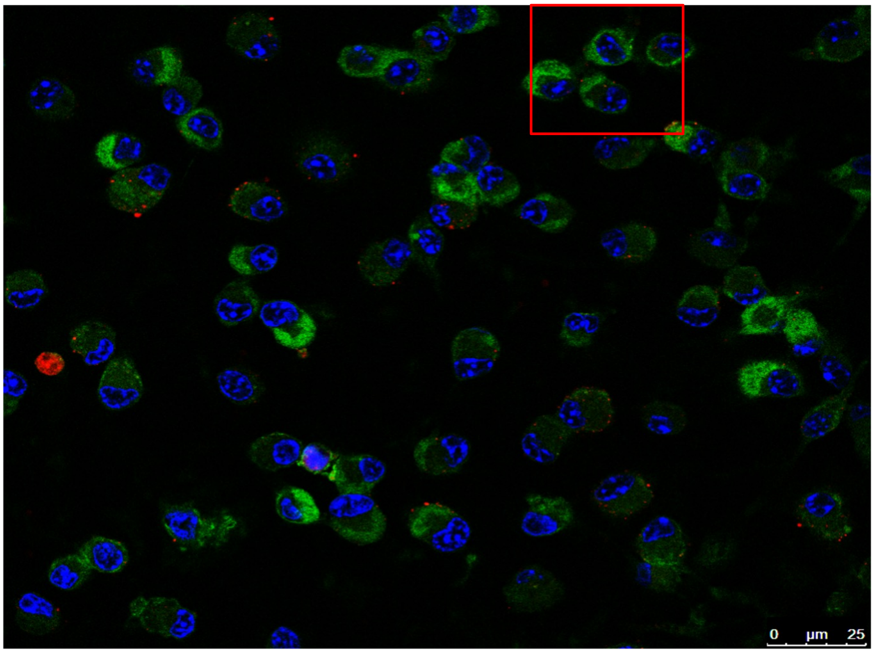

Figure 3A

- AF568-Lec+MCD.
- CD11b

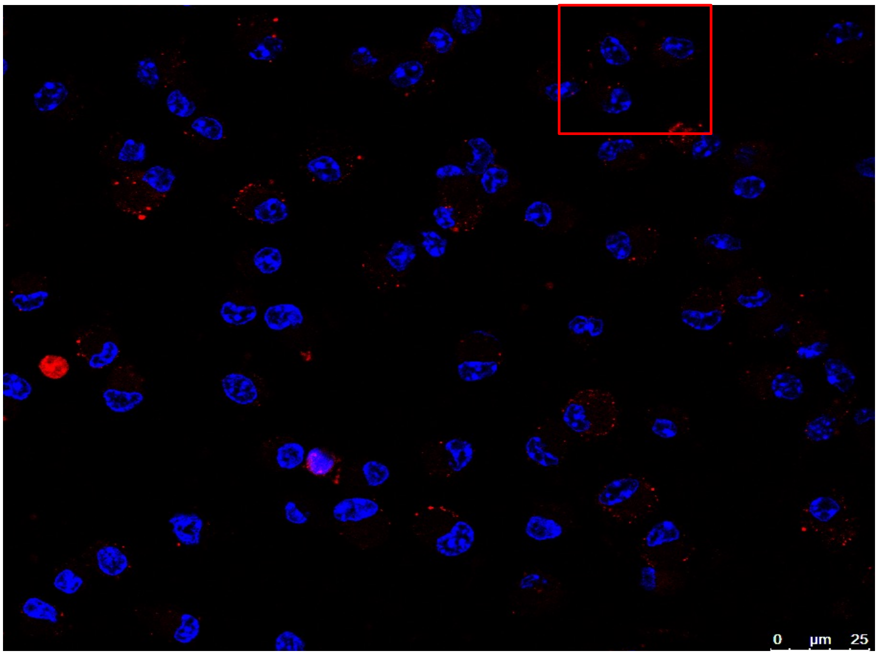

Figure 3A

- AF568-Lec+MCD.
- CD11b

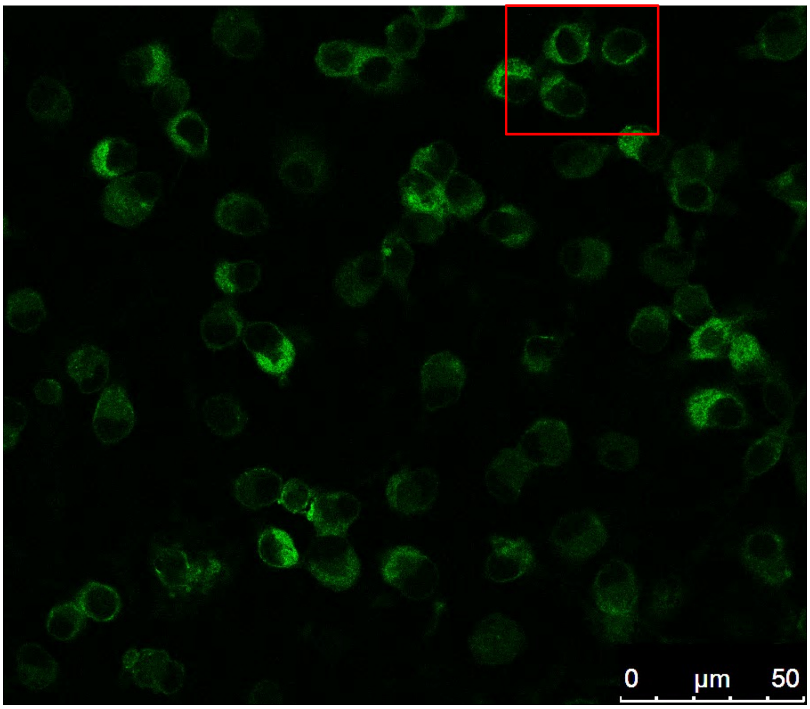

Figure 3A

- AF568-Lec.+MCD
- Single cell

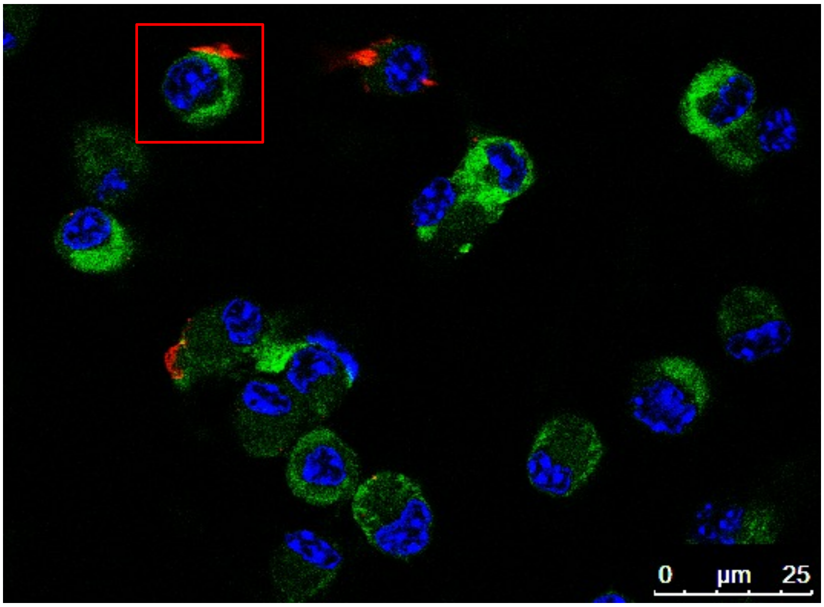

Supplement: Supplementary file 7 — Source Data for Figure 3 [file EMBR-24-e54600-s011.zip › Figure 3/Fig 3A confocal microscopy.pdf]
