# Supplementary figures and images for: Clostridium perfringens virulence factors are nonredundant activators of the NLRP3 inflammasome
Source: EMBO Rep. 2023 Apr 19;24(6):e54600. doi: 10.15252/embr.202254600 (PMC10240202; doi:10.15252/embr.202254600)

**Figure 3C**

- WT BMDMs
- Media, lecithinase

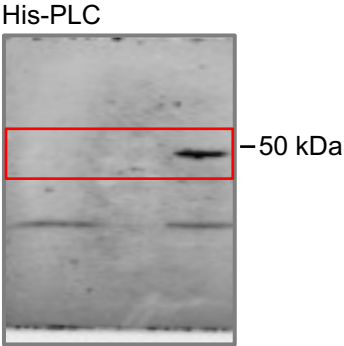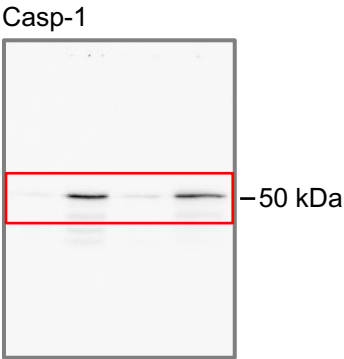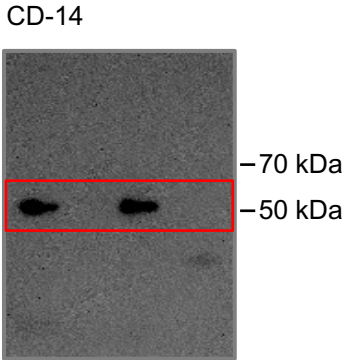

Supplement: Supplementary file 7 — Source Data for Figure 3 [file EMBR-24-e54600-s011.zip › Figure 3/Fig 3C western blot.pdf]

**Figure 3D**  
➤ Untreated/MCD-treated WT BMDMs  
➤ Media, lecithinase, PFO, nigericin

Caspase-1

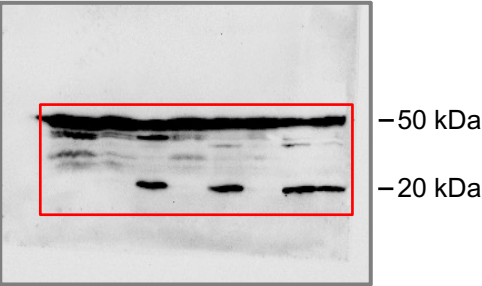

GSDMD

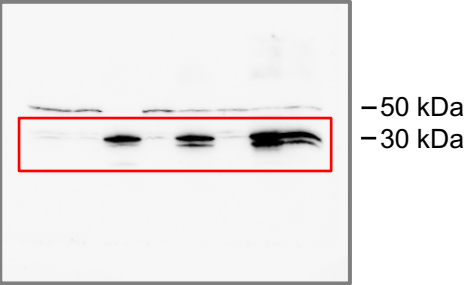

Supplement: Supplementary file 7 — Source Data for Figure 3 [file EMBR-24-e54600-s011.zip › Figure 3/Fig 3D western blot.pdf]

**Figure 4B**  
➤ Untreated BMDMs (Med.)

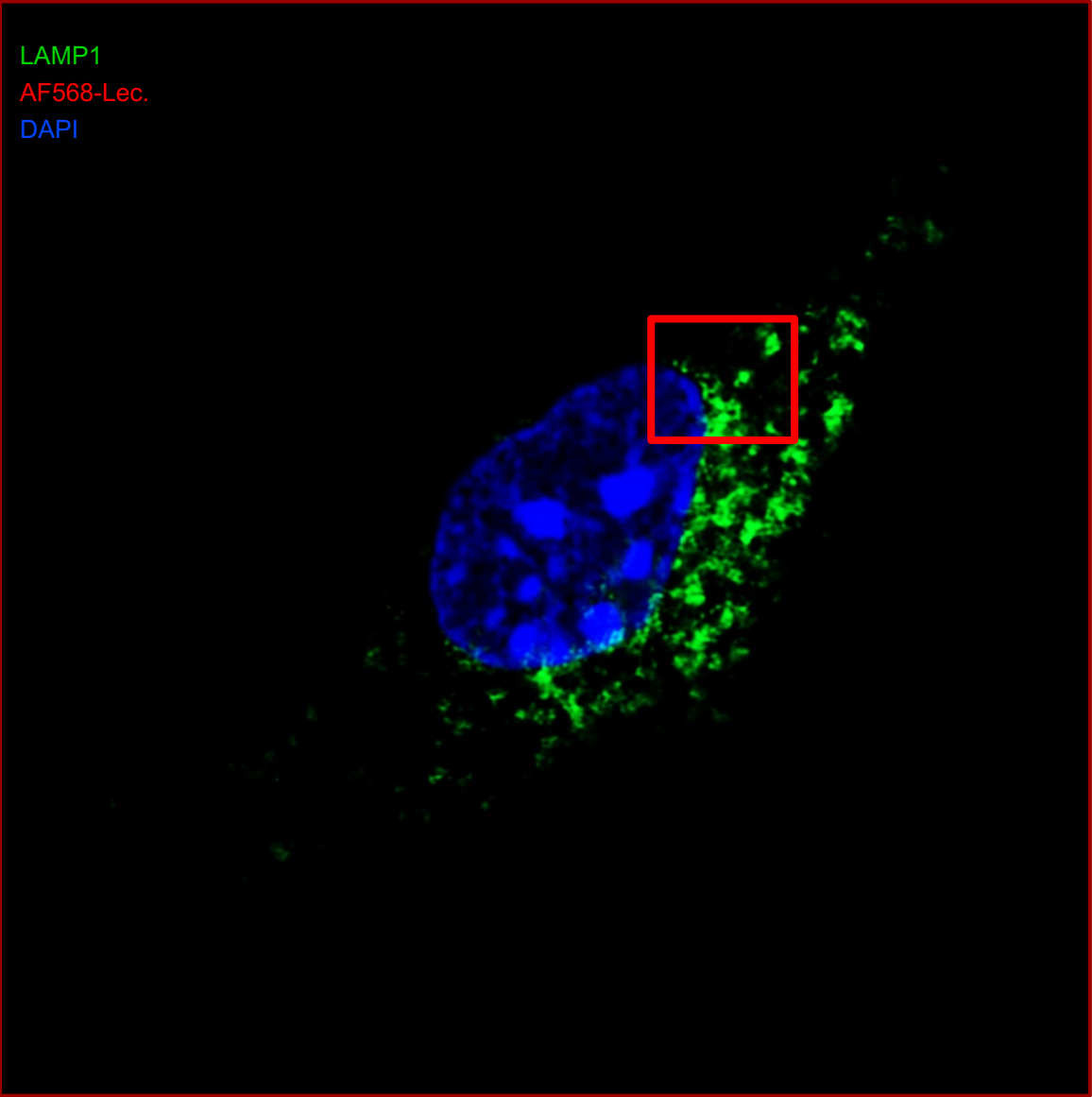

**Figure 4B**

- AF568-Lec. treated BMDMs
- Merge/DAPI

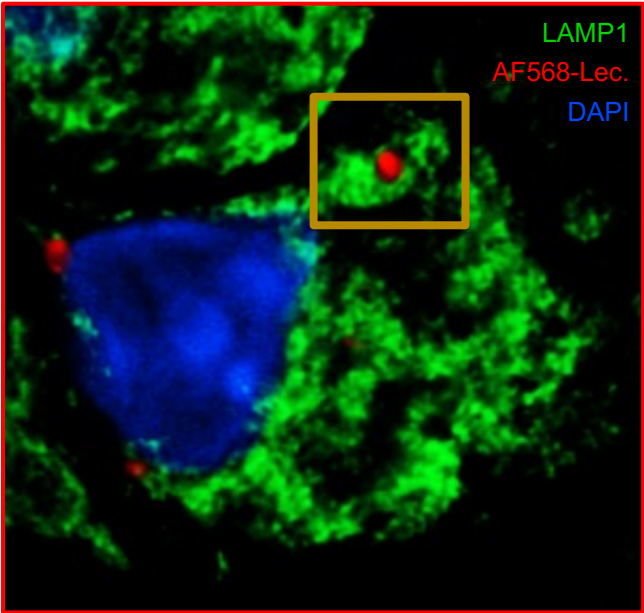

Supplement: Supplementary file 8 — Source Data for Figure 4 [file EMBR-24-e54600-s004.zip › Figure 4/Figure 4B confocal microscopy.pdf]

**Figure 4A**  
➤ Untreated BMDMs (Med.)

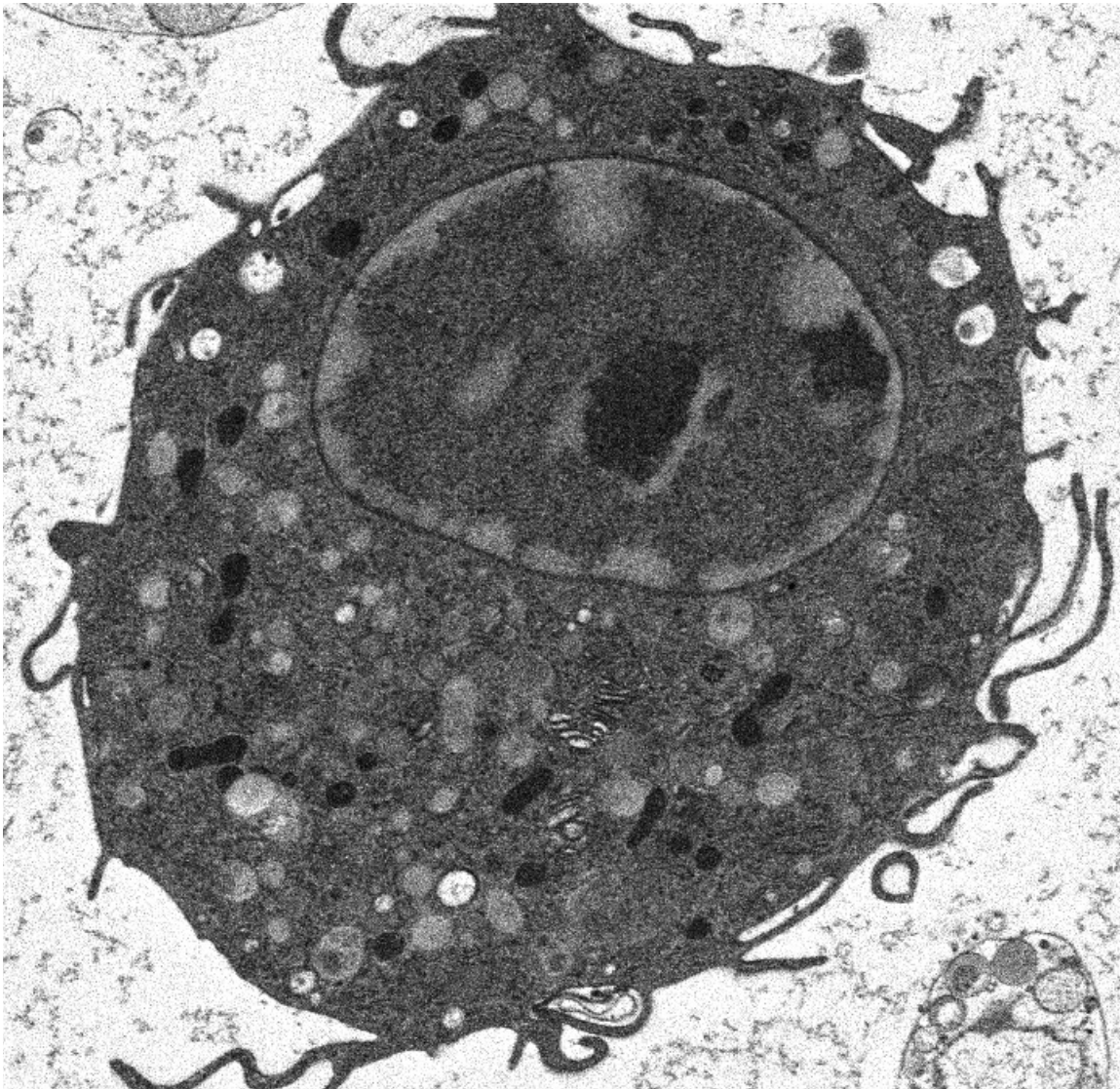

**Figure 4A**  
➤ AF-568 Lecithinase treated BMDMs

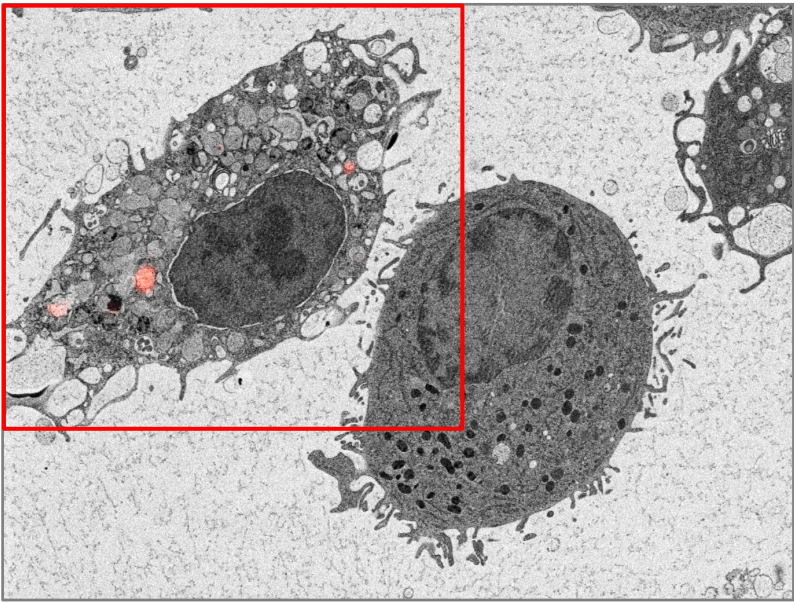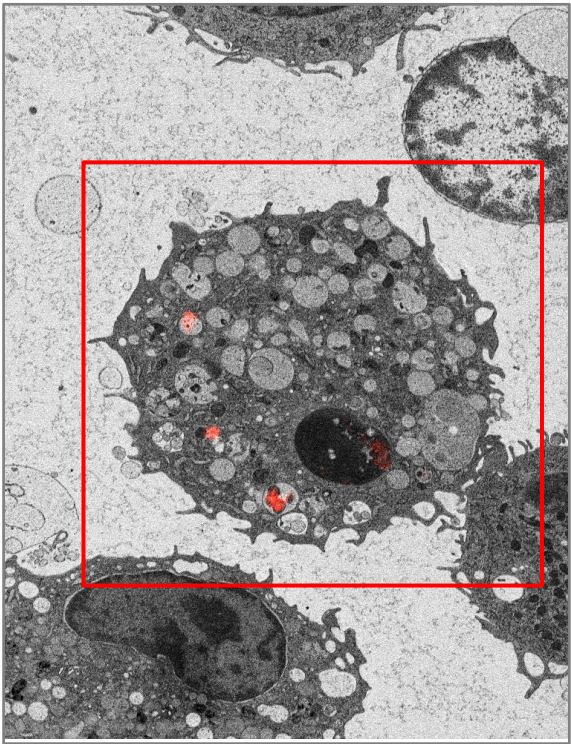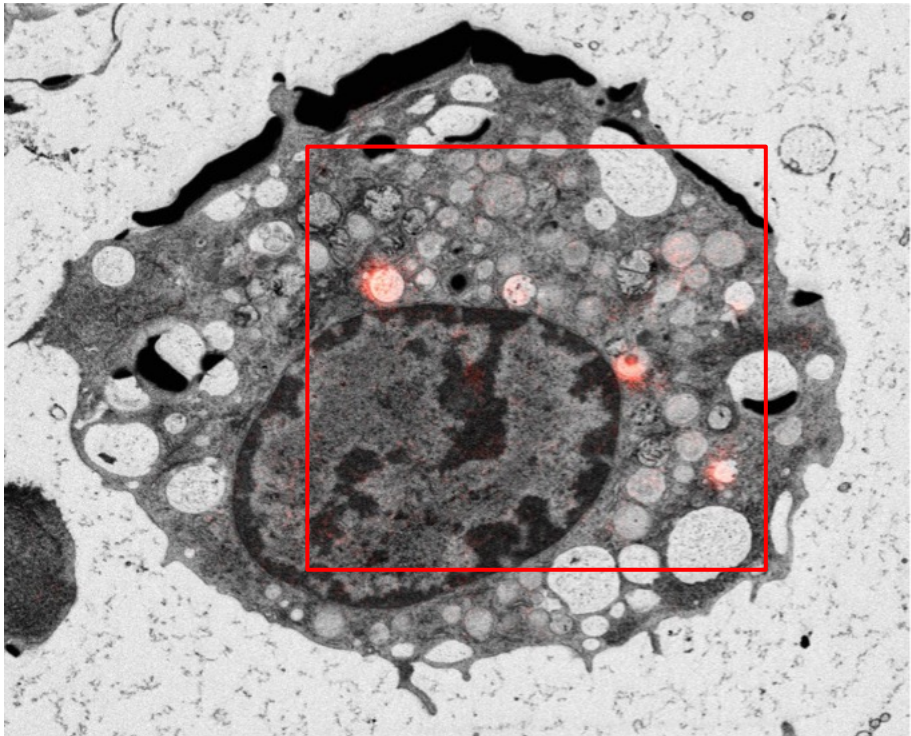

Supplement: Supplementary file 8 — Source Data for Figure 4 [file EMBR-24-e54600-s004.zip › Figure 4/Figure 4A CLEM.pdf]

**Figure 5A**  
➤ Untreated/BafA-treated WT BMDMs  
➤ Media, lecithinase, nigericin

Caspase-1

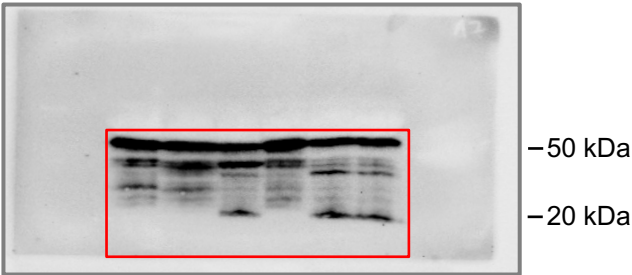

GSDMD

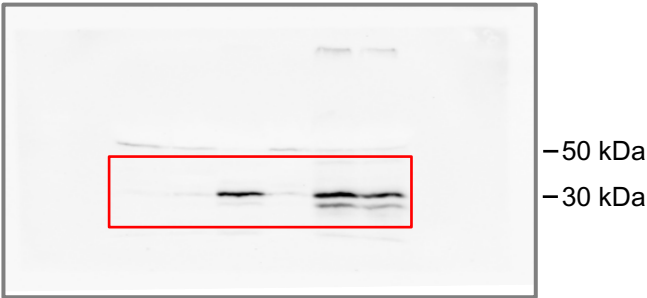

Supplement: Supplementary file 9 — Source Data for Figure 5 [file EMBR-24-e54600-s010.zip › Figure 5/Fig 5A western blot.pdf]

**Figure 6B**  
➤ WT, *Gsdmd*<sup>mut/mut</sup> (I105N/I105N) BMDMs  
➤ Media, lecithinase, LPS transfection

Caspase-1

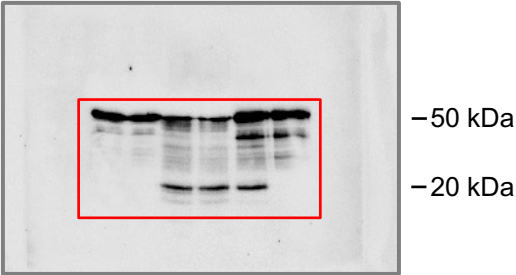

GSDMD

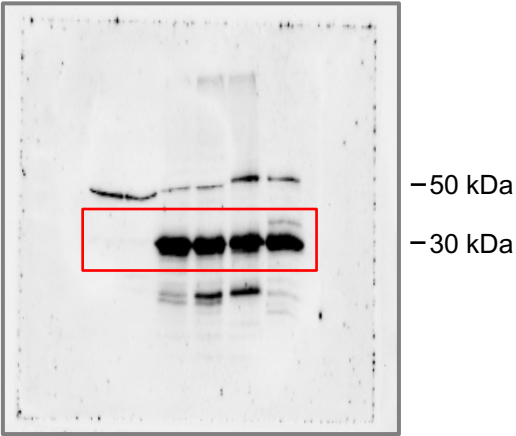

Supplement: Supplementary file 10 — Source Data for Figure 6 [file EMBR-24-e54600-s009.zip › Figure 6/Fig 6B western blot.pdf]

**Figure 6J**  
➤ WT, *Ninj1*<sup>-/-</sup> BMDMs  
➤ Media, lecithinase, LPS transfection

Caspase-1

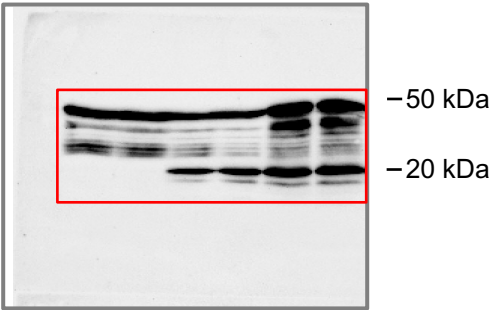

GSDMD

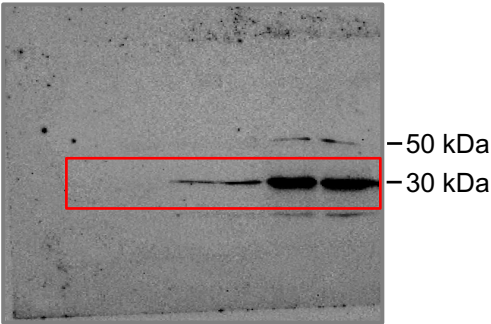

Ninj1

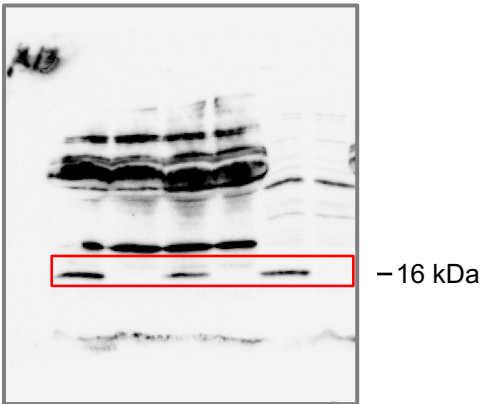

Supplement: Supplementary file 10 — Source Data for Figure 6 [file EMBR-24-e54600-s009.zip › Figure 6/Fig 6J western blot.pdf]

**Figure 6D**  
➤ WT, *Gsdmd*<sup>-/-</sup> BMDMs  
➤ Media, lecithinase, LPS transfection

Caspase-1

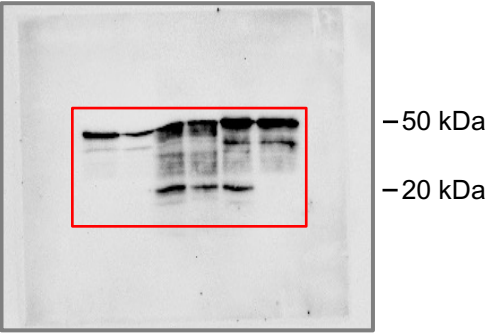

GSDMD

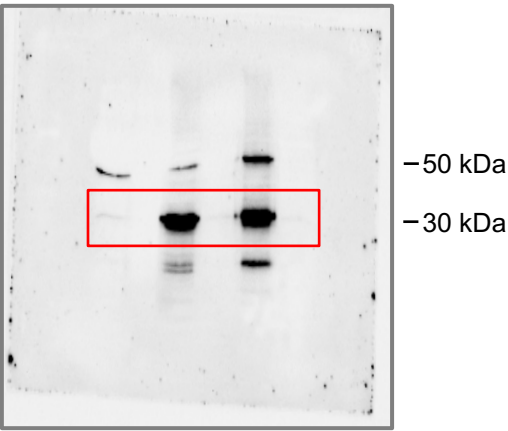

Supplement: Supplementary file 10 — Source Data for Figure 6 [file EMBR-24-e54600-s009.zip › Figure 6/Fig 6D western blot.pdf]
