# Supplementary material for: Clostridium perfringens virulence factors are nonredundant activators of the NLRP3 inflammasome
Source: EMBO Rep. 2023 Apr 19;24(6):e54600. doi: 10.15252/embr.202254600 (PMC10240202; doi:10.15252/embr.202254600)

Figure 5C
- Untreated/NH_4Cl-treated WT BMDMs
- Media, lecithinase, *F. novicida,* nigericin

Caspase-1

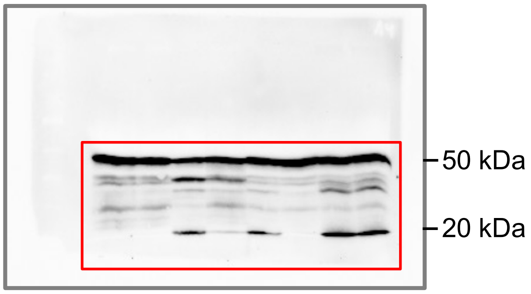

GSDMD

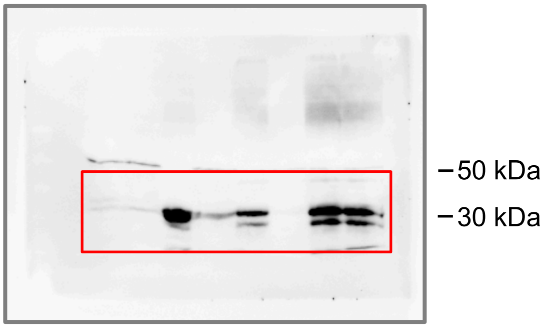

Supplement: Supplementary file 9 — Source Data for Figure 5 [file EMBR-24-e54600-s010.zip › Figure 5/Fig 5C western blot.pdf]
