# Supplementary material for: Clostridium perfringens virulence factors are nonredundant activators of the NLRP3 inflammasome
Source: EMBO Rep. 2023 Apr 19;24(6):e54600. doi: 10.15252/embr.202254600 (PMC10240202; doi:10.15252/embr.202254600)

Appendix Figure S2B

- WT BMDMs
- Media, lecithinase and heat-inactivated lecithinase

Caspase-1

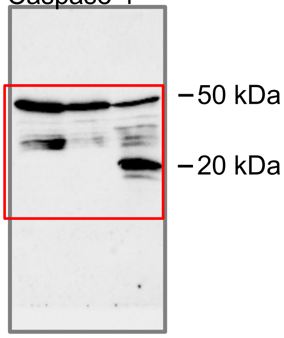

GSDMD

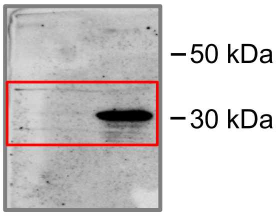

Supplement: Supplementary file 3 — Source Data for Expanded View and Appendix [file EMBR-24-e54600-s002.zip › Appendix_Figures_Source_Data.zip!/Appendix Figures Source Data/Appendix Fig S2B western blot.pdf]
