# Supplementary material for: Clostridium perfringens virulence factors are nonredundant activators of the NLRP3 inflammasome
Source: EMBO Rep. 2023 Apr 19;24(6):e54600. doi: 10.15252/embr.202254600 (PMC10240202; doi:10.15252/embr.202254600)

Figure EV3A

- Untreated/MCC950-treated WT BMDMs
- Media, lecithinase, ATP

Caspase-1

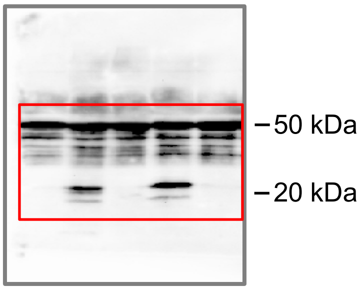

GSDMD

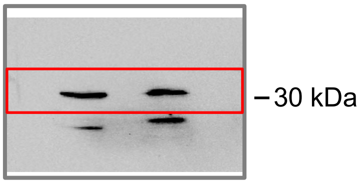

Supplement: Supplementary file 3 — Source Data for Expanded View and Appendix [file EMBR-24-e54600-s002.zip › EV_Figures_Source_Data.zip!/EV Figure 3 /Fig EV3A western blot.pdf]
