# Supplementary material for: Clostridium perfringens virulence factors are nonredundant activators of the NLRP3 inflammasome
Source: EMBO Rep. 2023 Apr 19;24(6):e54600. doi: 10.15252/embr.202254600 (PMC10240202; doi:10.15252/embr.202254600)

Figure EV2A

- Untreated BMDMs (Med.)

WT

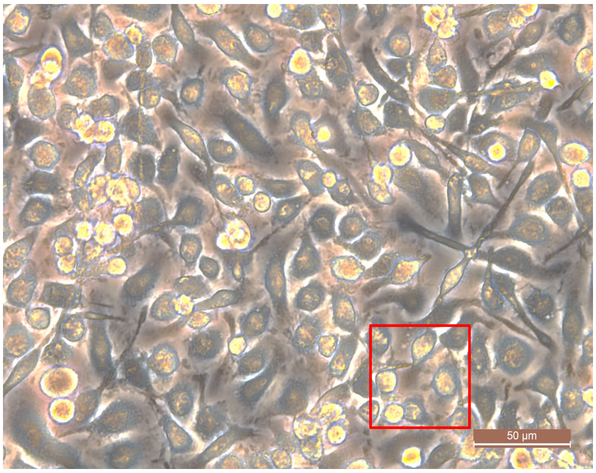

Casp1/11$^{-/-}$

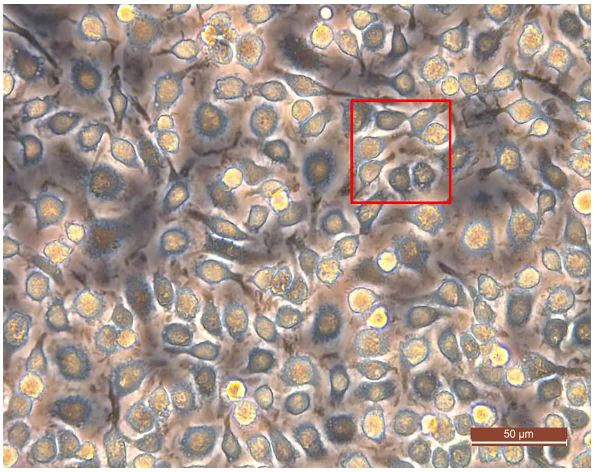

Nlrp3$^{-/-}$

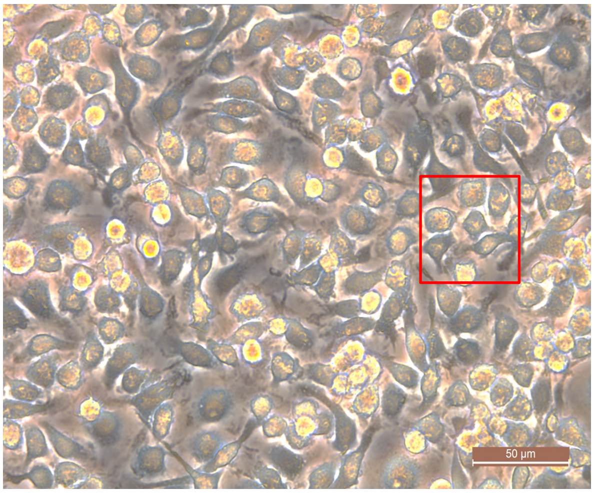

Nlrc4$^{-/-}$

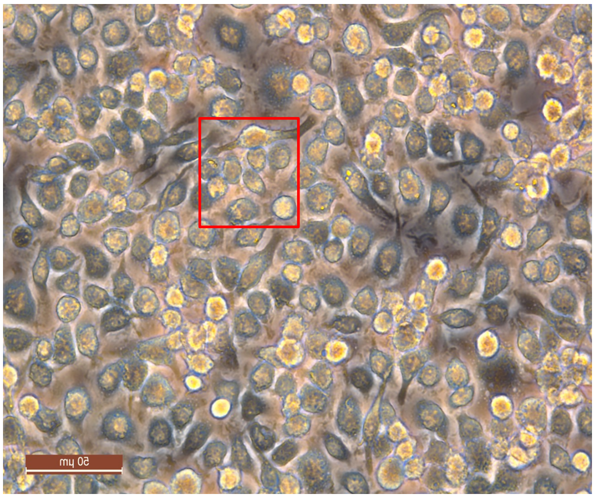

Asc$^{-/-}$

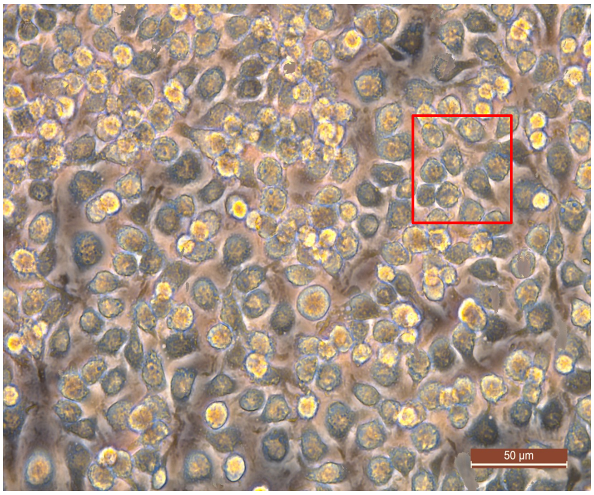

Aim2$^{-/-}$

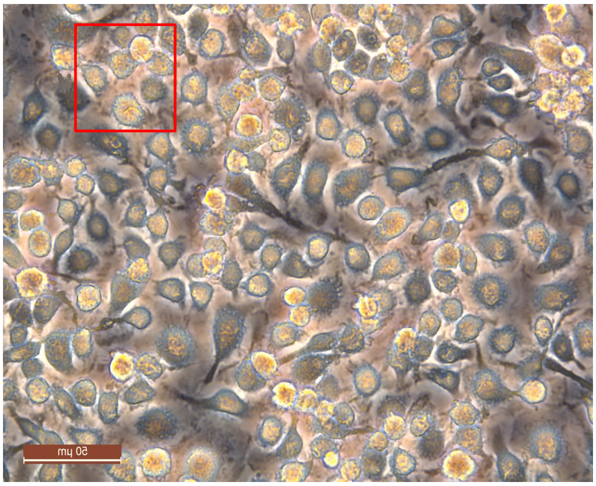

Figure EV2A

- Untreated BMDMs (Med.)

Mefv$^{mut/mut}$

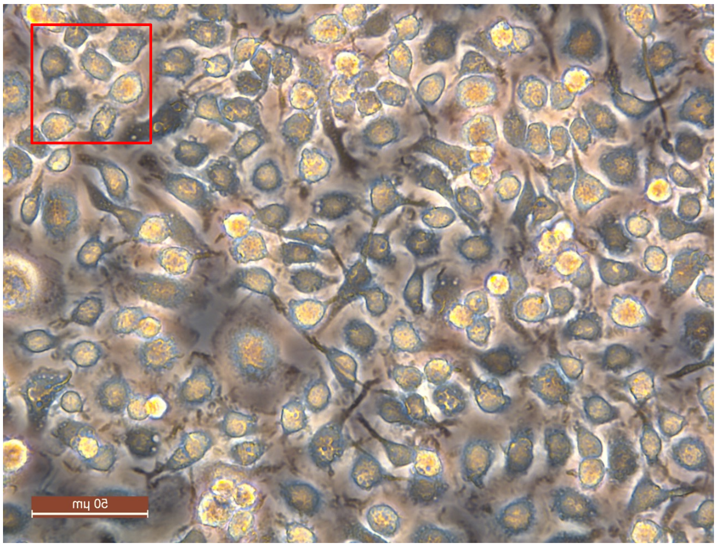

Casp11$^{-/-}$

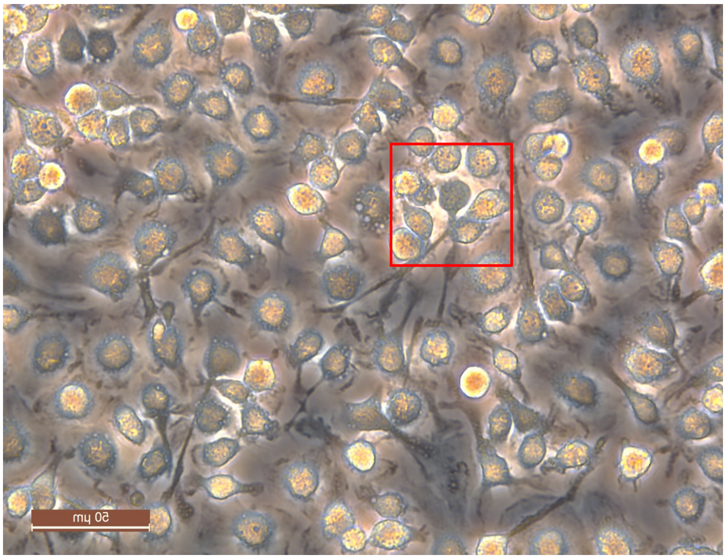

Gsdmd$^{I105N/I105N}$

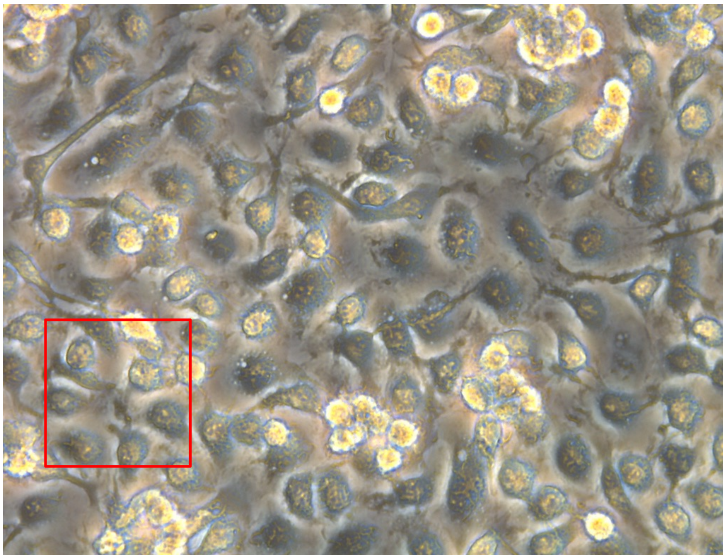

Figure EV2A

➢ Lecithinase treated BMDMs

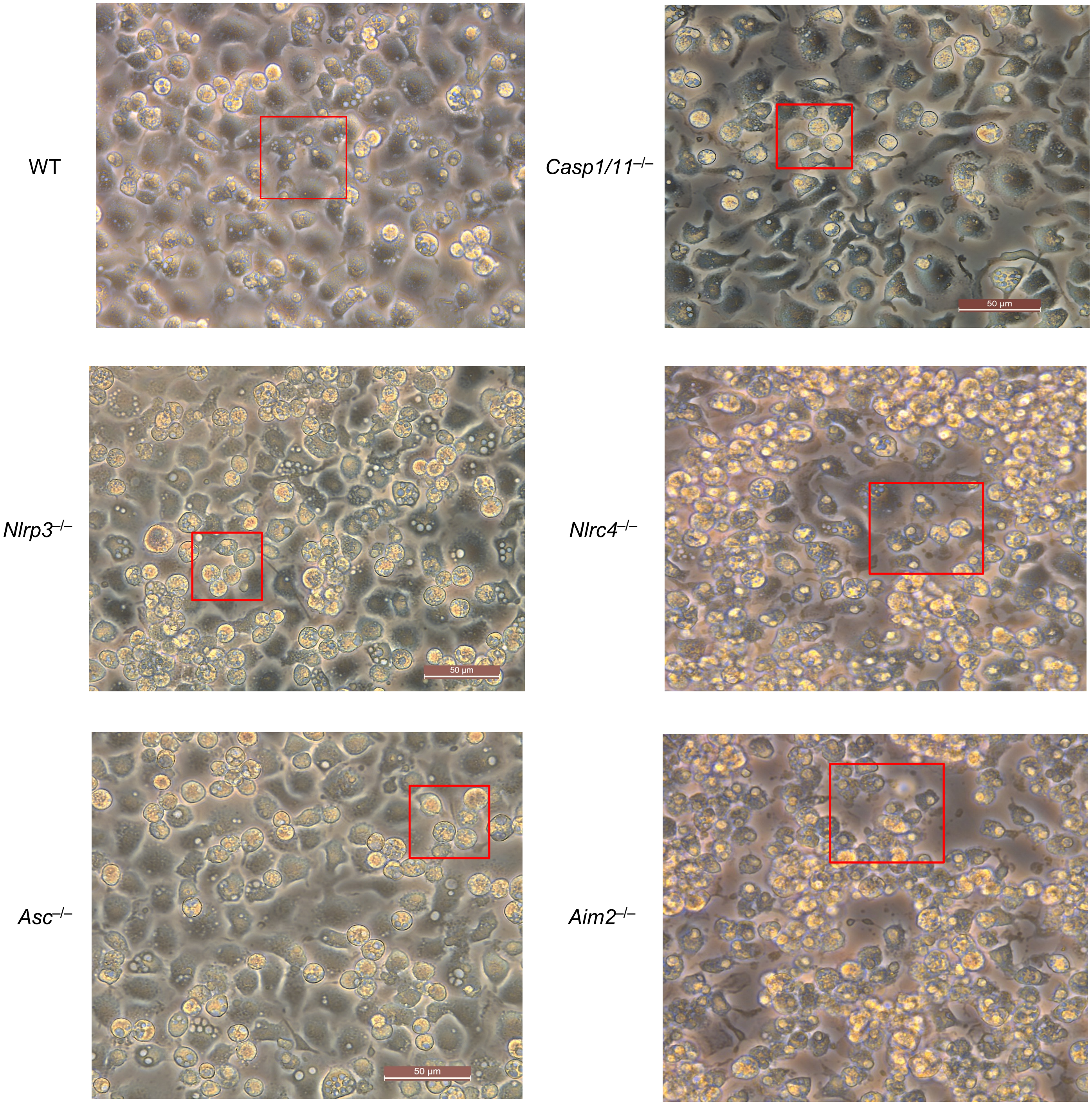

Figure EV2A

➢ Lecithinase treated BMDMs

Mefv$^{mut/mut}$

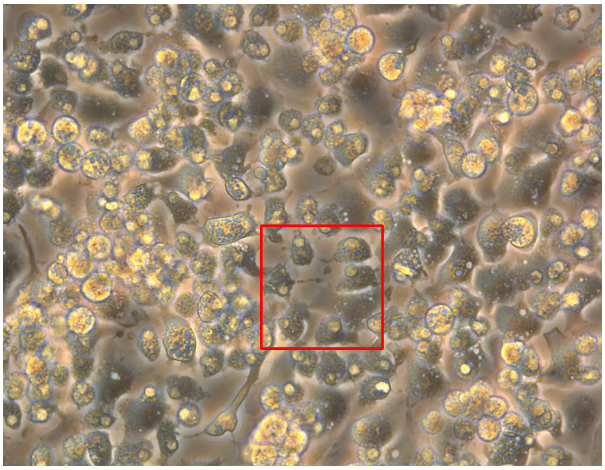

Casp11$^{–/–}$

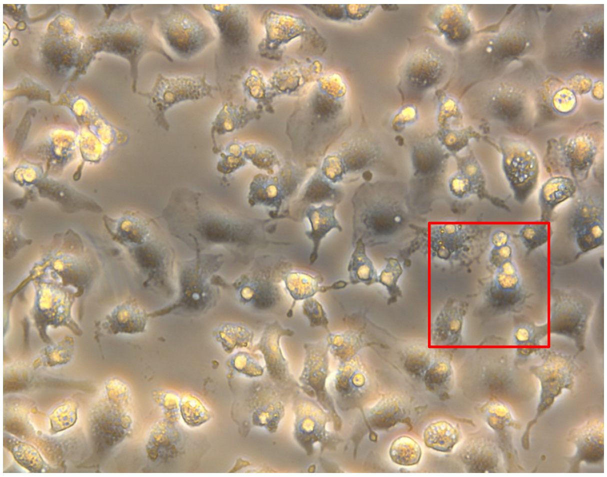

Gsdmd$^{I105N/I105N}$

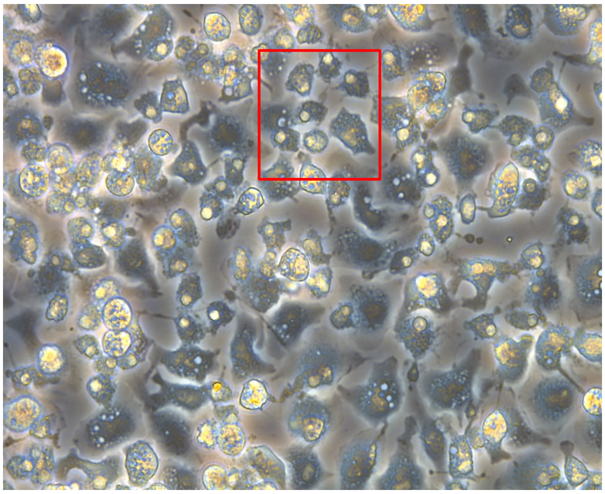

Supplement: Supplementary file 3 — Source Data for Expanded View and Appendix [file EMBR-24-e54600-s002.zip › EV_Figures_Source_Data.zip!/EV Figure 2/EV2A brightfield microscopy.pdf]
